# First records and three new species of the family Symphytognathidae (Arachnida, Araneae) from Thailand, and the circumscription of the genus *Crassignatha* Wunderlich, 1995

**DOI:** 10.3897/zookeys.1012.57047

**Published:** 2021-01-26

**Authors:** Francisco Andres Rivera-Quiroz, Booppa Petcharad, Jeremy A. Miller

**Affiliations:** 1 Department of Terrestrial Zoology, Understanding Evolution group, Naturalis Biodiversity Center, Darwinweg 2, 2333CR Leiden, the Netherlands Naturalis Biodiversity Center Leiden Netherlands; 2 Institute for Biology Leiden (IBL), Leiden University, Sylviusweg 72, 2333BE Leiden, the Netherlands Leiden University Leiden Netherlands; 3 Faculty of Science and Technology, Thammasat University, Rangsit, Pathum Thani, 12121 Thailand Thammasat University Pathum Thani Thailand

**Keywords:** 3D reconstruction, *
Anapistula
*, Borneo, computed tomography, micro-CT, *
Patu
*, Sabah, Symphytognathoids

## Abstract

The family Symphytognathidae is reported from Thailand for the first time. Three new species: *Anapistula choojaiae***sp. nov.**, *Crassignatha seeliam***sp. nov.**, and *Crassignatha seedam***sp. nov.** are described and illustrated. Distribution is expanded and additional morphological data are reported for *Patu shiluensis* Lin & Li, 2009. Specimens were collected in Thailand between July and August 2018. The newly described species were found in the north mountainous region of Chiang Mai, and *Patu shiluensis* was collected in the coastal region of Phuket. DNA sequences are provided for all the species here studied. The relations of these symphytognathid species were tested using previously published phylogenetic analyses on micro orb-weavers. Also, we used micro CT analysis to build 3D models of the male genitalia and somatic characters of two species of *Crassignatha* Wunderlich, 1995. The molecular phylogeny and 3D models were used to discuss the taxonomy and circumscription of the currently valid symphytognathid genera, with focus on *Crassignatha* and *Patu* Marples, 1951. Based on this, three new combinations are suggested: *Crassignatha bicorniventris* (Lin & Li, 2009), **comb. nov.**, *Crassignatha quadriventris* (Lin & Li, 2009), **comb. nov.**, and *Crassignatha spinathoraxi* (Lin & Li, 2009), **comb. nov.** A new record of *Crassignatha danaugirangensis*Miller et al. 2014 is reported from Brunei.

## Introduction

The family Symphytognathidae includes some of the tiniest spiders known. According to a recent “Spider World Record” study (Mammola et al. 2017), this family holds the records for the smallest female, smallest male and smallest web. The Symphytognathidae has traditionally been put together with other small size araneoids (Anapidae, Mysmenidae, and Theridiosomatidae, sometimes with synaphrids and micropholcommatids) in a group informally called the symphytognathoids (Griswold et al. 1998; Hormiga and Griswold 2014). Although phylogenetic relationships among the Symphytognathidae have not been directly studied, some representatives have been used as part of other phylogenetic studies targeting the family Mysmenidae (Lopardo et al. 2011; Feng et al. 2019), as well as a broad scope analysis of the whole order Araneae (Wheeler et al. 2017; Kulkarni et al. 2020). Symphytognathids can be separated from other relatives by the following combination of characters: the loss of the posterior median eyes, reducing eye number to six (with the further loss of the anterior median eyes in the case of the four-eyed genus *Anapistula*), fusion of the chelicerae (but see below), extreme reduction or loss of female pedipalp, the labium being much wider than long, loss of the colulus, sternum broadly truncated posteriorly, the absence of book lungs, and the presence of one or two promarginal cheliceral teeth originating from a common base (Forster and Platnick 1977; Wunderlich 2004; Miller et al. 2009; Lopardo et al. 2011; Hormiga and Griswold 2014).

The family is widespread in the tropics and subtropical regions, with most species described from the southern hemisphere. At present 8 genera and 74 species are recorded worldwide. In Asia, six genera and 29 species have been recorded (WSC 2020). From these, 19 species have been recorded from China (Tong and Li 2006; Lin and Li 2009; Miller et al. 2009; Lin et al. 2013; Lin 2019) and six from South East Asia (Indonesia, Malaysia and Vietnam) (Wunderlich 1995; Harvey 1998; Lin et al. 2009; Miller et al. 2014). Here, the family Symphytognathidae is formally reported from Thailand for the first time, although Lopardo et al. (2011) did include a Thai symphytognathid in their study, designated SYMP-004-THAI, which was later identified as *Crassignatha* (Lopardo, pers. comm.). We describe three new species of the genera *Anapistula* and *Crassignatha* and expand the known distribution of *Patu shiluensis*. We used a combination of newly generated sequences and sequences available in GenBank to build a molecular phylogeny of the Symphytognathidae, and related micro orb-weaver families, in order to test the familial placement of our new species. Additionally, we discuss the taxonomy of the Symphytognathidae with emphasis on the genera *Crassignatha* and *Patu*.

## Materials and methods

### Fieldwork

The symphytognathid specimens reported here were collected in Chiang Mai and Phuket, Thailand, between 16 July and 6 August 2018. All the specimens were captured using methods optimized for ground dwelling spiders: leaf litter sifting, Winkler extractors, pitfall traps and direct collecting on ground, and among sifted leaf litter.

### Molecular data

To test the relationships and position of the novel species within the Symphytognathidae, we selected one specimen from each species we collected and used all four right legs to extracted genomic DNA and sequence six gene fragments: COI, H3, 12S, 16S, 18S, and 28S (primers in Suppl. material 1) following Miller et al. (2010) and Wheeler et al. (2017) protocols. Sequences were edited in Geneious Prime 2020.0.5 and deposited in GenBank; accession numbers are reported in Table 1. We used these sequences and a selection of taxa previously used to test the phylogeny of mysmenid spiders (Lopardo et al. 2011; Feng et al. 2019). In total, 47 species of “symphytognathoids” from the families Anapidae, Mysmenidae, Symphytognathidae and Theridiosomatidae were used. Two more species of Tetragnathidae were used as an outgroup to the symphytognathoids. We used MAFFT v.7.450 online (https://mafft.cbrc.jp/alignment/server/) with default parameters to align the sequences. Matrix was built using in Sequence Matrix v.1.8 (http://www.ggvaidya.com/taxondna/); matrix available in Suppl. material 1. Each locus was treated as a partition and examined with jModelTest2 (Darriba et al. 2012) in CIPRES (Miller et al. 2010) to get the best model fit for each; GTR+I+G was selected in all cases. Our datasets were analyzed using MEGA X (Kumar et al. 2018) for Maximum Parsimony (SPR, default values, bootstrap = 1000); RaXML (Stamatakis 2014) in CIPRES for Maximum Likelihood (GTR, bootstrap = 1000) and MrBayes v. 3.2.6 (Ronquist and Huelsenbeck 2003) in CIPRES for the Bayesian Inference (GTR+I+G, two independent runs with one cold and three heated chains, mcmc = 50,000,000 gen, samplefreq = 1000, burnin = 2500; partitions are indicated in the NEXUS file). The program Tracer v. 1.7.1 (Rambaut et al. 2018) was used to analyze the performance of our BI analyses.

**Table 1.** GenBank accession numbers of DNA sequences generated for the present work.

Species	COI	H3	16s	12s	18s	28s
*Anapistula choojaiae*	MT712393	MT782018	–	MT711286	MT711238	MT711242
*Crassignatha seedam*	MT712396	MT782021	–	–	MT711241	–
*Crassignatha seeliam*	MT712394	MT782019	–	–	MT711239	–
*Patu shiluensis*	MT712395	MT782020	MT711285	–	MT711240	–

### Morphological data

Specimens were photographed with a Nikon DS-Ri2 camera attached to a Leica DM 2500 microscope. Specimens were observed in ethanol using semi-permanent slide preparations (Coddington 1983). Female genitalia were dissected, digested using pancreatin solution (Alvarez-Padilla and Hormiga 2007), and cleared with methyl salicylate. For the 3D scans, whole male spiders were stained in 1% iodine in 70% et-OH for 24 hours. Specimens were fixed in a modified 10 ul pipette tip and scanned using a Zeiss X-radia 520 versa. 3D model and subsequent segmentation of the internal ducts of male pedipalps were done in Avizo 9.5.0. All the specimens have been deposited in the collection of the Naturalis Biodiversity Center, Leiden, the Netherlands. Additionally, two males of *Crassignatha danaugirangensis*Miller et al., 2014, recently collected in Brunei, were analyzed using micro-CT scanning. 3D reconstructions were used to clarify some anatomical details of this species and the genus *Crassignatha*, including the internal and external structure of the male pedipalp, cheliceral armature, and carapace texture.

Nomenclature of the genital structures was based on Harvey (1998) and Lin et al. (2013) for *Anapistula*, and Lin and Li (2009) and Miller et al. (2009) for *Crassignatha* and *Patu*. Abbreviations in text and figures: A – Epigynal atrium; AME – Anterior median eyes; BI – Bayesian Inference; C – Conductor; C1 – Conductor, anterior projection; C2 – conductor, posterior projection; Cd – Copulatory duct; Ch – Chelicera; ChT – cheliceral tooth; Co – Copulatory opening; Ct – cymbial tooth; Cy – Cymbium; E – Embolus; Em – Embolic membrane; EMD – Epigynal median duct; F – Femur; Fd – Fertilization duct; Lb – lateral branch of the EMD; LE – lateral eyes; Mcl – male leg II mating clasper; ML – Maximum Likelihood; MP – Maximum Parsimony; Pa – Patella; Pc – Paracymbium; PME – Posterior median eyes; S – Spermatheca; Sa – Secretory ampulla; Sc – Epigynal scape; Sd – Spermatic duct; T – Tibia.

## Results

### Phylogenetic analysis

**Figure 1.**
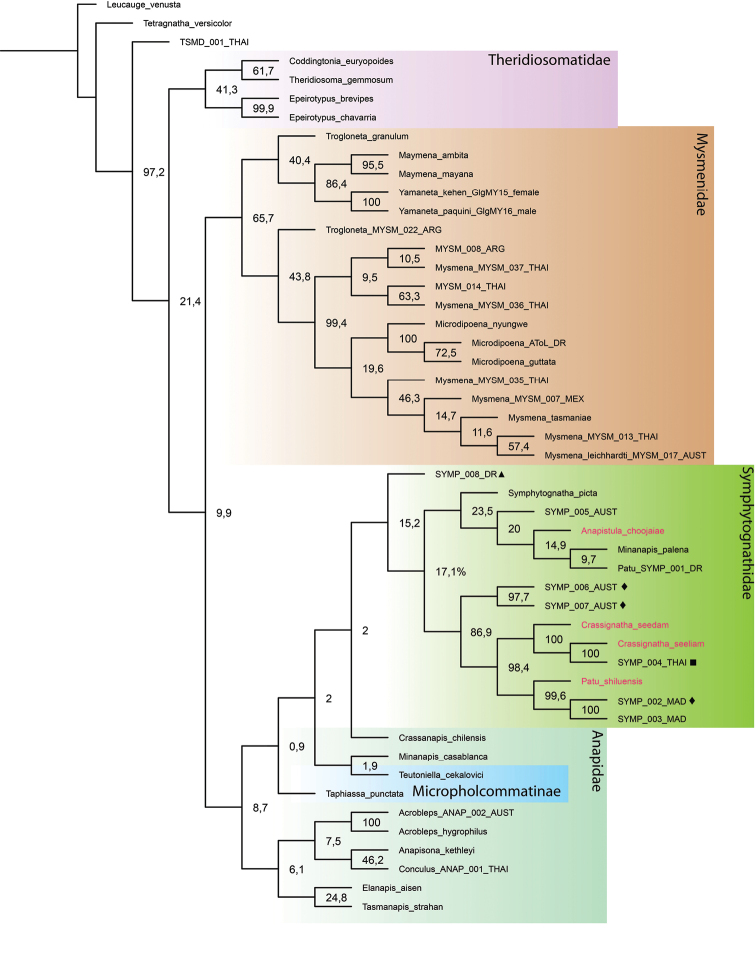
Tree topology obtained by Maximum Parsimony in MEGA-X using a modified version of Lopardo et al., (2011) and Feng et al., (2019) plus the four symphytognathid species from our study (in red). Numbers at nodes indicate bootstrap support. Note the paraphyly of Anapidae and the high support of *Crassignatha* and *Patu* in the Symphytognathidae. Molecular vouchers used for previous “symphytognathoid” studies (Lopardo et al. 2011; Lopardo and Hormiga 2015) identified to genus level by L. Lopardo (*pers. comm.*) as follows: ■ *Crassignatha* (apparently conspecific with *C. seeliam*); ◆*Patu*; and ▲*Symphytognatha*.

**Figure 2.**
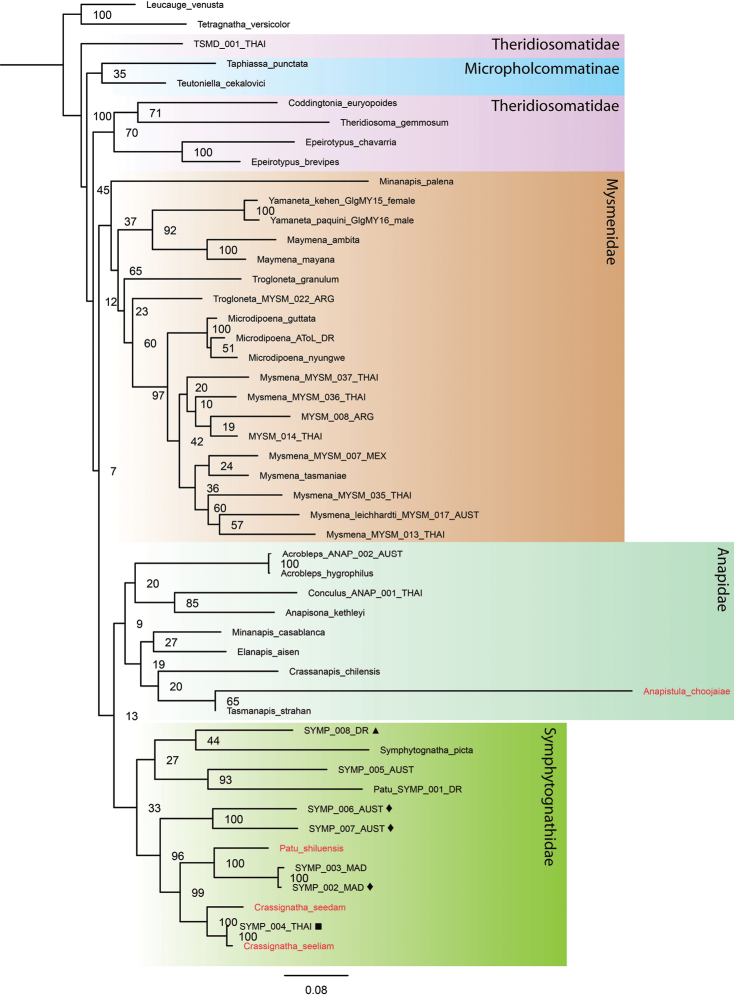
Tree topology obtained by Maximum Likelihood in RAxML using a modified version of Lopardo et al. (2011) and Feng et al. (2019) plus the four symphytognathid species from our study (in red). Numbers at nodes indicate bootstrap support. Note the long branch of *Anapistula* and its position within Anapidae; and the high support of *Crassignatha* and *Patu* in the Symphytognathidae. Molecular vouchers used for previous “symphytognathoid” studies (Lopardo et al. 2011; Lopardo and Hormiga 2015) identified to genus level by L. Lopardo (*pers. comm.*) as follows: ■ *Crassignatha* (apparently conspecific with *C. seeliam*); ◆*Patu*; and ▲*Symphytognatha*.

**Figure 3.**
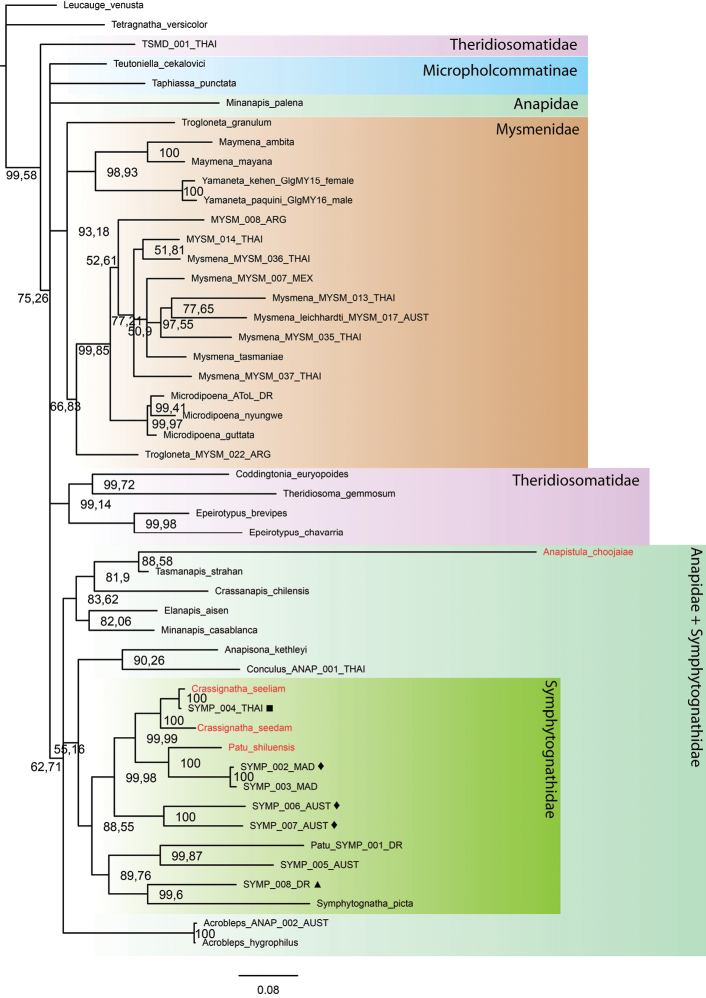
Tree topology obtained by Bayesian Inference in Mr. Bayes using a modified version of Lopardo et al. (2011) and Feng et al. (2019) plus the four symphytognathid species from our study (in red). Numbers at nodes indicate percent posterior probabilities. Note the unresolved relations of the Anapidae and the highly supported monophyly of Symphytognathidae. Molecular vouchers used for previous “symphytognathoid” studies (Lopardo et al. 2011; Lopardo and Hormiga 2015) identified to genus level by L. Lopardo (pers. comm.) as follows: ■ *Crassignatha* (apparently conspecific with *C. seeliam*); ◆*Patu*; and ▲*Symphytognatha*.

Tree topologies inferred by the different phylogenetic analyses performed (Figs 1–3) show some consistencies in several groupings; however, low support values are common, especially in the MP and ML trees. There is an inconsistent and problematic placement of the Symphytognathidae in relation to the Anapidae. All tree analyses recovered Mysmenidae as monophyletic and a sister group of Anapidae + Symphytognathidae. Theridiosomatidae is recovered as monophyletic in the MP and ML analyses with medium to high support (Figs 1, 2); nevertheless, in the BI the position of this family is not resolved (Fig. 3). Similarly, the position of Micropholcommatinae, currently considered part of the Anapidae, is not clear, being found as paraphyletic in the MP, unresolved in the BI, and a poorly supported monophyletic clade in the ML analysis (Figs 1–3). The Anapidae is closely related to the Symphytognathidae in all our trees (with the notable exception of the two micropholcommatines in the ML and BI); however, it appears as a poorly supported monophyletic group in the ML (Fig. 2), and paraphyletic in the MP and BI (Figs 1, 3). The Symphytognathidae appear monophyletic with moderate to high support in all the analyses (Figs 1, 2). In the BI analysis, this family is monophyletic and highly supported but found in an unresolved branch that includes the paraphyleticAnapidae (Fig. 3). The internal relations of the Symphytognathidae are similar in all our trees forming one clade that includes *Symphytognatha picta*, one species (SYMP_008_DR) identified as *Symphytognatha*, one as *Patu* (*Patu*_SYMP_001_DR), and one more (SYMP_005_AUST) that remained unidentified. The other clade recovers the rest of the *Patu* species + *Crassignatha*. Here, two terminals (SYMP_002_MAD and SYMP_003_MAD) are closer to *Patu shiluensis* and related to the three *Crassignatha* representatives; and two other (SYMP_006_AUS and SYMP_007_AUS) are consistently found outside of the *Crassignatha* + *Patu* clade. SYMP-004-THAI consistently clusters with *Crassignatha seeliam* sp. nov., and unpublished morphological observations (Lopardo, pers. comm.) are consistent with the possibility that these are conspecific.

### Micro-CT and 3D modelling

**Figure 4.**
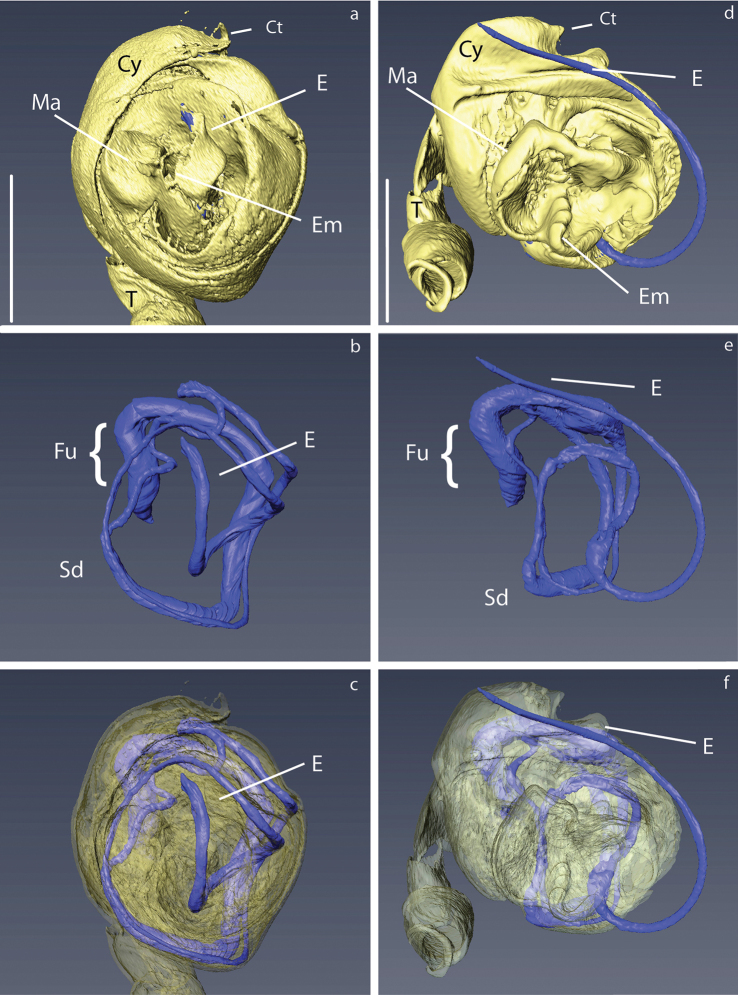
3D reconstruction of the male palp of *Crassignatha* with detail in the spermatic ducts: **a–c***C. seeliam* sp. nov. **d–f***C. danaugirangensis*. Scale bars: 0.1 mm.

**Figure 5.**
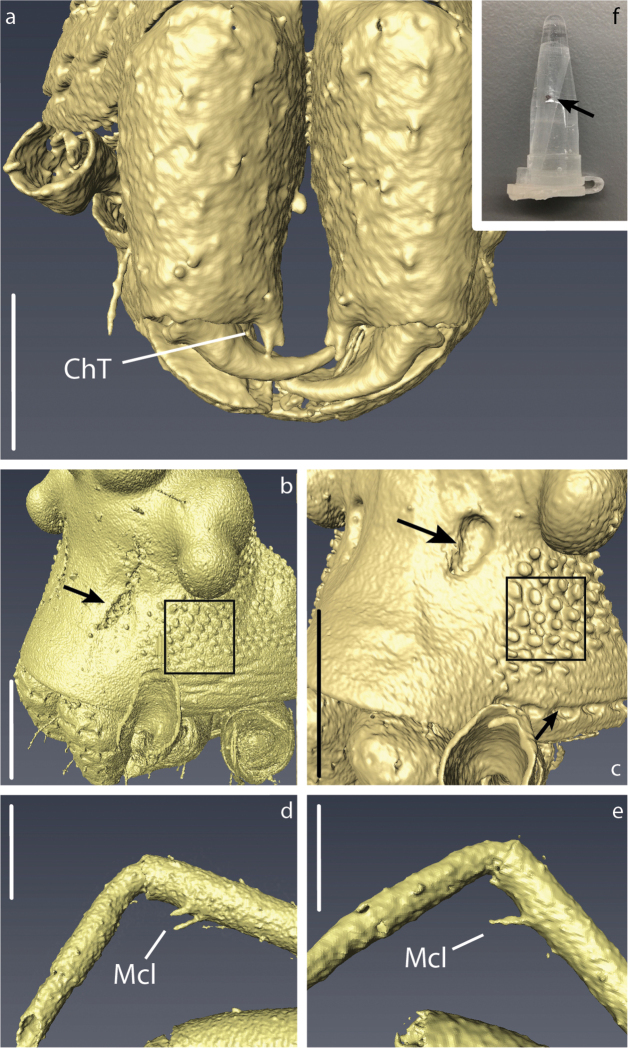
3D reconstruction of some diagnostic characters of *Crassignatha* males: **a, c, e***C. danaugirangensis***b, d***C. seeliam* sp. nov. **a** chelicerae, arrow pointing at the bifurcated tooth **b, c** detail of the carapace; cephalothorax tubercles (in the squares), and pore bearing sulcus (arrows) **d, e** male leg II clasper **f** whole male specimen of *C. danaugirangensis* prepared for micro-CT inside a modified 10 µl pipette tip and a 0.5 ml Eppendorf tube filled with 70% Et-OH. Scale bars: 0.06 mm (**a**); 0.1 mm (**b–e**).

**Figure 6.**
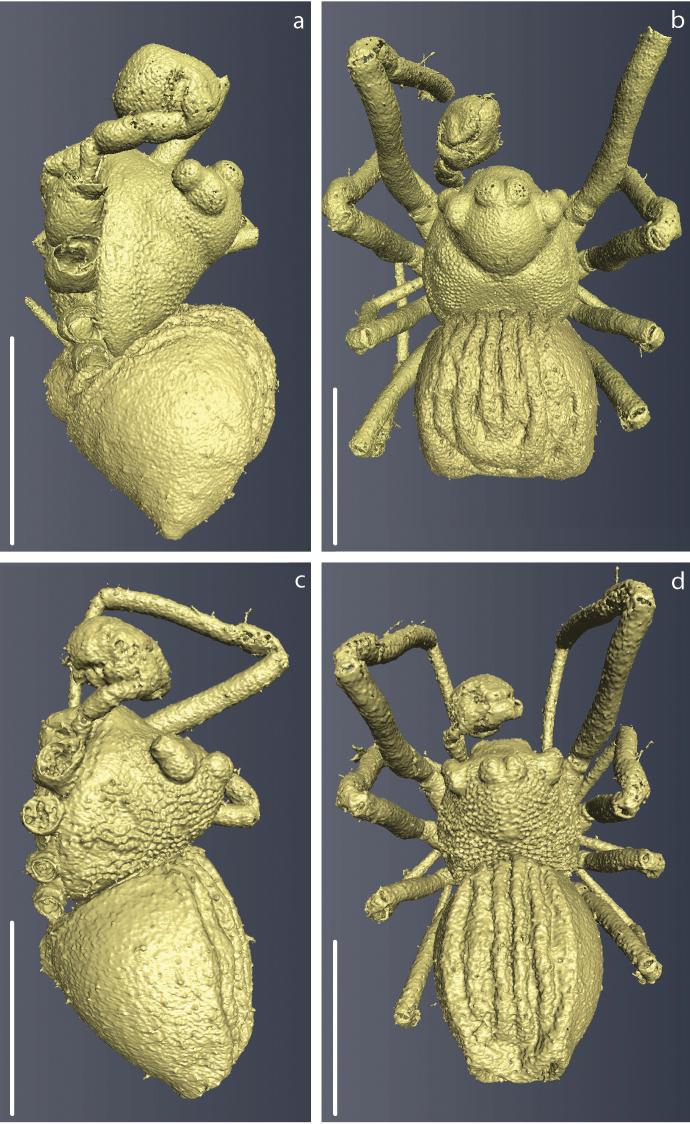
3D reconstruction of the habitus of *Crassignatha* males: **a, b***C. seeliam* sp. nov. **c, d***C. danaugirangensis*. Right pedipalp was dissected previous to the scanning. Scale bars: 0.3 mm.

The micro computed tomography scans allowed us to observe in detail small structures of the surface and internal ducts of the male genitalia (Fig. 4a–f). Structures like the cheliceral teeth (Fig. 5a), cephalothorax tubercles (Fig. 5b, c), and mating clasper on male tibia II (Fig. 5d, e) were also observed. We reconstructed 3D models of the whole body surface of *Crassignatha seeliam* (Fig. 6a, b) and *Crassignata danaugirangensis* (Fig. 6c, d). All of these images were important to examine, interpret and clarify the diagnostic characters of the genus *Crassignatha*. Additional views of the pedipalps, spermatic ducts and habitus can be found in the Suppl. material 2, 3)

### Taxonomy

**Family Symphytognathidae Hickman, 1931**

#### Genus *Anapistula* Gertsch, 1941

*Anapistula* Gertsch, 1941: 2. Type species *Anapistula secreta* Gertsch, 1941.

##### Anapistula choojaiae sp. nov.

Taxon classificationAnimaliaAraneaeSymphytognathidae

58405756-3675-560A-8D43-8BA719E77DAC

http://zoobank.org/916E1BC0-A72E-4B04-9C65-114FC0876E99

Figures 7 , 8 , 9

###### Material examined.

***Holotype***: Thailand • ♂; Chiang Mai, Pha Daeng National Park. Riparian tropical forest; 19°37.768'N, 98°57.257'E. 560 m; July 16–19, 2018; Booppa Petcharad, Jeremy Miller, F. Andres Rivera-Quiroz leg.; Winkler extractor; RMNH.ARA.18442. ***Paratypes***: Thailand • ♀ allotype; same data as holotype • 1♂ 1♀; same data as holotype; RMNH.5106639 • 2♀; Pha Daeng National Park. Bamboo forest; 19°37.668'N, 98°57.131'E. 573 m, same dates and collectors as holotype; RMNH.ARA.18443.

**Figure 7.**
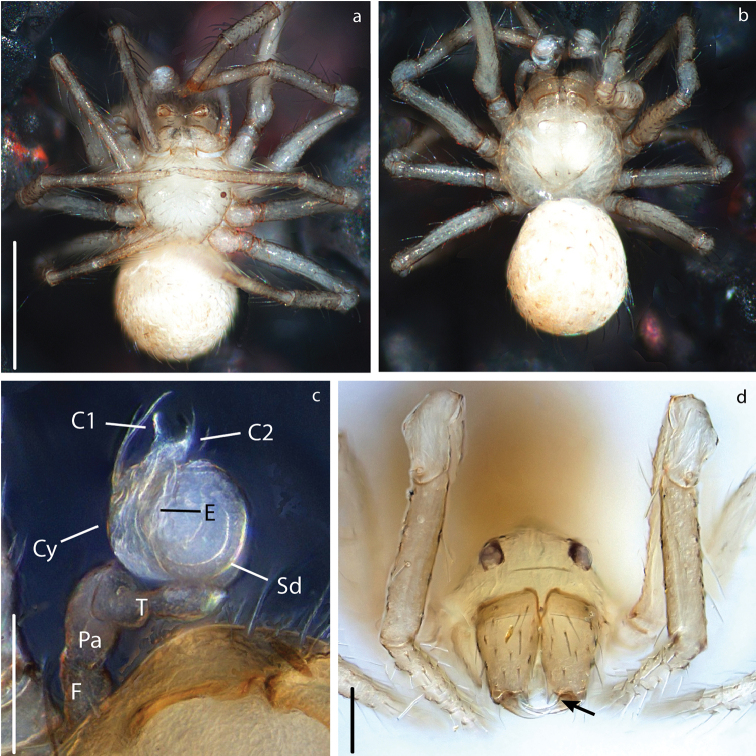
*Anapistula choojaiae* sp. nov. male: Habitus: **a** ventral view **b** dorsal view. Palp: **c** ventral view. Female: Prosoma: **d** anterior view. Scale bars: 0.2 mm (**a, b**); 0.07 mm (**c**); 0.06 mm (**d**). Arrow pointing to the cheliceral teeth.

###### Etymology.

The species epithet is a Latinized matronym of the second authors’ daughter.

###### Diagnosis.

**Figure 8.**
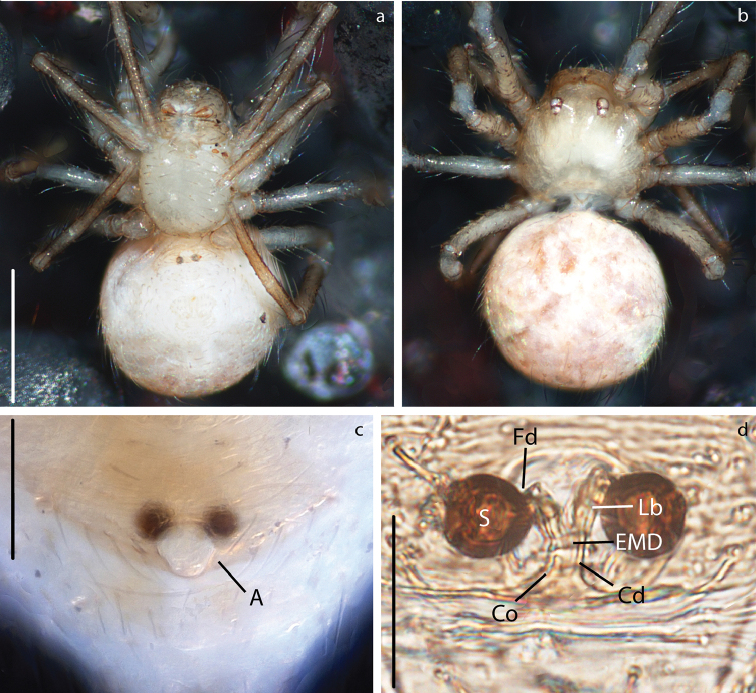
*Anapistula choojaiae* sp. nov. female: Habitus: **a** ventral view **b** dorsal view. Epigynum: **c** ventral view **d** dorsal view, cleared. Scale bars: 0.2 mm (**a, b**); 0.06 mm (**c**); 0.03 mm (**d**).

**Figure 9.**
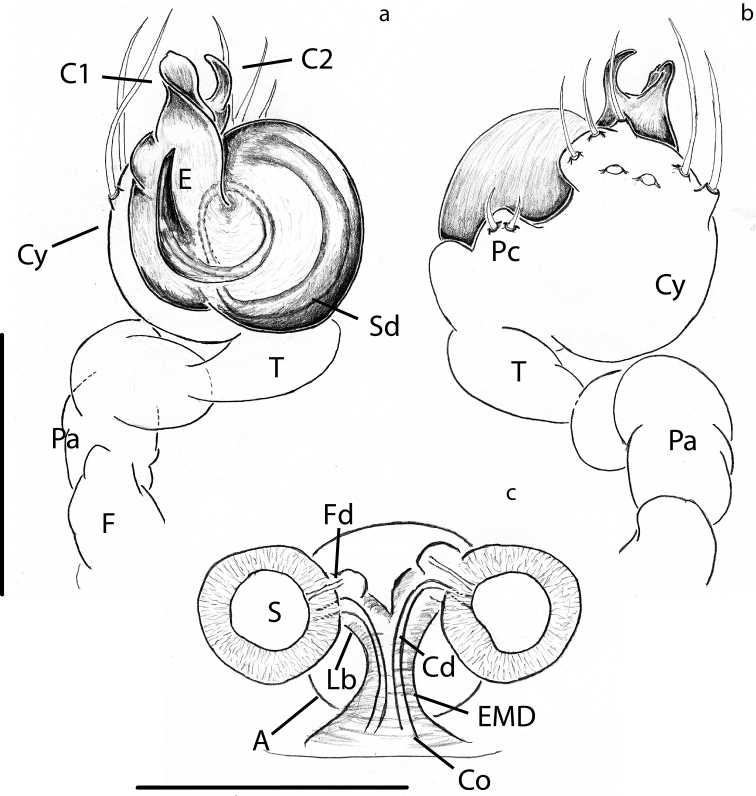
*Anapistula choojaiae* sp. nov., genitalia. Palp: **a** ventral view **b** dorsal view. Epigynum, cleared: **c** dorsal view. Scale bars: 0.07 mm (**a, b**); 0.06 mm (**c**).

Female genitalia in *Anapistula* show little morphological variation between congeneric species making it generally difficult to tell species apart. However, *A. choojaiae* sp. nov. can be distinguished from most *Anapistula* species by the presence of an epigynal atrium; *A. aquytabuera* Rheims & Brescovit, 2003, *A. pocaruguara* and *A. ybyquyra* Rheims & Brescovit, 2003 from Brazil, *A. panensis* Lin, Tao, and Li 2013 and *A. zhengi* Lin, Tao, and Li 2013 from China, and *A. seychellensis* Saaristo, 1996 from the Seychelles also share this character. *A. choojaiae* differs from all of these by the relative size and shape of the atrium, the width of the EMD and the bifurcation of the Lb (compare Figs 8d and 9c to Rheims and Brescovit 2003: figs 16, 18, 21; Lin et al. 2013: figs 3, 4, 8, 9; and Saaristo 1996: fig. 3).

Male pedipalp of *A. choojaiae* similar to *A. panensis* in the overall shape of the palp and in having C1 and C2 roughly the same length, but differs on the width of C1 in respect to C2 and the length of the E in relation to C1 (compare Figs 7c, 9a to Lin et al. 2013: figs 1, 2).

###### Description.

Carapace ovoid, yellowish-white with smooth texture (Figs 7a, b, 8a, b). AME absent (Fig. 7d). Male LE without pigmentation (Figs 7b, 8b). Chelicerae with two promarginal teeth (Fig. 7d). Legs same color as carapace with slightly darker color on distal segments. Abdomen sub-spherical with small sparse sclerotized patches, some bearing long setae (Figs 7b, 8b). Scuta absent in both sexes.

***Male palp***: Weakly sclerotized (Fig. 7c). Semicircular from ventral view (Figs 7c, 9a). With one wide sheet shaped conductor that presents two projections, here called C1 and C2 (Fig. 9a, b). Embolus short and transparent located posteriorly to C; very difficult to see (Figs 7c, 9a).

***Vulva***: Epigynal plate flat, without scape. Atrium semi-circular as wide as inner distance between S (Fig. 8c). Spermathecae spherical, heavily sclerotized in relation to the rest of the body (Fig. 8d). Cd easy to distinguish inside the EMD. LB diverging from the EMD forming a “Y” (Figs 8d, 9c). Fertilization ducts very short and difficult to see, they appear as small bumps on the distal portion of Lb (Fig. 9c).

**Male**: Total length 0.4; carapace 0.2 long, 0.21 wide; clypeus 0.03; Chelicera 0.1 long, 0.06 wide; Leg I: femur 0.26, patella 0.1, tibia 0.17, metatarsus 0.09 tarsus 0.17; leg formula IV-I-II-III; abdomen 0.21 long, 0.21 wide.

**Female**: Total length 0.43, carapace 0.2 long, 0.21 wide; clypeus 0.3; Chelicera 0.1 long, 0.05 wide; Leg I: femur 0.20, patella 0.09, tibia 0.14, metatarsus 0.16, tarsus 0.1; leg formula IV-I-II-III; abdomen 0.24 long, 0.23 wide.

#### Genus *Crassignatha* Wunderlich, 1995

*Crassignatha* Wunderlich, 1995: 547. Type species *Crassignatha haeneli* Wunderlich, 1995.

##### Crassignatha seeliam sp. nov.

Taxon classificationAnimaliaAraneaeSymphytognathidae

6EFF0126-E379-5E8B-8FBC-5DF030F5AF05

http://zoobank.org/DA61A955-A1D4-4B7D-A7A0-89AD024460A3

**Figure 10.**
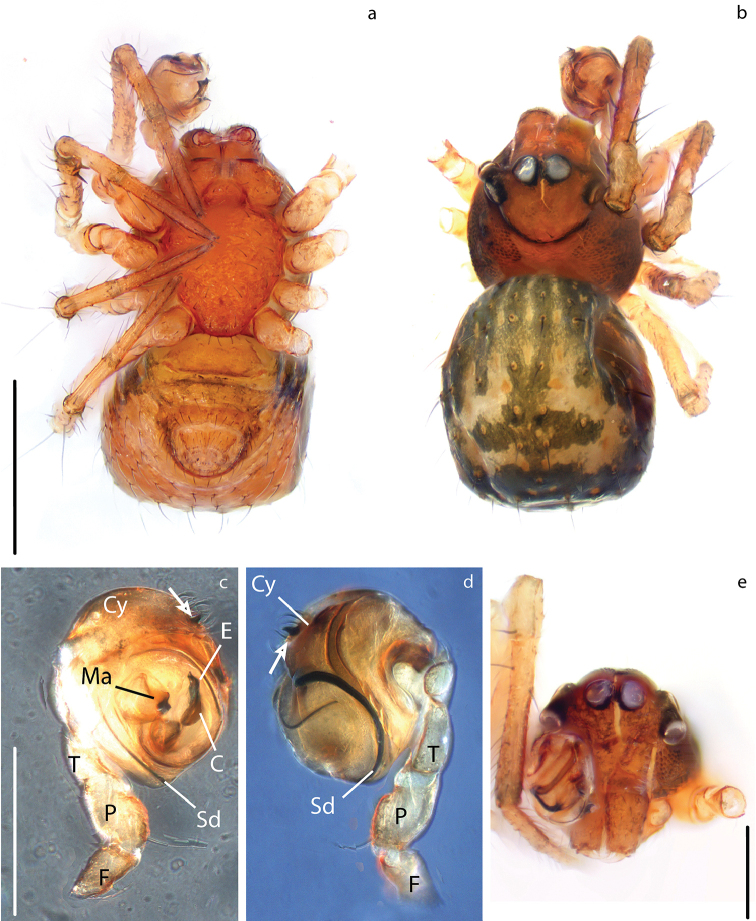
Crassignatha seeliam sp. nov., male: Habitus: **a** ventral view **b** dorsal view. Palp: **c** ventral view **d** retrolateral view. Prosoma: **e** anterior view. Scale bars: 0.3 mm (**a, b**); 0.15 mm (**c–e**). Arrow pointing at the cymbial tooth.

**Figure 11.**
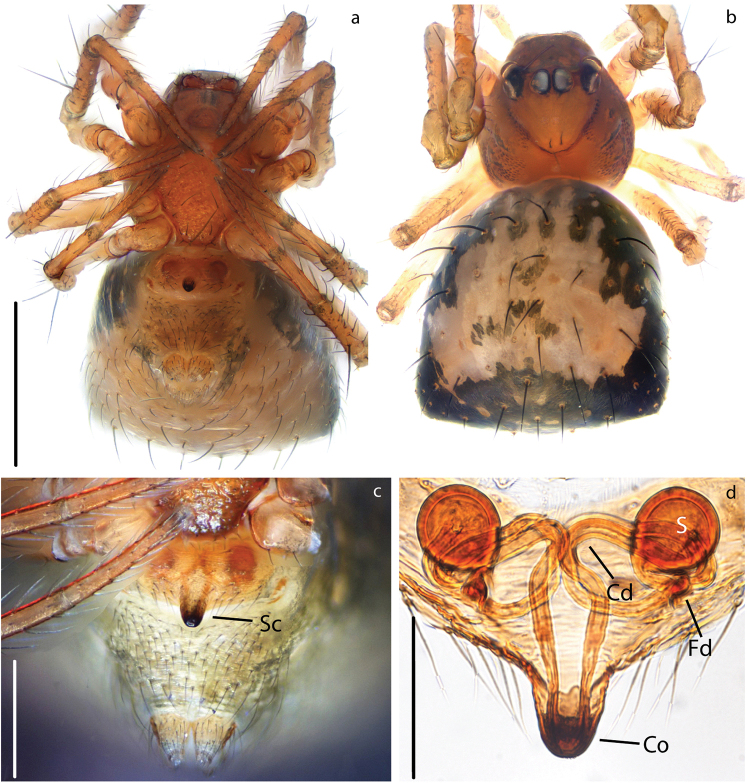
Crassignatha seeliam sp. nov. female: Habitus: **a** ventral view **b** dorsal view. Epigynum: **c** ventral view **d** dorsal view, cleared. Scale bars: 0.4 mm (**a, b**); 0.15 mm (**c**); 0.07 mm (**d**).

**Figure 12.**
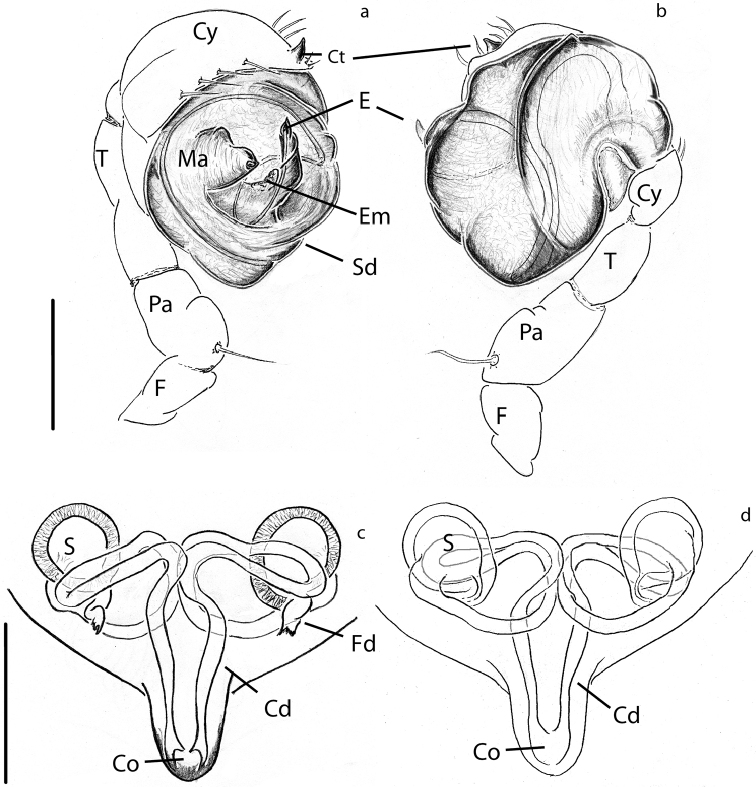
Crassignatha seeliam sp. nov., genitalia. Palp: **a** ventral view **b** dorsal view. Epigynum, cleared: **c** dorsal view **d** ventral view. Scale bars: 0.1 mm (**a, b**); 0.07 mm (**c, d**).

Figures 4a–c , 5b, d , 6a, b , 10 , 11 , 12

###### Material examined.

***Holotype*** : Thailand • ♂: Chiang Mai, Doi Inthanon National Park. Montane evergreen forest; 18°30.454'N, 98°30.584'E. 1605 m; July 21–24, 2018; Booppa Petcharad, Jeremy Miller, F. Andres Rivera-Quiroz leg.; direct hand coll.; RMNH.ARA.18444. ***Paratypes*** : Thailand • ♀ allotype; same data as holotype • 8 ♀; same data as holotype; RMNH.5106641• ♂ and ♀ Chiang Mai, Doi Suthep National Park. Montane evergreen forest with pine; 18°48.502'N, 98°53.528'E. 1409 m; July 24–28, 2018; same collectors as holotype; pitfall traps. RMNH.ARA.18445.

###### Etymology.

 The species epithet is a derivation of the Thai seeliam (square), in reference to the shape of the abdomen in dorsal view. 

###### Diagnosis.

 Distinguished from other Crassignatha species except Crassignatha quadriventris (Lin & Li, 2009) by the semi-squared posterior of the abdomen in dorsal view (Figs 10b, 11b). Female can be separated from C. quadriventris by the coiling of the copulatory ducts in the epigynum (compare Figs 11d and 12c, d to Lin and Li 2009: fig. 10). Male differs on the size of tegular sclerites and the cymbial tooth being short and stout instead of hook-shaped (compare Figs 10c, d and 12a, b to Lin and Li 2009: fig. 8). 

###### Description.

Carapace coloration orange-brown covered by small tubercles (Figs 6a, b, 10a, b, 11a, b). Legs same color, slightly darker on distal portion its segments. Male Tibia II with two spines (mating claspers) (Fig. 5d). Abdomen black with light red patches; squared posteriorly, with sparse sclerotized patches, some bearing long setae (Figs 10b, 11b). Male with posterior scutum wrapping the abdomen. Male palp: slightly less sclerotized than carapace. Semicircular from ventral view (Figs 10c, 12a). Cymbium with distal tooth. Median apophysis as big as Ct (Fig. 12a). Embolus filiform, exposed when palp is expanded (Fig. 12c). Spermatic duct very long and coiling 2× inside the bulb (Fig. 4b, c).

***Vulva***: Epigynum with wide scape directed ventrally, heavily sclerotized at the tip (Fig. 11c). Copulatory opening at the tip of scape (Figs 11d, 12c, d). Spermathecae spherical, slightly more sclerotized than epigynum, separated by ca. 2× their diameter (Fig. 11d). Copulatory ducts very long, coiling over themselves before connecting to S. Fertilization ducts as long as S width, projecting dorsally (figs 11d, 12c). 

**Male**: Total length 0.68; carapace 0.36 long, 0.30 wide; clypeus 0.13; Chelicera 0.1 long, 0.07 wide; Leg I: femur 0.28, patella 0.12, tibia 0.37, metatarsus 0.17, tarsus 0.22; leg formula I-II-IV-III; abdomen 0.42 long, 0.38 wide. 

**Female**: Total length 0.69, carapace 0.44 long, 0.39 wide; clypeus 0.12; Chelicera 0.15 long, 0.1 wide; Leg I: femur 0.42, patella 0.15, tibia 0.53, metatarsus 0.22, tarsus 0.27; leg formula I-II-IV-III abdomen 0.44 long, 0.43 wide. 

##### Crassignatha seedam sp. nov.

Taxon classificationAnimaliaAraneaeSymphytognathidae

DD262AC5-F48F-5447-8C15-BEA6D7C013F2

http://zoobank.org/0562D340-D322-49C4-A029-E95B47110BB5

Figures 13 , 15b, d

###### Material examined.

***Holotype***: Thailand • ♀ Chiang Mai, Doi Suthep National Park. Montane evergreen forest with pine; 18°48.502'N, 98°53.528'E. 1409 m; July 24–28, 2018. Booppa Petcharad, Jeremy Miller, F. Andres Rivera-Quiroz leg.; direct hand coll.; RMNH.5106640. **Male** unknown.

###### Etymology.

 The species epithet is a derivation of the Thai *seedam* (black), in reference to the dark coloration of this species.

###### Diagnosis.

**Figure 13.**
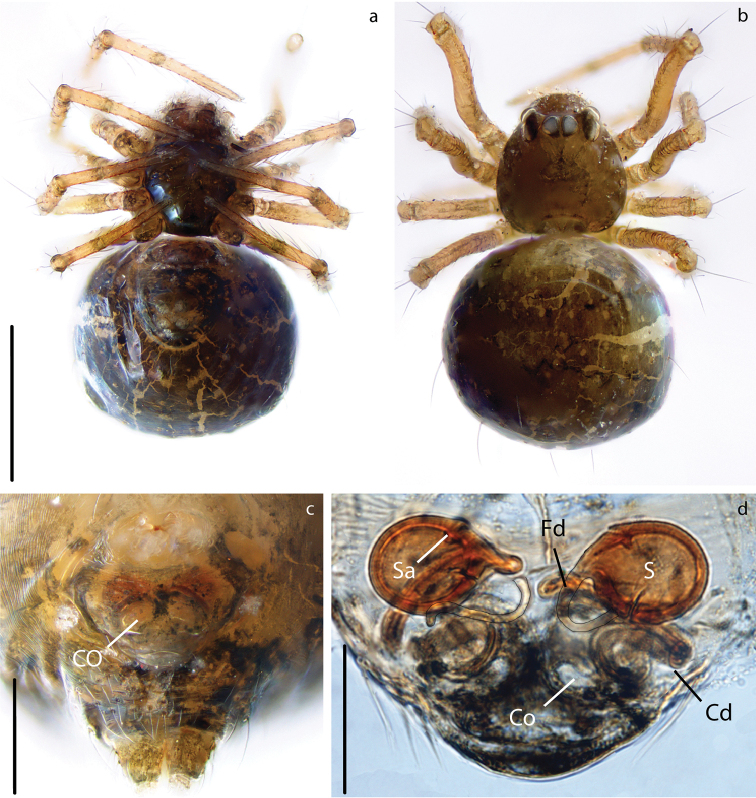
*Crassignatha seedam* sp. nov. female: Habitus: **a** ventral view **b** dorsal view. Epigynum: **c** ventral view **d** dorsal view, cleared. Scale bars: 0.3 mm (**a, b**); 0.1 mm (**c, d**); 0.05 mm (**d**).

*Crassignatha seedam* sp. nov. differs from other *Crassignatha* species by having a nearly round abdomen instead of triangular or squared, and having the epigynum bulging ventro-posteriorly but not forming an scape (compare Figs 13d and 15b, d to Fig. 12c; Lin and Li 2009: fig. 10; and Miller et al. 2009 fig. 76d, h).

###### Description.

Carapace brown with smooth texture (Fig. 13b). Legs light brown, slightly darker on the distal portion its segments. Abdomen sub-spherical, darker than carapace with sparse light patches (Fig. 13a, b).

***Vulva***: Epigynum weakly sclerotized but covered by small dark patches (Fig. 13d), bulging ventrally. Copulatory openings broad but not forming an atrium (Fig. 15b). Spermathecae spherical, much more sclerotized than epigynum, separated by 0.5× their diameter (Fig. 13d). Copulatory ducts long, coiling over themselves before connecting to S. Fertilization ducts as long as S width, connecting very close to Cd and projecting dorsally (Fig. 15b, d).

**Female**: Total length 0.56, carapace 0.28 long, 0.26 wide; clypeus 0.06; Chelicera 0.1 long, 0.07 wide; Leg I: femur 0.3, patella 0.1, tibia 0.22, metatarsus 0.13, tarsus 0.19; leg formula I-II-IV-III; abdomen 0.47 long, 0.41 wide.

##### Crassignatha danaugirangensis

Taxon classificationAnimaliaAraneaeSymphytognathidae

Miller et al., 2014

B478D0CF-D350-546D-A8FB-1FF8749CE83E

Figures 4d–f , 5a, c, e , 6c, d

Crassignatha danaugirangensis Miller et al., 2014: 4, figs 1a–f, 3, 4.

###### New records.

Brunei • 2♂; Temburong, Huala Belalong Field Studies Centre; 4.545°N, 115.157°E, 150 m; September 26 – October 6, 2018; Taxon Expeditions 2018 leg.; Winkler extractor; RMNH.5106643.

#### Genus *Patu* Marples, 1951

*Patu* Marples, 1951: 47. Type species *Patu vitiensis* Marples, 1951.

##### Patu shiluensis

Taxon classificationAnimaliaAraneaeSymphytognathidae

Lin & Li, 2009

D1FCD9D1-AC07-541D-B881-DFE02A1C1BD7

Figures 14 , 15a, c

Patu shiluensis Lin & Li, 2009: 59, figs 11A, B, 12A, B, 13A–D.

###### Collected material.

Thailand • 4♀; Phuket Province, Siray Island. Mixed tropical forest; 7°53.355'N, 98°26.083'E. 132 m; August 02–06, 2018; Booppa Petcharad, Jeremy Miller, F. Andres Rivera-Quiroz leg.; Winkler extractor; RMNH.5106642.

###### Distribution.

Known only from its type locality, Shilu Town, Hainan Province, China and the specimens collected for the present work.

###### Morphological remarks.

**Figure 14.**
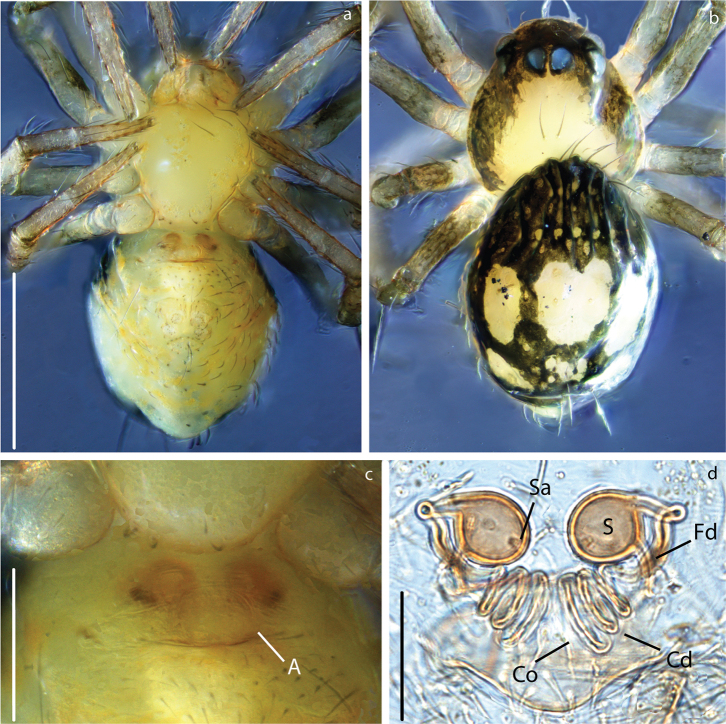
*Patu shiluensis* Lin & Li, 2009 female: Habitus: **a** ventral view **b** dorsal view. Epigynum: **c** ventral view **d** dorsal view, cleared. Scale bars: 0.2 mm (**a, b**); 0.06 mm (**c**); 0.03 mm (**d**).

Carapace pale yellow with black margin, smooth texture (Fig. 14b). Legs black and semi-transparent. Abdomen oval, longer than wide (Fig. 14a, b). Ventrally same color as carapace, dorsally, darker with pale yellow patches.

**Figure 15.**
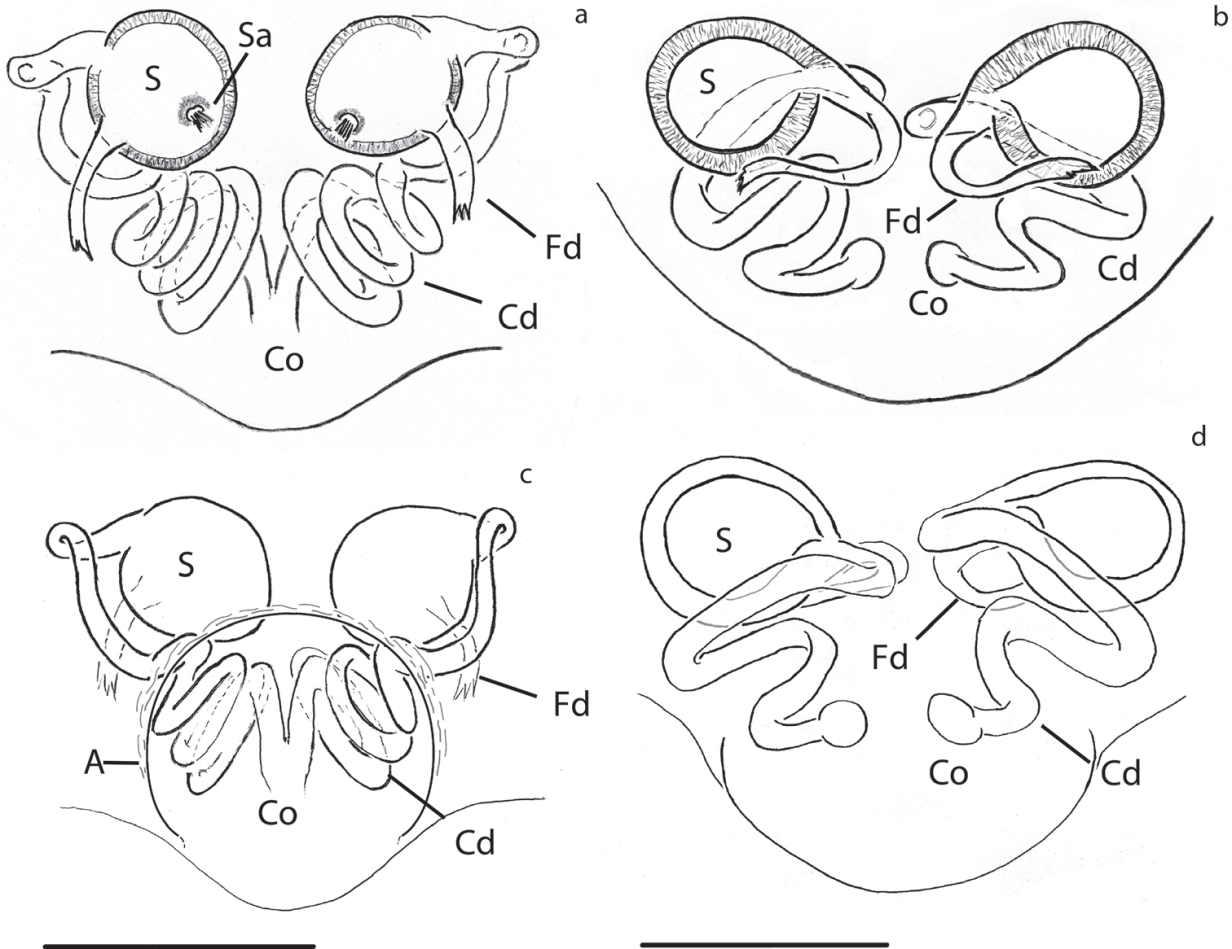
**a, c***Patu shiluensis* Lin & Li, 2009 **b, d***Crassignatha seedam* sp. nov. Epigynum, cleared: **a, b** dorsal view **c, d** ventral view. Scale bars: 0.03 mm (**a, c**); 0.05 mm (**b, d**).

***Vulva***: Epigynum weakly sclerotized, semi-transparent (Fig. 14c). Atrium semi-circular slightly wider than inner distance between S (Figs 14c, 15c). Spermathecae spherical slightly more sclerotized than epigynum, separated by 0.5× their diameter (Fig. 14d). Copulatory ducts spring-like, spiraling 3× over themselves. Fertilization ducts as long as S width, projecting posteriorly (Figs 14d, 15a, c).

**Female**: Total length 0.52, carapace 0.21 long, 0.2 wide; clypeus 0.04; Chelicera 0.07 long, 0.05 wide; Leg I: femur 0.15, patella 0.07, tibia 0.1, metatarsus 0.07, tarsus 0.1; leg formula I-II-IV-III; abdomen 0.34 long, 0.28 wide.

###### Notes.

Small somatic variations can be seen between the specimen we collected in Thailand and the ones previously described from China (compare Fig. 14b to Lin and Li 2009: fig. 11). However, we did not find any objective differences in the female genitalia.

Secretory ampullae (Figs 14d, 15a) were very evident in our specimens; these glandular structures might be homologous to the accessory glands in Lopardo and Hormiga (2015). These structures were found in one anapid (*Tasmanaspis*) and several mysmenids, but scored as absent or unknown for all the symphytognathids.

The authors of this species mentioned it to be close to *Patu silho* Saaristo, 1996 from Seychelles. The possibility of *P. silho* not being a true *Patu* was discussed by its author (Saaristo 1996; 2010) mentioning evident differences on somatic and sexual characters between *P. silho* and other *Patu* species. Nevertheless, the author deemed appropriate to place it in this genus. We also consider this species might be misplaced in *Patu* but would need further and more detailed analysis out of the scope of this work to clarify it (see discussion on *Patu* relationships below).

## Discussion

The monophyly of the Symphytognathidae and its relations to other symphytognathoid spiders have resulted in complications and inconsistencies across different studies. The symphytognathoids were first recognized in a morphological study being formed by four putatively monophyletic families Anapidae, Symphytognathidae, Mysmenidae and Theridiosomatidae (Griswold et al. 1998). The monophyly of this clade has been tested several times using different molecular approaches targeting specific families (Rix et al. 2008; Lopardo et al. 2011; Feng et al. 2019), the Orbiculariae (Fernández et al. 2014), and the whole order Araneae (Wheeler et al. 2017; Kulkarni et al. 2020). However, only a few representatives of the family Symphytognathidae have been used rendering their position and relations largely unexplored. Here, we built on two previous studies that used nine species of Symphytognathidae to test the relations of the Mysmenidae (Feng et al. 2019; Lopardo et al. 2011). Similarly to Feng et al. (2019) low node supports were common in our trees, especially for MP and ML; still, the topologies we observed when including our four species are consistent with the results from these studies. All of our analyses showed a close relationship between the Symphytognathidae and the Anapidae (Figs 1–3). This relationship has also been recovered in previous works (Griswold et al. 1998; Lopardo et al. 2011; Wheeler et al. 2017; Feng et al. 2019). Although tenuous due to the few terminals included, our study fails to recover the monophyly of the Anapidae and the position of micropholcommatids within this family. Our BI tree could not fully resolve the relations between the Anapidae and Symphytognathidae; similar issues have been observed before for the symphytognathoids (Rix et al. 2008; Lopardo et al. 2011; Dimitrov et al. 2012; Fernández et al. 2014; Feng et al. 2019). This has been explained by either the limited set of loci and the relatively low taxon sampling (Feng et al. 2019) or an indication of the polyphyly of the “symphytognathoids” as suggested by three broad scoped phylogenies (Dimitrov et al. 2012; Fernández et al. 2014; Wheeler et al. 2017). Nevertheless, Symphytognathoids were found to be a highly supported monophyletic group in a recent study that used ultraconserved elements (UCE) from 16 species across the four principal symphytognathoid families (Kulkarni et al. 2020)

The internal relations of the Symphytognathidae in our analyses are still unresolved. Most of Lopardo’s identifications (pers. comm.) are found in the *Crassignatha* + *Patu* clade. From these, SYMP_004_THAI (identified to *Crassignatha*; presumably conspecific to *C. seeliam*), and SYMP_002_MAD and SYMP_003_MAD (*Patu*) group together with the other representatives of the genera they were identified to. But the placing of two more, SYMP_006_AUS and SYMP_007_AUS (*Patu*), is more ambiguous being found outside of the *Crassignatha* + *Patu* clade rendering *Patu paraphyletic*. This clade and its internal relations are highly supported in all our trees (Figs 1–3). Other two sequences, SYMP_008_DR (*Symphytognatha*) and Patu_SYMP_001_DR, are consistently grouped in another branch of the Symphytognathidae together with *Symphytognatha picta* and other unidentified symphytognathid (Figs 1–3) suggesting that Patu_SYMP_001_DR might be misidentified. The position of *Anapistula* within the Symphytognathidae is also problematic. *Anapistula choojaiae* has a very long branch that is recovered as a sister to *Tasmanapis strahan* Platnick & Forster, 1989 with moderate to high support in the ML and BI (Figs 2, 3). In these two analyses, this branch is related to other Anapidae having much higher support values in the BI than the ML (Figs 2, 3). Nevertheless, the recent UCE study by Kulkarni et al., (2020) places this genus next to *Patu* in a highly supported but taxonomically limited Symphytognathidae. Solving the internal relations of the families Anapidae and Symphytognathidae, and clarifying their delimitations would need a much more detailed examination with a broader taxonomic sample.

The minute size of the symphytognathid spiders complicates the observation of diagnostic traits. Examination and interpretation of many characters require higher magnifications than those a dissection microscope can give. Therefore, SEM images have been previously used in the taxonomy of this family (Forster and Platnick 1977; Rheims and Brescovit 2003; Miller et al. 2009, among others). Unfortunately, the process for getting SEM images is destructive; therefore, rare specimens or short series are not usually prepared in this way and some characters cannot be properly observed. Here we used micro-CT scanning to overcome this issue and get clear views of important characters without damaging the specimens. 3D reconstruction has been used before to elucidate surfaces and internal structures of spider genitalia (Lipke et al. 2015; Sentenská et al. 2017; Dederichs et al. 2019). Nevertheless, ours are, to the best of our knowledge, the smallest palps that have been processed using this method. This was challenging in itself since we wanted to preserve the samples without critical point drying, a method commonly used in micro-CT scanning (Sentenská et al. 2017; Keklikoglou et al. 2019; Steinhoff et al. 2017, 2020). The tiny size of the palps, less than 0.2 mm wide, did not allow to properly fix the dissected organ and keep it from moving during the scanning process. We attempted to fix the palp in agarose gel inside a 10 µl pipette tip, but the contrast of the resulting scans was too low to allow any observations. This problem was solved by scanning the entire spider (without dissecting the palp) in Et-OH 70% inside a modified 10 µL pipette tip that was in turn inside a 0.5 ml Eppendorf tube (Fig. 5f) in a similar fashion to Lipke et al. (2015), and Sombke et al. (2015). With this approach we were able to reconstruct the long and complicated internal ducts of the male genitalia (Fig. 4b, c, e, f), as well as the surface of the external somatic and genital morphology (Figs 4a, b, 5a–e, 6a–d; Suppl. material 2, 3). Other internal structures of the male palp, probably glands, could be observed but would require more detailed examination out of the scope of the present work to accurately determine their nature; therefore, they are not shown in our 3D models. Images obtained through 3D reconstruction were used to interpret and discuss the diagnostic characters of the genus *Crassignatha* and compare them to other Symphytognathid genera in Table 2.

Forster and Platnick (1977) reviewed the Symphytognathidae and its component genera. Five of the eight currently recognized symphytognathid genera were included: *Anapistula* Gertsch, 1941, *Curimagua* Forster & Platnick, 1977, *Globignatha* Balogh & Loksa, 1968, *Patu* Marples, 1951, and *Symphytognatha* Hickman, 1931. *Crassignatha* Wunderlich, 1995 was described based on a single male specimen from peninsular Malaysia. This genus has been associated with several families (Synaphridae, Anapidae, Mysmenidae, Symphytognathidae; Marusik and Lehtinen 2003; Wunderlich 2004; Miller et al. 2009; Lopardo and Hormiga 2015) and is currently considered a symphytognathid. Two other genera currently cataloged as Symphytognathidae, *Iardinis* Simon, 1899 *Anapogonia* Simon, 1905, are unrecognizable (Levi and Levi 1962; Forster and Platnick 1977; Platnick and Forster 1989; Lopardo and Hormiga 2015). Although spider taxonomy generally relies heavily on genitalia, little in the way of descriptive text or helpful depictions of genitalic characters was offered in Forster and Platnick’s (1977) revision. Table 2 summarizes some important diagnostic characters of the currently accepted symphytognathid genera in an attempt to clarify the taxonomic inconsistencies in this family.

**Table 2.** Overview of diagnostic characters of the currently accepted genera of the Symphytognathidae.

	*Anapistula* Gertsch, 1941	*Anapogonia* Simon, 1905	*Crassignatha* Wunderlich, 1995	*Curimagua* Forster & Platnick, 1977	*Globignatha* Balogh & Loksa, 1968	*Iardinis* Simon, 1899	*Patu* Marples, 1951	*Symphytognatha* Hickman, 1931
**Sexes known**	♀ ♂	♀	♀ ♂	♀ ♂	♀	♂	♀ ♂	♀ ♂
**Species number**	25	1	9	2	2	(2)	18	15
**Nomenclatural status**	Valid	Valid	Valid	Valid	Valid	Nomen dubium*	Valid	Valid
**Female genitalia, internal**	Pair of round spermathecae connected by t-shaped duct	–	Large spermathecae, convoluted duct path (Fig. 12c, d)	Ducts follow nearly straight path posteriorly from round spermathecae	Spermathecae twisted anteriorly	N.A.	Spermathecae variable, sometimes elongate or reniform	Copulatory ducts loop around elongate spermathecae (Hickman 1931: figs 1–6, pl. 1, fig. 2)
**Female genitalia, external**	Transverse rounded lip overhanging furrow	–	Short robust scape (Fig. 11c, d)	Transverse rounded lip overhanging furrow	Transverse rounded lip overhanging furrow	N.A.	Transverse rounded lip overhanging furrow, or a flexible scape (Marples 1951: figs 1d, 2e)	Transverse rounded lip overhanging furrow
**Tarsal claws**	Homogeneous	–	Homogeneous	–	Homogeneous	–	Homogeneous	Multidentate only in anterior legs (Forster and Platnick 1977: figs 6, 7; Hickman 1931: fig. 2; Lin 2019: fig. 3)
**Cheliceral fusion**	Near the base	Absent	Near the base	Near the base	Almost entirely fused with no visible suture line (Forster and Platnick 1977: figs 41, 42)	–	Fused basally to ca. 1/2 their length	Fused for most of their length, with visible suture line
**Cheliceral teeth**	Two (Fig. 7d)	–	Single asymmetrically bifid tooth, or two teeth (Fig. 5a)	Absent	One large, two short (Forster and Platnick 1977: fig. 43)	One (Brignoli 1978: fig. 6)	Usually a single large tooth with 1–3 peaks	Two sinuous teeth (Forster and Platnick 1977: figs 3, 32, 36; Lin 2019: figs 2B, 2C; Lopardo and Hormiga 2015: fig. 122A)
**Male tibia II clasper**	Absent	N.A.	1–4 (Fig. 5d, e)	Absent	N.A.	–	Sometimes 1–2	Absent
**Male abdominal scutum**	Absent except in *A. boneti*	N.A.	Surrounding the posterior part of the abdomen. Usually present, except in *C. haeneli*	Absent	N.A.	–	Absent	Absent
**Pars cephalica**	usually only slightly raised, strongly raised in *A. boneti*	–	Strongly raised	Strongly raised	Strongly raised	Strongly raised	Strongly raised	Strongly raised
**Eye arrangement**	Usually four eyes (Fig. 8b), median eyes present in *A. boneti*	Six eyes in triads	Six eyes in diads (Figs 10b, e, 11b)	Six eyes in triads	Six eyes in diads	Six eyes in triads	Six eyes in diads (Fig. 14b)	Six eyes in diads
**Female palp**	Absent	–	Absent	Vestigial	Absent	N.A.	Absent	Absent
**Carapace texture**	Mostly smooth	–	Generally covered with tubercles (Fig. 5b, c)	Mostly smooth	Mostly smooth	–	Mostly smooth	Mostly smooth
**Abdomen shape**	Subspherical	–	Subspherical, sometimes with postero-lateral lobes (Fig. 6)	Subspherical	Subspherical	–	Subspherical, sometimes with lobes	Subspherical
**Cymbium**	With strong setae but without teeth or denticles	N.A.	With cymbial tooth (Fig. 4b, d)	With small bumps or denticles (Forster and Platnick 1977: fig. 66)	N.A.	–	–	–
**Spermatic duct**	Coiling 1.5× over itself (Fig. 9a)	N.A.	Long, coiling several times around itself (Fig. 4b, e)	–	N.A.	Coiling 1.5× over itself (Brignoli 1978: fig. 7; Lopardo and Hormiga 2015: fig 135a)	–	–
**Embolus**	Short less than 0.5× the diameter of the bulb (Figs 7c, 9a)	N.A.	Variable, short (Fig. 4c) or long, ca. the diameter of the palp (Fig. 4f)	Short, ca. 0.5× the diameter of the bulb (Forster and Platnick 1977: figs 67, 68)	N.A.	long, 0,5–1,5 the diameter of the bulb (Brignoli 1978: fig. 7; Brignoli 1980: figs 1, 2)	Long, ca. 1×the diameter of the bulb (Marples 1951: fig. 1e, f; Marples 1955: fig. 19)	Short, ca. 0.5× the diameter of the bulb (Forster and Platnick 1977: figs 8, 9)
**Relevant literature**	(Harvey 1998; Dupérré and Tapia 2017; Forster and Platnick 1977; Rheims and Brescovit 2003; Rubio and González 2010)	(Simon 1905; Platnick and Forster 1989)	(Wunderlich 2004; Miller et al. 2009; Lopardo and Hormiga 2015)	(Forster and Platnick 1977)	(Forster and Platnick 1977)	(Brignoli 1980; Forster and Platnick 1977; Gertsch 1960; Levi and Levi 1962; Lopardo and Hormiga 2015)	(Marples 1951, 1955; Forster 1959; Forster and Platnick 1977; Saaristo 1996)	(Hickman 1931; Forster and Platnick 1977; Lopardo and Hormiga 2015; Lin 2019)

Number of species is based on the WSC (2020). *Type species *Iardinis weyersi* Simon, 1899 is considered a *nomen dubium*; two species placed in this genus by Brignoli (1978, 1980) remain cataloged here (WSC 2020).

Other than their small size, the characteristic that is perhaps most strongly associated with the Symphytognathidae was the fusion of the chelicerae (Forster and Platnick 1977). But the degree of fusion is variable across the family and is particularly problematic in the genus *Patu*. The two species originally placed in *Patu* were reported as having the chelicerae fused for approximately half their length, but the degree of fusion was apparently less extensive in the genotype *Patu vitiensis* than in *Patu samoensis*, the other species described (Marples 1951). Subsequent authors have generally characterized *Patu* as having the chelicerae fused only at the base (Forster and Platnick 1977). Curiously, Forster (1959) made no mention of cheliceral fusion in *Patu*, but he did report basal fusion of the chelicerae in two genera (*Pseudanapis* and *Textricella*) that were subsequently transferred to Anapidae. So, assessing the presence or absence of basal cheliceral fusion is not always straight forward in practice. Some (but not all) *Patu* species known from males have a number of ventral distal macrosetae on tibia II, a characteristic scored as present in Lopardo’s *Patu* specimens SYMP_002_MAD and SYMP_006_AUS and absent in Patu_SYMP_001_DR and *Symphytognatha picta* (Lopardo and Hormiga 2015); this leg II clasper is otherwise found only in *Crassignatha*.

Genotype *Crassignatha haeneli* Wunderlich, 1995 features a textured carapace and a distinctive ventral spur on tibial II (Fig. 5d, e; Wunderlich 1995: figs 14, 15, 17). The chelicerae are not conspicuously fused and are armed with a single bifid tooth (Fig. 5a); a character also scored for three species (SYMP_002_MAD, SYMP_006_AUS and SYMP_007_AUS, later on identified as *Patu*) used in Lopardo and Hormiga (2015). Miller et al. (2009, 2014) placed several additional species in *Crassignatha*, including the first descriptions of females. In all of Miller’s species where males are known, they possess a unique abdominal scutum surrounding the abdomen laterally and posteriorly. In most *Crassignatha* species, the female genitalia consists of a pair of robust round spermathecae separated by approximately their diameter, copulatory ducts that loop and switchback along their path, and a short, robust scape (Miller et al. 2009: figs 76, 79, 89A–D); only *C. longtou* and *C. seedam* sp. nov. have a transverse bulge and not a scape (Miller et al. 2009: figs 89E, F, 91F).

Wunderlich (1995) stated that *Crassignatha haeneli* lacked an abdominal scutum, and among the Symphytognathidae, only *Anapistula boneti* and Miller’s *Crassignatha* species have a scutum (but see *Patu spinathoraxi*, below). A dissection of *Crassignatha* chelicerae indicated that they were indeed fused at the base (Miller et al. 2009: fig. 78A). It is however worth noting that the 3D scan of *Crassignatha* presented here do not appear to indicate cheliceral fusion (Fig. 5a). It was also determined that most of these *Crassignatha* species have an asymmetrical split in the cheliceral tooth with a small peak on the mesal side of the tooth; only *C. longtou* has two subequal teeth. *Crassignatha* species known from the male all have a group of 1–3 strong ventral setae on male tibia II (Miller et al. 2015: figs 74E, 77D, 80E, 83E; Miller et al. 2009: fig. 1F). One species had the abdomen modified with a pair of posteriolateral lobes (Miller et al. 2009: figs 86D–F), not as conspicuous in other species (Fig. 6b, d), or generally round or oblong.

### Modern symphytognathid taxonomy in Asia

2009 was a big year for little spiders in Asia. Four papers described a total of 18 symphytognathid species from China, Japan, and Vietnam (Lin et al. 2009; Lin and Li 2009; Miller et al. 2009; Shinkai 2009). These were distributed across the genera *Anapistula*, *Crassignatha*, and *Patu*. Lin and Li (2009) described five new *Patu* species from China. Again, fusion of the chelicerae only near the base was declared as a characteristic of *Patu*. Chelicerae of all species were illustrated as fused, but no details were provided in the text. Of these five species, three show characters that match the diagnostic characters of *Crassignatha* instead of *Patu*:

*Patu bicorniventris* Lin & Li, 2009, known from the female only, has an asymmetrically bifid cheliceral tooth (Lin and Li 2009: figs 2C, 2D) resembling those typical of *Crassignatha* (Miller et al. 2009: fig. 78A). It also has modifications to the abdomen consisting of two posteriolateral lobes and a straight posterior margin, resembling *Crassignatha ertou* (Miller et al., 2009 figs 86D-86F). The female genitalia of *Patu bicorniventris* resembles most *Crassignatha* females described in Miller et al. (2009), featuring conspicuous spermathecae with convoluted copulatory ducts leading to a knob-like median scape.

*Patu quadriventris* Lin & Li, 2009 shares with *P. bicorniventris* an abdomen that is truncated posteriorly, but lacks the posteriolateral lobes. The female genitalia is consistent with *Crassignatha*. The cymbium of the male pedipalp has a distal apophysis (CS in Lin and Li 2009: fig. 9C) that strongly resembles the Ct in *Crassignatha* (Figs 9a, 13a, d; Miller et al. 2009: figs 75, 77B, 81, 82B, 84, 87, 88).

*Patu spinathoraxi* Lin & Li, 2009 has distinctive spikey tubercles covering the carapace. It closely resembles (but is not conspecific with) *Crassignatha longtou* Miller, Griswold & Yin, 2009, which was described from the female only. The female genitalia of both species are similar, featuring round spermathecae with ducts that run ectally before turning back toward the middle and terminate in a pair of conspicuous posterior openings; they contrast with *Crassignatha* in that they lack a robust scape. The male has a medially split abdominal scutum, a single ventral macroseta on tibia II, and a distal apophysis of the cymbium similar to those found in *Crassignatha* (CS in Lin and Li 2009: fig. 16C). These two species are clearly congeneric; whether they are best placed together in *Crassignatha*, or in their own new genus, is debatable.

### Current status and proposed changes

Of the eight valid symphytognathid genera, *Anapistula*, *Curimagua*, *Globignatha*, *Symphytognatha*, and *Crassignatha* seem morphologically coherent and recognizable; *Anapogonia* and *Iardinis* are currently unrecognizable; *Patu* remains problematic. However, some species currently placed in *Patu* show clear affinities with *Crassignatha*. We propose the following taxonomic changes: *Crassignatha bicorniventris* (Lin & Li, 2009) comb. nov., *Crassignatha quadriventris* (Lin & Li, 2009) comb. nov., and *Crassignatha spinathoraxi* (Lin & Li, 2009) comb. nov.

## Supplementary Material

XML Treatment for Anapistula choojaiae

XML Treatment for Crassignatha seeliam

XML Treatment for Crassignatha seedam

XML Treatment for Crassignatha danaugirangensis

XML Treatment for Patu shiluensis

## Supplementary materials

Supplementary material 1 List of primers used in our study Data type molecular data Explanation note List of primers used in our study, alignment of DNA sequence data used in phylogenetic analyses in nexus format, and Trace plot and histograms for both runs of the BI analysis observed in Tracer 1.7.1. https://binary.pensoft.net/file/501624This dataset is made available under the Open Database License (http://opendatacommons.org/licenses/odbl/1.0/). The Open Database License (ODbL) is a license agreement intended to allow users to freely share, modify, and use this Dataset while maintaining this same freedom for others, provided that the original source and author(s) are credited.F. Andres Rivera-Quiroz, Booppa Petcharad, Jeremy A. Miller

Supplementary material 2 3D reconstructions *Crassignatha seeliam* sp. nov. male pedipalp and habitus
Data type multimedia https://binary.pensoft.net/file/501625This dataset is made available under the Open Database License (http://opendatacommons.org/licenses/odbl/1.0/). The Open Database License (ODbL) is a license agreement intended to allow users to freely share, modify, and use this Dataset while maintaining this same freedom for others, provided that the original source and author(s) are credited.F. Andres Rivera-Quiroz, Booppa Petcharad, Jeremy A. Miller

Supplementary material 3 3D reconstructions *Crassignatha danaugirangensis* male pedipalp and habitus
Data type multimedia https://binary.pensoft.net/file/501626This dataset is made available under the Open Database License (http://opendatacommons.org/licenses/odbl/1.0/). The Open Database License (ODbL) is a license agreement intended to allow users to freely share, modify, and use this Dataset while maintaining this same freedom for others, provided that the original source and author(s) are credited.F. Andres Rivera-Quiroz, Booppa Petcharad, Jeremy A. Miller

## References

[B1] Alvarez-Padilla F Hormiga G (2007) A protocol for digesting internal soft tissues and mounting spiders for scanning electron microscopy. Journal of Arachnology 35(3): 538–542. 10.1636/Sh06-55.1

[B2] Brignoli PM (1978) Spinnen aus Nepal, IV. Drei neue Symphytognathidae (Arachnida: Araneae). Senckenbergiana Biologica 59: 247–252.

[B3] Brignoli PM (1980) On few Mysmenidae from the Oriental and Australian regions (Araneae). Revue Suisse De Zoologie 87: 727–738. 10.5962/bhl.part.85542

[B4] Coddington JA (1983) A temporary slide-mount allowing precise manipulation of small structures. Verhandlungen des Naturwissenschaftlichen Vereins in Hamburg 26: 291–292.

[B5] Darriba D Taboada GL Doallo R Posada D (2012) JModelTest 2: More models, new heuristics and parallel computing. Nature Methods 9: 772–772. 10.1038/nmeth.2109 PMC4594756 22847109

[B6] Dederichs TM Müller CHG Sentenská L Lipke E Uhl G Michalik P (2019) The innervation of the male copulatory organ of spiders (Araneae) – A comparative analysis. Frontiers in Zoology 16(39): 1–14. 10.1186/s12983-019-0337-6 31666802 PMC6813115

[B7] Dimitrov D Lopardo L Giribet G Arnedo MA Álvarez-Padilla F Hormiga G (2012) Tangled in a sparse spider web: Single origin of orb weavers and their spinning work unravelled by denser taxonomic sampling. Proceedings of the Royal Society B – Biological Sciences 279(1732): 1341–1350. 10.1098/rspb.2011.2011 PMC3282380 22048955

[B8] Dupérré N Tapia E (2017) On some minuscule spiders (Araneae: Theridiosomatidae, Symphytognathidae) from the Chocó region of Ecuador with the description of ten new species. Zootaxa 4341(3): 375–399. 10.11646/zootaxa.4341.3.3 29245661

[B9] Feng C Miller JA Lin Y Shu Y (2019) Further study of two chinese cave spiders (Araneae, Mysmenidae), with description of a new genus. ZooKeys 870: 77–100. 10.3897/zookeys.870.35971 31423079 PMC6694075

[B10] Fernández R Hormiga G Giribet G (2014) Phylogenomic analysis of spiders reveals non-monophyly of orb weavers. Current Biology 24(15): 1772–1777. 10.1016/j.cub.2014.06.035 25042584

[B11] Forster RR (1959) The spiders of the family Symphytognathidae. Transactions and Proceedings of the Royal Society of New Zealand 86: 263–329.

[B12] Forster RR Platnick NI (1977) A review of the spider family Symphytognathidae (Arachnida, Araneae). American museum novitates 2619: 1–29.

[B13] Gertsch WJ (1960) Descriptions of American spiders of the family Symphytognathidae. American Museum Novitates 1981: 1–40.

[B14] Griswold CE Coddington JA Hormiga G Scharff N (1998) Phylogeny of the orb-web building spiders (Araneae, Orbiculariae: Deinopoidea, Araneoidea). Zoological Journal of the Linnean Society 123(1): 1–99. 10.1111/j.1096-3642.1998.tb01290.x

[B15] Harvey MS (1998) A review of the Australasian species of *Anapistula* Gertsch (Araneae: Symphytognathidae). Records of the Western Australian Museum 19: 111–120.

[B16] Hickman VV (1931) A new family of spiders. Proceedings of the Zoological Society of London 101(4): 1321–1328. 10.1111/j.1096-3642.1931.tb01063.x

[B17] Hormiga G Griswold CE (2014) Systematics, Phylogeny, and Evolution of Orb-Weaving Spiders. Annual Review of Entomology 59: 487–512. 10.1146/annurev-ento-011613-162046 24160416

[B18] Keklikoglou K Faulwetter S Chatzinikolaou E Wils P Brecko J Kvaček J Metscher B Arvanitidis C (2019) Micro-computed tomography for natural history specimens: a handbook of best practice protocols. European Journal of Taxonomy 522: 1–55. 10.5852/ejt.2019.522

[B19] Kulkarni S Wood H Lloyd M Hormiga G (2020) Spider-specific probe set for ultraconserved elements offers new perspectives on the evolutionary history of spiders (Arachnida, Araneae). Molecular Ecology Resources 20: 185–203. 10.1111/1755-0998.13099 31599100

[B20] Kumar S Stecher G Li M Knyaz C Tamura K (2018) MEGA X: Molecular evolutionary genetics analysis across computing platforms. Molecular Biology and Evolution 35(6): 1547–1549. 10.1093/molbev/msy096 29722887 PMC5967553

[B21] Levi HW Levi LR (1962) The genera of the spider family Theridiidae. Bulletin of the Museum of Comparative Zoology 127: 1–71.

[B22] Lin Y (2019) First report of the spider genus *Symphytognatha* from Asia (Araneae, Symphytognathidae). Zootaxa 4638(2): 291–295. 10.11646/zootaxa.4638.2.8 31712480

[B23] Lin Y Li S (2009) First described *Patu* spiders (Araneae, Symphytognathidae) from Asia. Zootaxa 2154: 47–68.

[B24] Lin Y Pham DS Li S (2009) Six new spiders from caves of Northern Vietnam (Araneae: Tetrablemmidae: Ochyroceratidae: Telemidae: Symphytognathidae). Raffles Bulletin of Zoology 57: 323–342. 10.11646/zootaxa.2154.1.3

[B25] Lin Y Tao Y Li S (2013) Two new species of the genus *Anapistula* (Araneae, Symphytognathidae) from Southern China. Acta Zootaxonomica Sinica 38(1): 53–58.

[B26] Lipke E Hammel JU Michalik P (2015) First evidence of neurons in the male copulatory organ of a spider (Arachnida, Araneae). Biology Letters 11(7): e20150465. 10.1098/rsbl.2015.0465 PMC4528456 26156131

[B27] Lopardo L Hormiga G (2015) Out of the twilight zone: Phylogeny and evolutionary morphology of the orb-weaving spider family Mysmenidae, with a focus on spinneret spigot morphology in symphytognathoids (Araneae, Araneoidea). Zoological Journal of the Linnean Society 173(3): 527–786. 10.1111/zoj.12199

[B28] Lopardo L Giribet G Hormiga G (2011) Morphology to the rescue: Molecular data and the signal of morphological characters in combined phylogenetic analyses-a case study from mysmenid spiders (Araneae, Mysmenidae), with comments on the evolution of web architecture. Cladistics 27(3): 278–330. 10.1111/j.1096-0031.2010.00332.x 34875780

[B29] Mammola S Michalik P Hebets EA Isaia M (2017) Record breaking achievements by spiders and the scientists who study them. PeerJ 5: e3972. 10.7717/peerj.3972 PMC5668680 29104823

[B30] Marples BJ (1951) Pacific Symphytognathid Spiders. Pacific Science 5: 47–51.

[B31] Marples BJ (1955) Spiders from Wesern Samoa. Journal of the Linnean Society of London, Zoology 42: 453–504. 10.1111/j.1096-3642.1955.tb02217.x

[B32] Marusik YM Lehtinen PT (2003) Synaphridae Wunderlich, 1986 (Aranei: Araneoidea), a new family status, with a description of a new species from Turkmenistan. Arthropoda Selecta 11: 143–152.

[B33] Miller JA Griswold CE Yin C (2009) The symphytognathoid spiders of the Gaoligongshan, Yunnan, China (Araneae: Araneoidea): Systematics and diversity of micro-orbweavers. ZooKeys 11: 9–195. 10.3897/zookeys.11.160

[B34] Miller JA Griswold CE Haddad CR (2010a) Taxonomic revision of the spider family Penestomidae (Araneae, Entelegynae). Zootaxa (2534): 1–36. 10.11646/zootaxa.2534.1.1

[B35] Miller JA Schilthuizen M Burmester J van der Graaf L Merckx V Jocqué M Kessler P Fayle T Breeschoten T Broeren R Bouman R Chua W-J Feijen F Fermont T Groen K Groen M Kil N de Laat H Moerland M Moncoquet C Panjang E Philip A Roca-Eriksen R Rooduijn B van Santen M Swakman V Evans M Evans L Love K Joscelyne S Tober A Wilson H Ambu L Goossens B (2014) Dispatch from the field: ecology of ground-web-building spiders with description of a new species (Araneae, Symphytognathidae). Biodiversity Data Journal 2: e1076. 10.3897/BDJ.2.e1076 PMC4031436 24891829

[B36] Miller MA Pfeiffer W Schwartz T (2010b) Creating the CIPRES Science Gateway for inference of large phylogenetic trees. In: 2010 Gateway Computing Environments Workshop, GCE 2010, 1–8. 10.1109/GCE.2010.5676129

[B37] Platnick NI Forster RR (1989) A revision of the temperate South American and Australasian spiders of the family *Anapidae* (Araneae, Araneoidea). Bulletin of the American Museum of Natural History 190: 1–139.

[B38] Rambaut A Drummond AJ Xie D Baele G Suchard MA (2018) Posterior summarization in Bayesian phylogenetics using Tracer 1.7. Systematic Biology 67(5): 901–904. 10.1093/sysbio/syy032 29718447 PMC6101584

[B39] Rheims C Brescovit AD (2003) Description of six new species of *Anapistula* Gertsch (Araneae, Symphytognathidae) from Brazil. Bulletin of the British Arachnological Society 12(7): 324–330.

[B40] Rix MG Harvey MS Roberts JD (2008) Molecular phylogenetics of the spider family Micropholcommatidae (Arachnida: Araneae) using nuclear rRNA genes (18S and 28S). Molecular Phylogenetics and Evolution 46(3): 1031–1048. 10.1016/j.ympev.2007.11.001 18162409

[B41] Ronquist F Huelsenbeck JP (2003) MrBayes 3: Bayesian phylogenetic inference under mixed models. Bioinformatics 19(12): 1572–1574. 10.1093/bioinformatics/btg180 12912839

[B42] Rubio GD González A (2010) The first Symphytognathidae (Arachnida: Araneae) from Argentina, with the description of a new species of *Anapistula* from the Yungas Mountain rainforest. Revista Chilena de Historia Natural 83(2): 243–247. 10.4067/S0716-078X2010000200005

[B43] Saaristo MI (1996) Symphytognathidae (Arachnida, Araneae), a new spider family for the granitic islands of Seychelles. Phelsuma 4: 53–56.

[B44] Saaristo MI (2010) Araneae. In: Gerlach J, Marusik Y (Eds) Arachnida and Myriapoda of the Seychelles islands. Press Manchester, UK, 306 pp.

[B45] Sentenská L Müller CHG Pekár S Uhl G (2017) Neurons and a sensory organ in the pedipalps of male spiders reveal that it is not a numb structure. Scientific Reports 7: e12209. 10.1038/s41598-017-12555-5 PMC5610179 28939892

[B46] Simon E (1905) Arachnides de Java, recueillis par le Prof. K. Kraepelin en 1904. Mitteilungen aus dem Naturhistorischen Museum in Hamburg 22: 49–73.

[B47] Sombke A Lipke E Michalik P Uhl G Harzsch S (2015) Potential and limitations of X-Ray micro-computed tomography in arthropod neuroanatomy: A methodological and comparative survey. Journal of Comparative Neurology 523(8): 1281–1295. 10.1002/cne.23741 PMC4409823 25728683

[B48] Stamatakis A (2014) RAxML version 8: A tool for phylogenetic analysis and post-analysis of large phylogenies. Bioinformatics 30(9): 1312–1313. 10.1093/bioinformatics/btu033 24451623 PMC3998144

[B49] Steinhoff POM Uhl G Harzsch S Sombke A (2020) Visual pathways in the brain of the jumping spider *Marpissa muscosa*. Journal of Comparative Neurology 528(11): 1883–1902. 10.1002/cne.24861 31960432

[B50] Steinhoff POM Sombke A Liedtke J Schneider JM Harzsch S Uhl G (2017) The synganglion of the jumping spider *Marpissa muscosa* (Arachnida: Salticidae): Insights from histology, immunohistochemistry and microCT analysis. Arthropod Structure and Development 46(2): 156–170. 10.1016/j.asd.2016.11.003 27845202

[B51] Tong Y Li S (2006) Symphytognathidae (Araneae), a spider family newly recorded from China. Zootaxa 1259: 33–38.

[B52] Wheeler WC Coddington JA Crowley LM Dimitrov D Goloboff PA Griswold CE Hormiga G Prendini L Ramírez MJ Sierwald P Almeida-Silva L Alvarez-Padilla F Arnedo MA Benavides Silva LR Benjamin SP Bond JE Grismado CJ Hasan E Hedin M Izquierdo MA Labarque FM Ledford J Lopardo L Maddison WP Miller JA Piacentini LN Platnick NI Polotow D Silva-Dávila D Scharff N Szüts T Ubick D Vink CJ Wood HM Zhang J (2017) The spider tree of life: phylogeny of Araneae based on target-gene analyses from an extensive taxon sampling. Cladistics 33: 574–616. 10.1111/cla.12182 34724759

[B53] WSC (2020) World Spider Catalog Version 21.0. Natural History Museum Bern. http://wsc.nmbe.ch

[B54] Wunderlich J (1995) Drei bisher unbekannte Arten und Gattungen der Familie Anapidae (s.l.) aus Süd-Afrika, Brasilien und Malaysia (Arachnida: Araneae). Beiträge zur Araneologie 3: 543–551.

[B55] Wunderlich J (2004) The fossil spiders of the family *Anapidae* s. l. (Aeaneae) [sic] in Baltic, Dominican and Mexican amber and their extant relatives, with the description of the new subfamily Comarominae. Beiträge zur Araneologie 3: 1020–1111.

