# Supplementary figures and images for: First records and three new species of the family Symphytognathidae (Arachnida, Araneae) from Thailand, and the circumscription of the genus Crassignatha Wunderlich, 1995
Source: Zookeys. 2021 Jan 26;1012:21–53. doi: 10.3897/zookeys.1012.57047 (PMC7854560; doi:10.3897/zookeys.1012.57047)

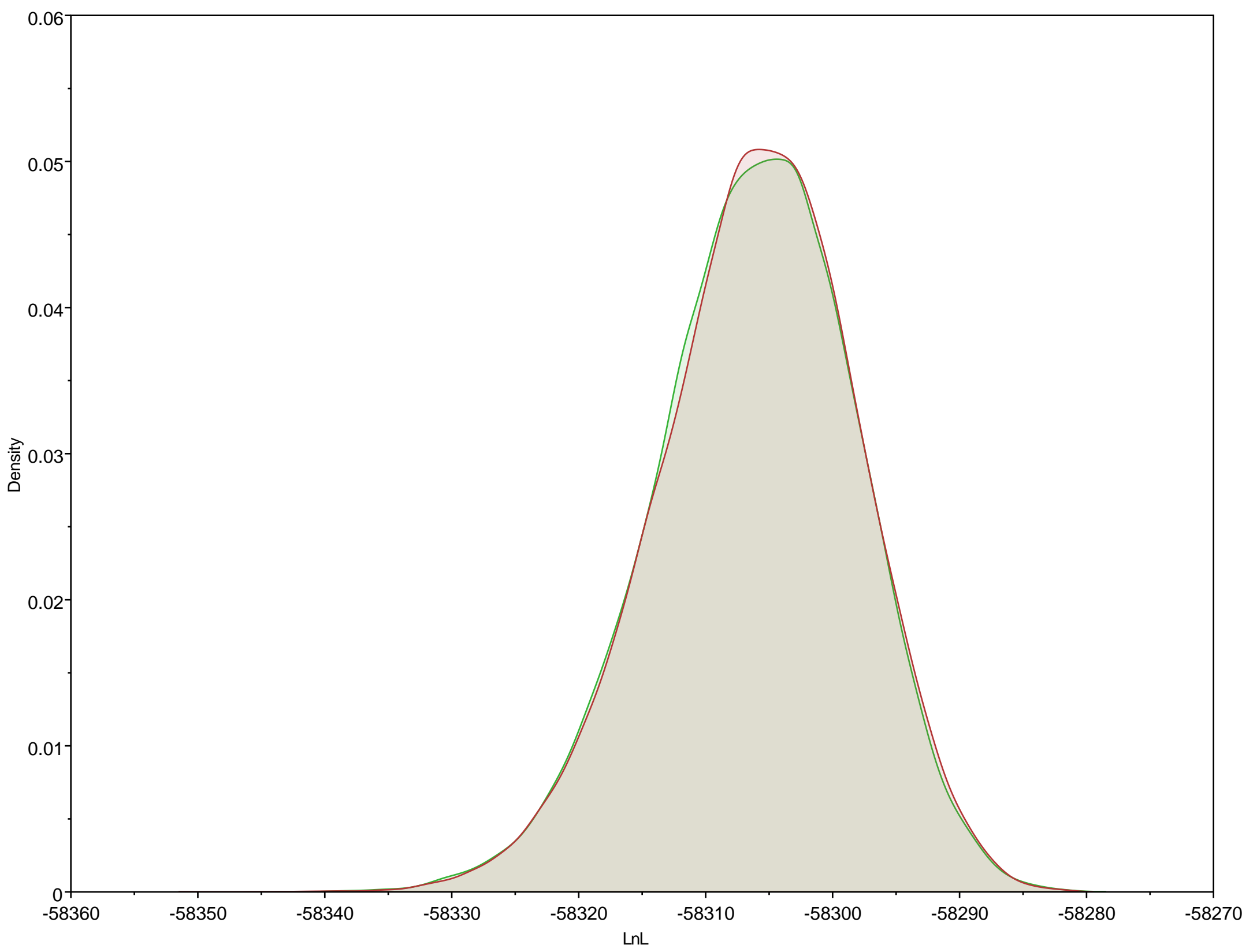

Supplement: Supplementary material 1 — List of primers used in our study [file zookeys-1012-021-s001.zip › Supplementary material 1/Thai2018_Symphy_Likelihood_histogram_BI.pdf]

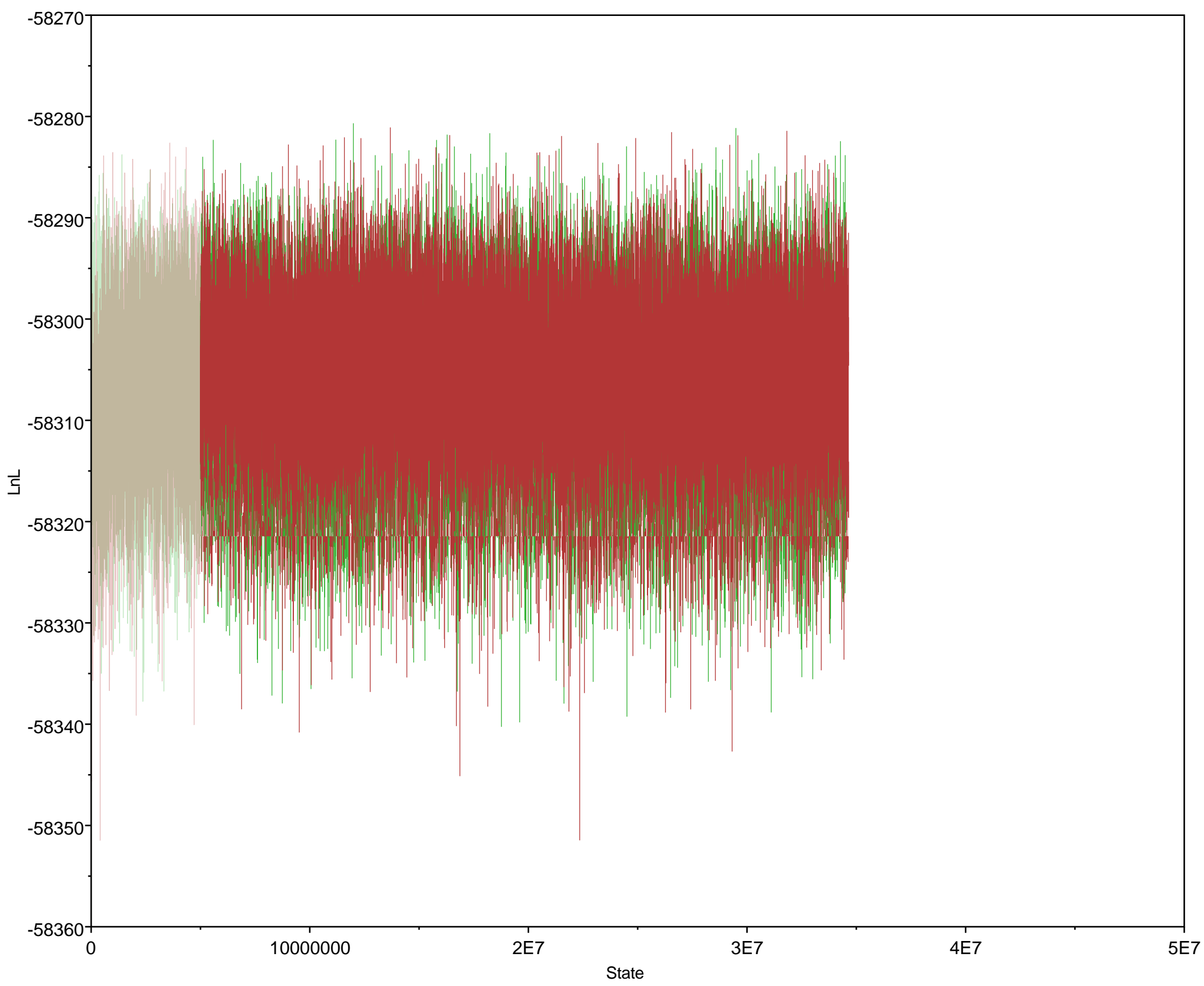

Supplement: Supplementary material 1 — List of primers used in our study [file zookeys-1012-021-s001.zip › Supplementary material 1/Thai2018_Symphy_Trace_plot_BI.pdf]

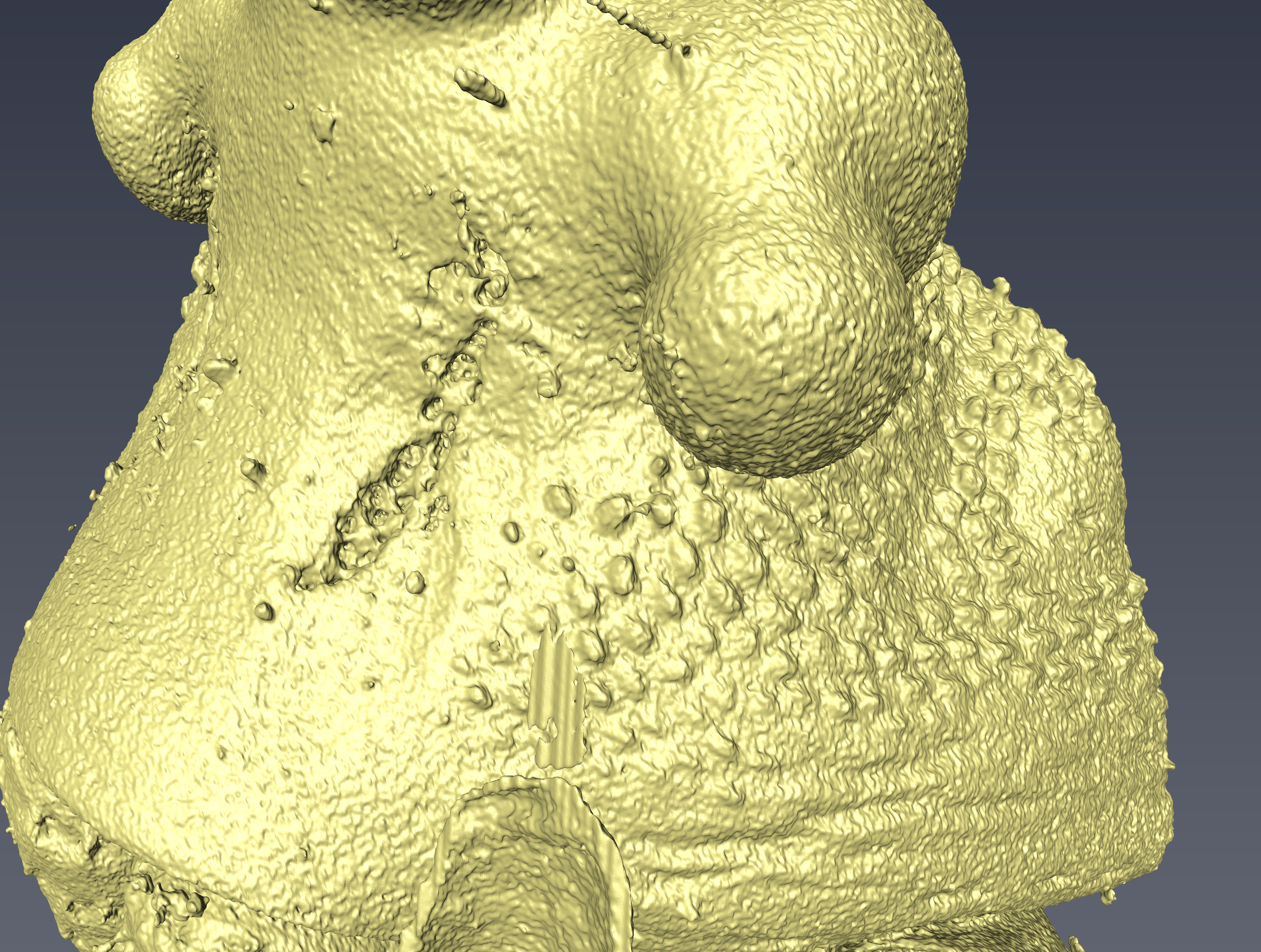

Supplement: Supplementary material 2 — 3D reconstructions Crassignatha seeliam sp. nov. male pedipalp and habitus [file zookeys-1012-021-s002.zip › Supplementary material 2/Crassignatha_seeliam_carapace_antero_lateral_surface.jpg]

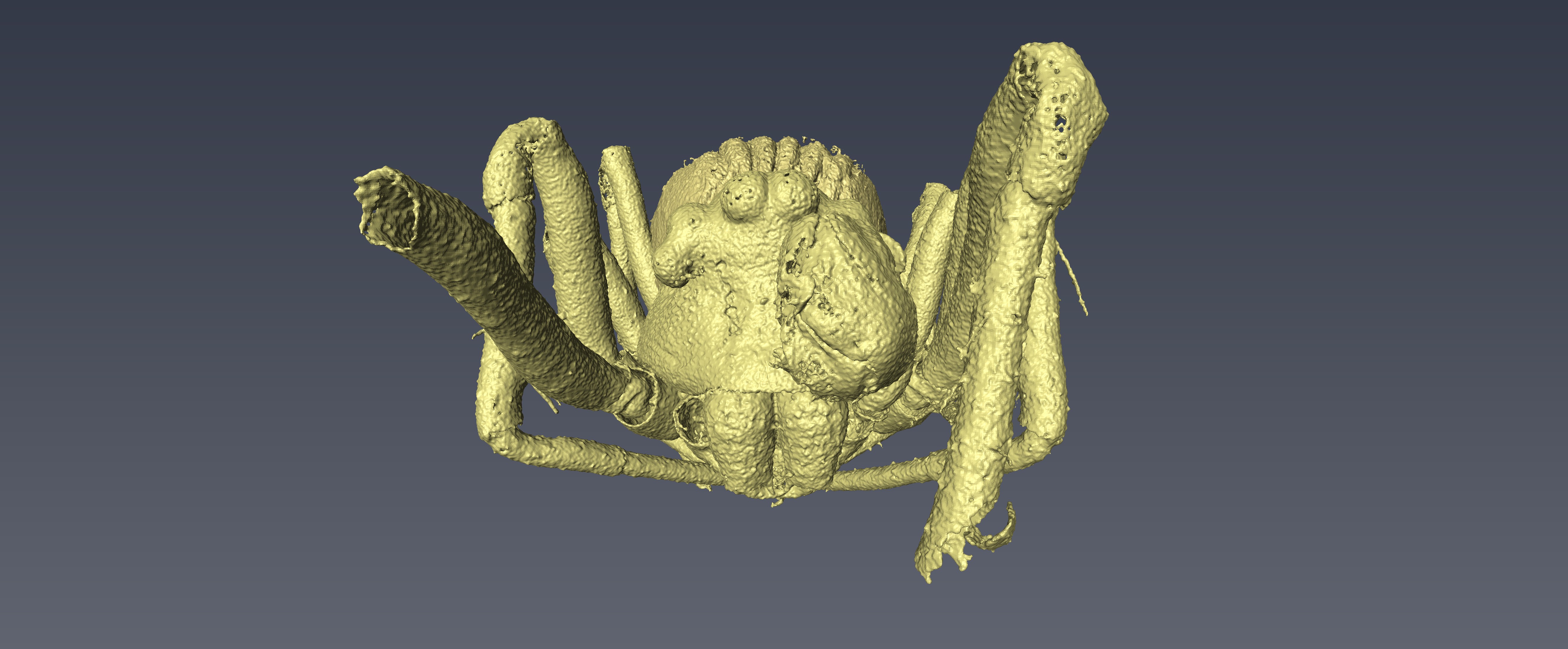

Supplement: Supplementary material 2 — 3D reconstructions Crassignatha seeliam sp. nov. male pedipalp and habitus [file zookeys-1012-021-s002.zip › Supplementary material 2/Crassignatha_seeliam_habitus_anterior_surface.jpg]

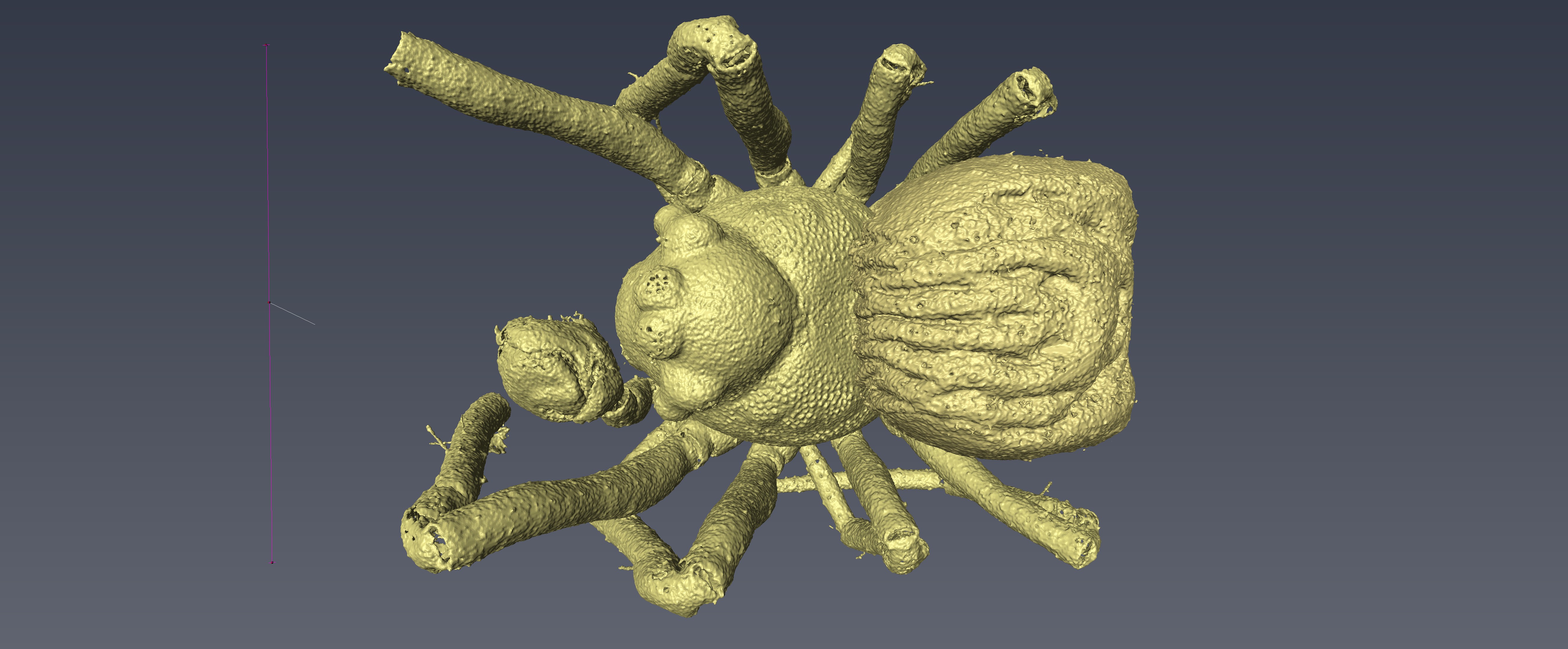

Supplement: Supplementary material 2 — 3D reconstructions Crassignatha seeliam sp. nov. male pedipalp and habitus [file zookeys-1012-021-s002.zip › Supplementary material 2/Crassignatha_seeliam_habitus_dorsal_surface.jpg]

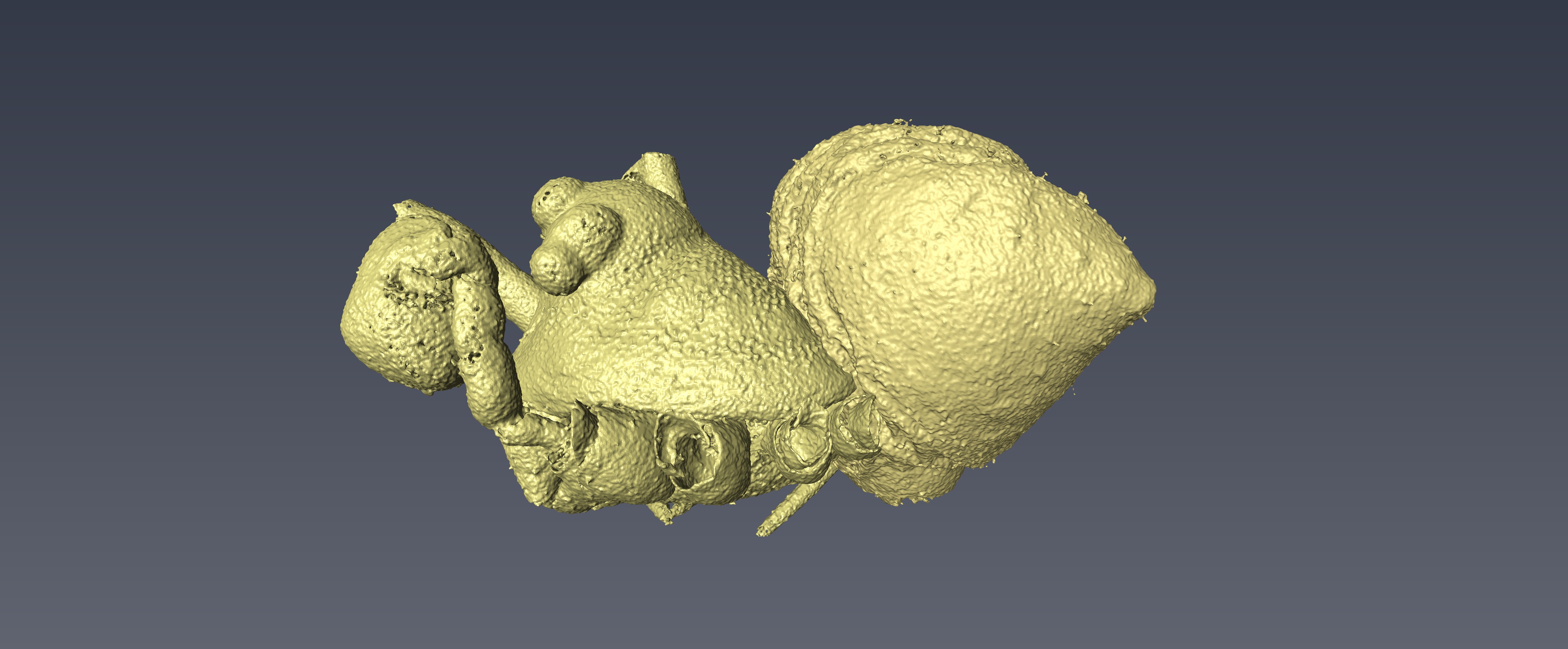

Supplement: Supplementary material 2 — 3D reconstructions Crassignatha seeliam sp. nov. male pedipalp and habitus [file zookeys-1012-021-s002.zip › Supplementary material 2/Crassignatha_seeliam_habitus_lateral_surface.jpg]

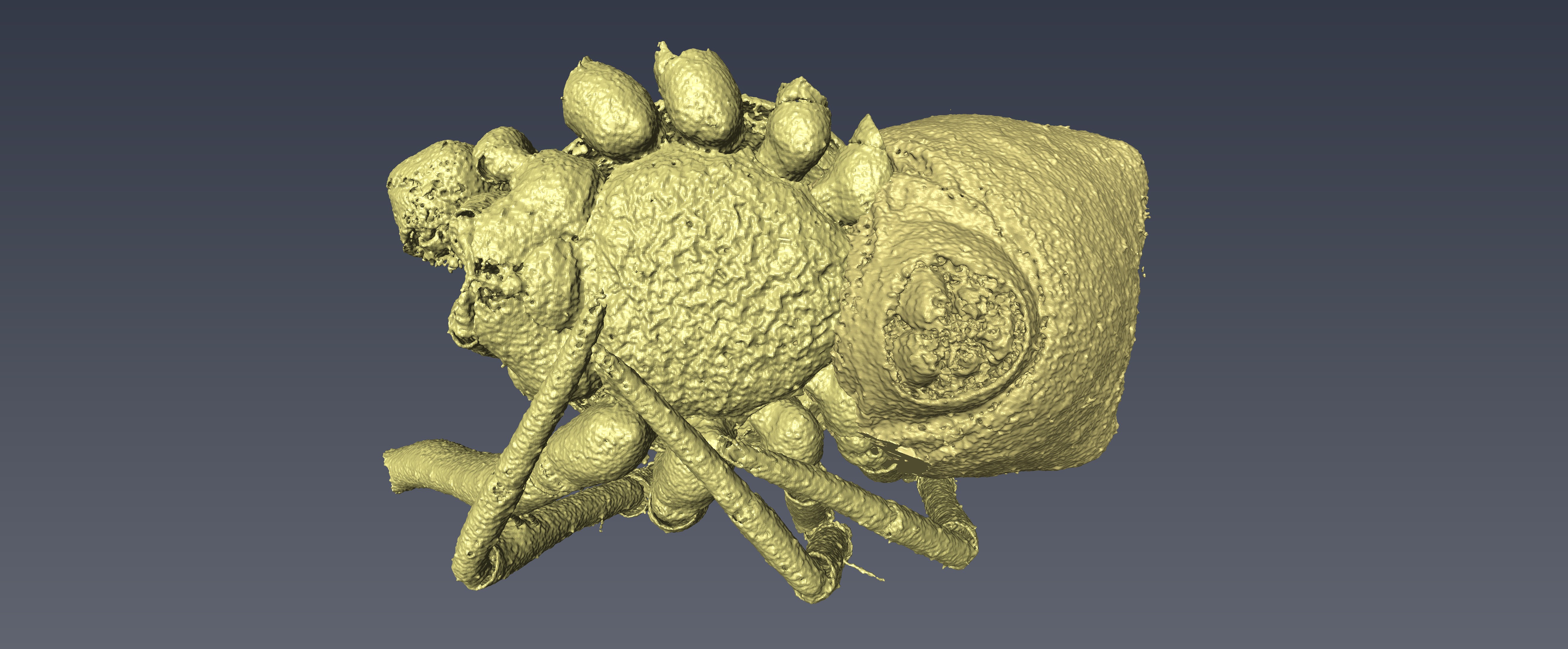

Supplement: Supplementary material 2 — 3D reconstructions Crassignatha seeliam sp. nov. male pedipalp and habitus [file zookeys-1012-021-s002.zip › Supplementary material 2/Crassignatha_seeliam_habitus_ventral_surface.jpg]

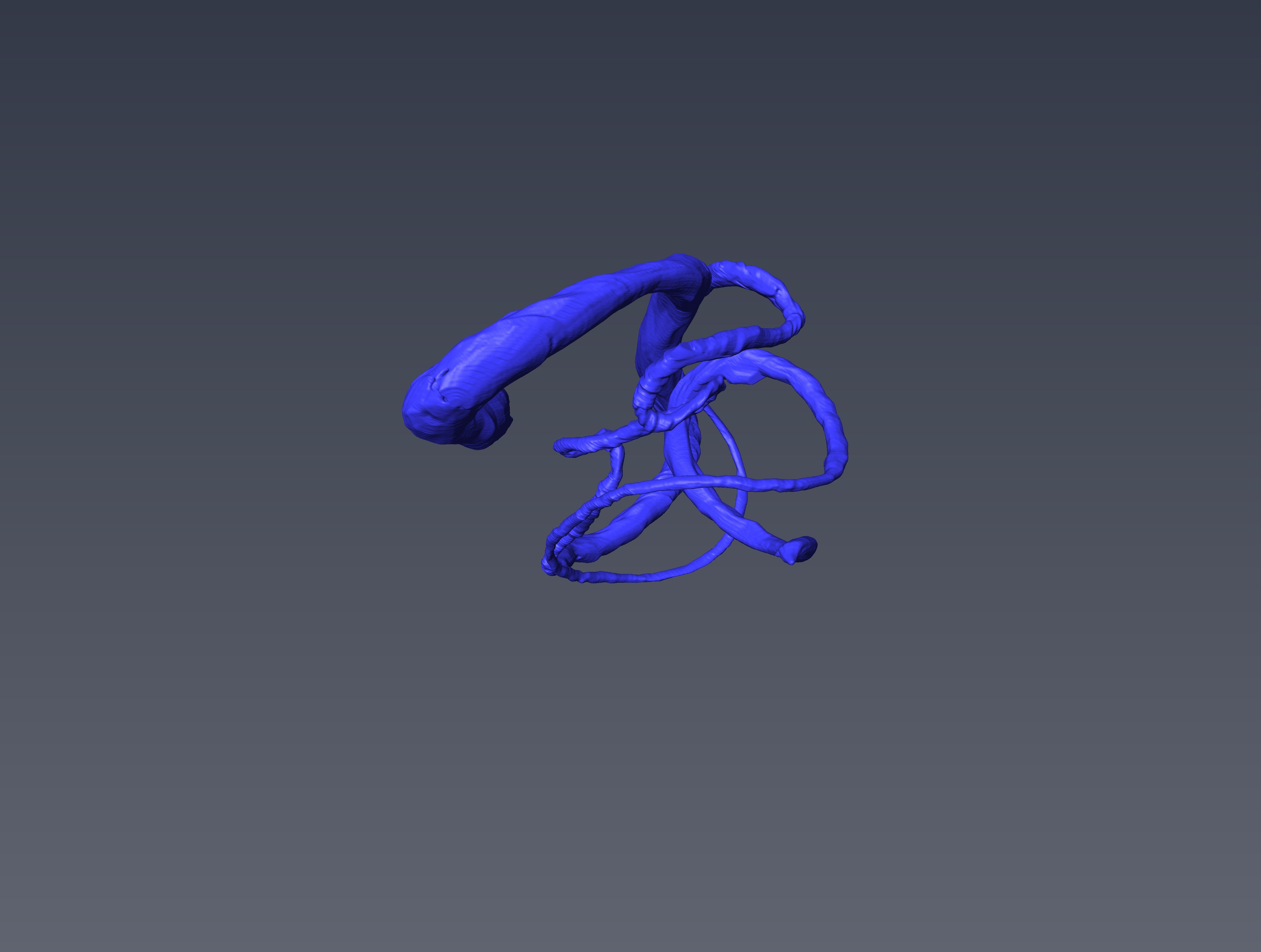

Supplement: Supplementary material 2 — 3D reconstructions Crassignatha seeliam sp. nov. male pedipalp and habitus [file zookeys-1012-021-s002.zip › Supplementary material 2/Crassignatha_seeliam_palp_anterior_ducts.jpg]

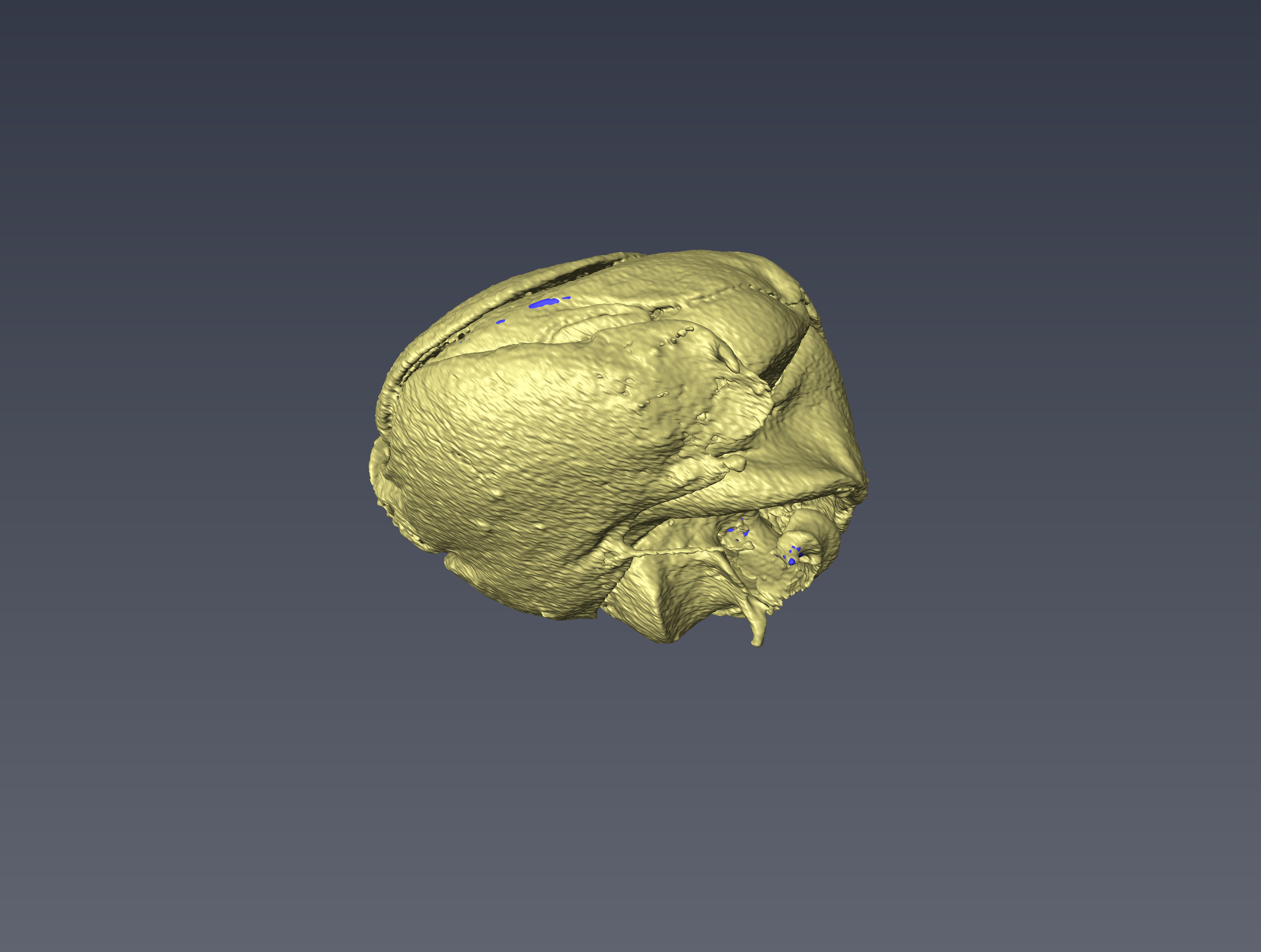

Supplement: Supplementary material 2 — 3D reconstructions Crassignatha seeliam sp. nov. male pedipalp and habitus [file zookeys-1012-021-s002.zip › Supplementary material 2/Crassignatha_seeliam_palp_anterior_surface.jpg]

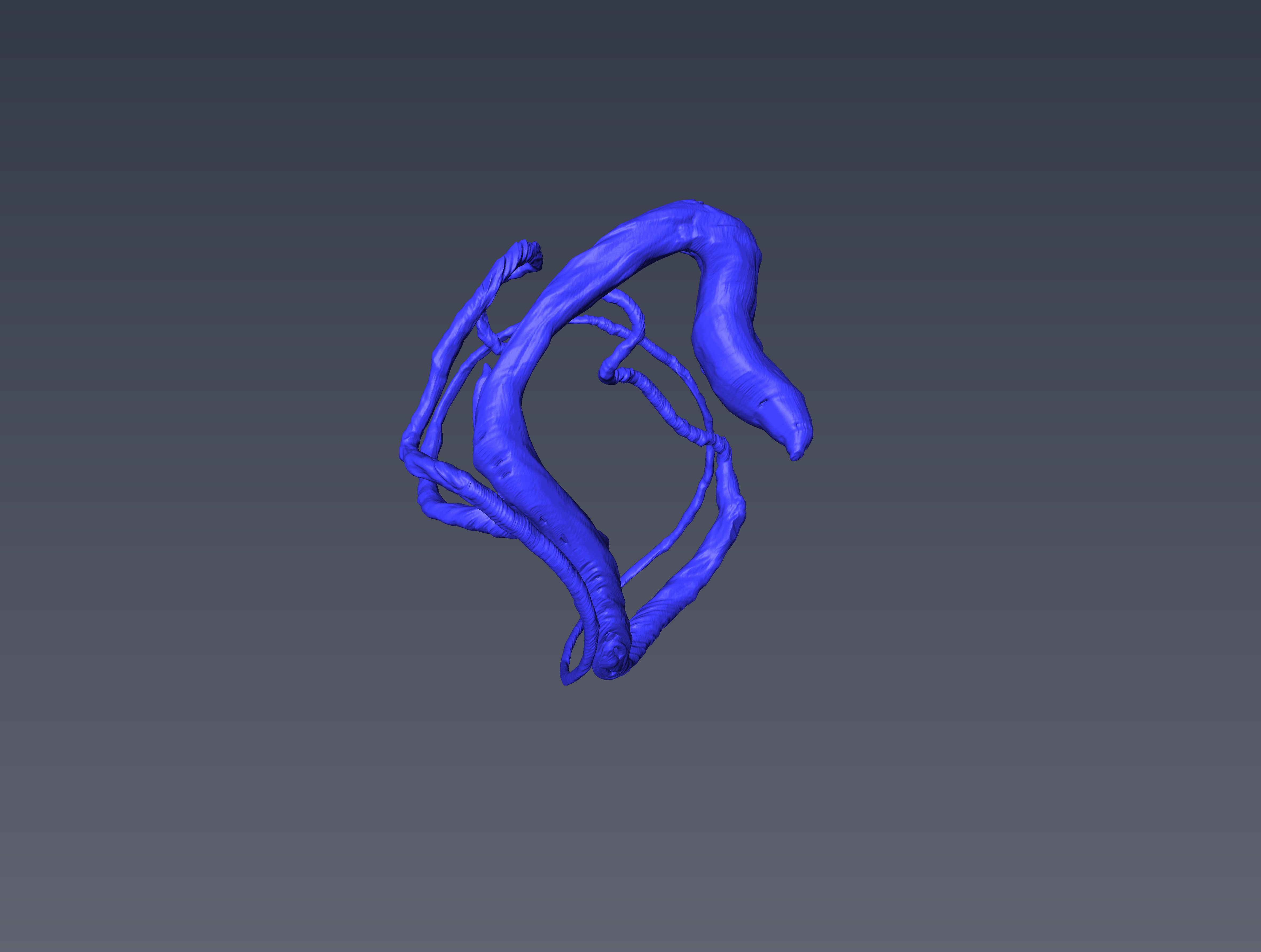

Supplement: Supplementary material 2 — 3D reconstructions Crassignatha seeliam sp. nov. male pedipalp and habitus [file zookeys-1012-021-s002.zip › Supplementary material 2/Crassignatha_seeliam_palp_dorsal_ducts.jpg]

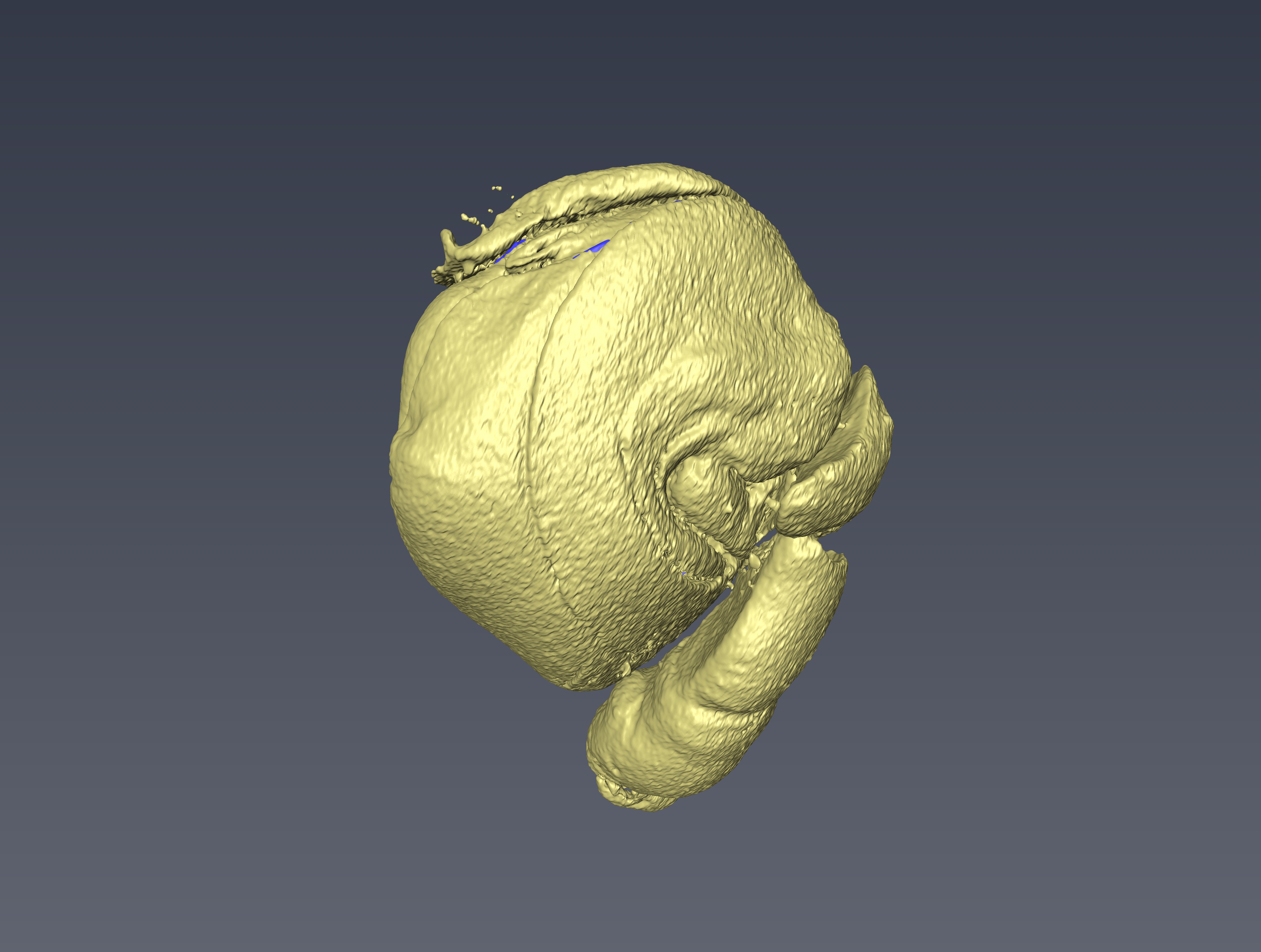

Supplement: Supplementary material 2 — 3D reconstructions Crassignatha seeliam sp. nov. male pedipalp and habitus [file zookeys-1012-021-s002.zip › Supplementary material 2/Crassignatha_seeliam_palp_dorsal_surface.jpg]

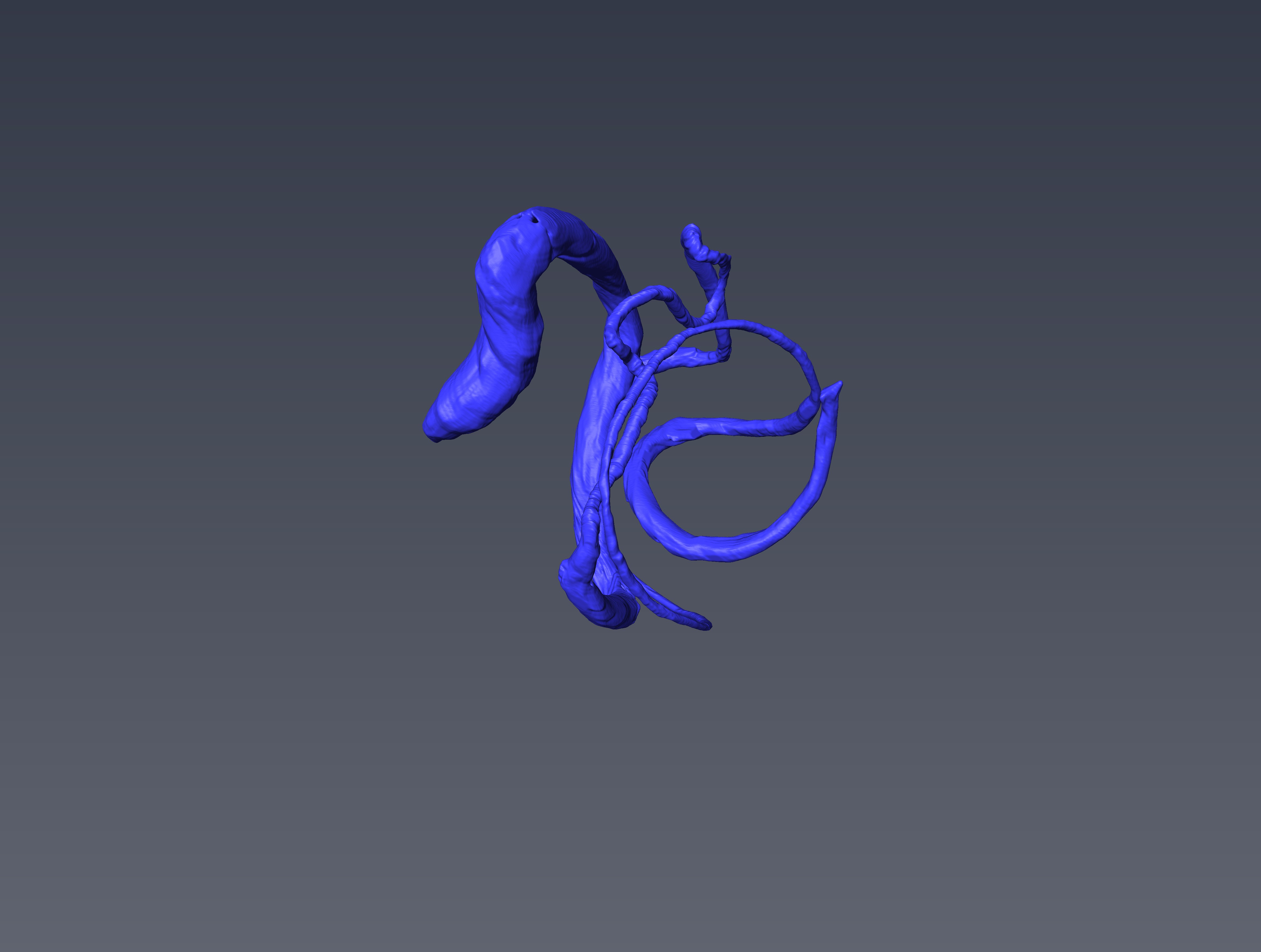

Supplement: Supplementary material 2 — 3D reconstructions Crassignatha seeliam sp. nov. male pedipalp and habitus [file zookeys-1012-021-s002.zip › Supplementary material 2/Crassignatha_seeliam_palp_prolateral_ducts.jpg]

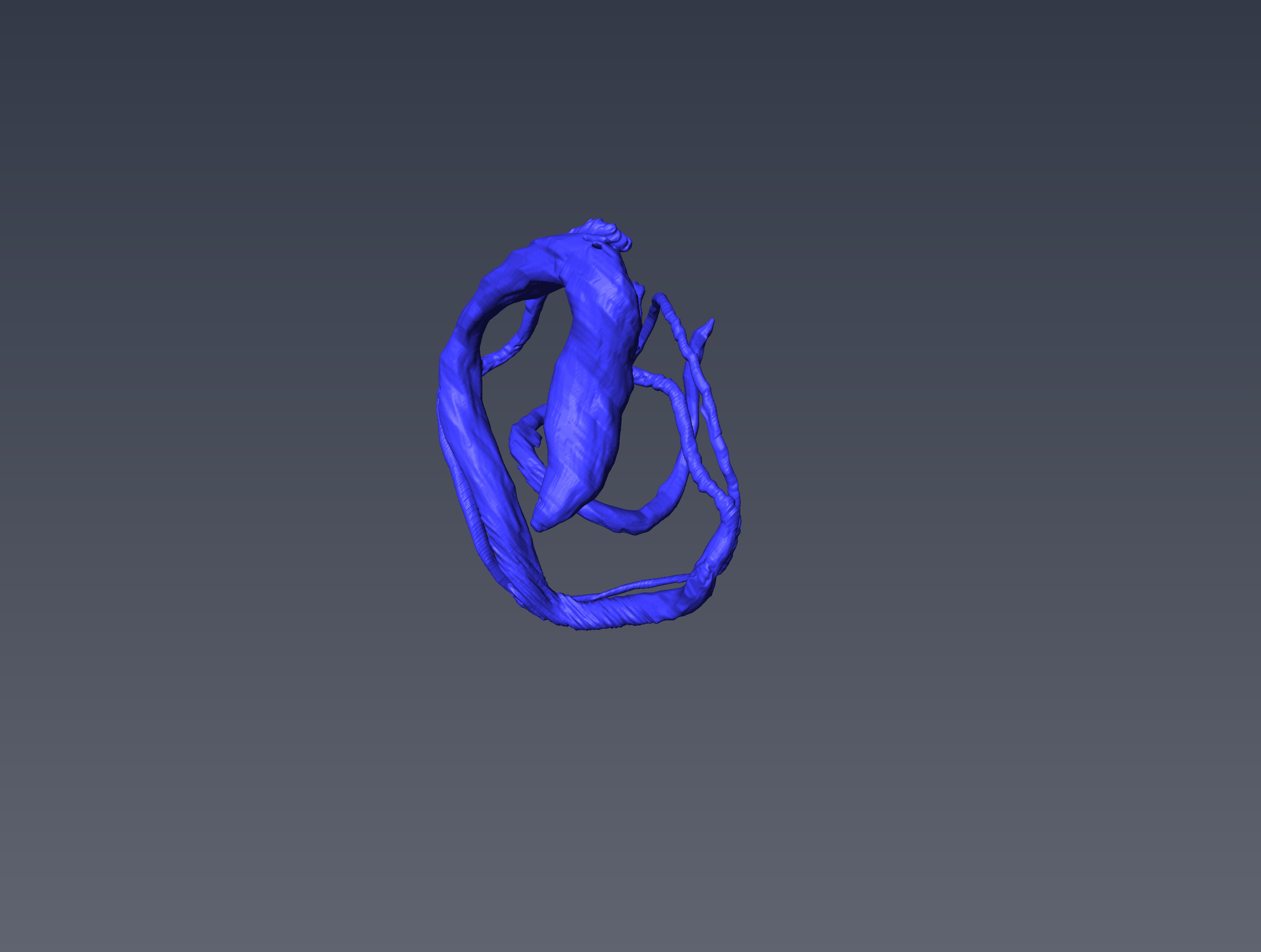

Supplement: Supplementary material 2 — 3D reconstructions Crassignatha seeliam sp. nov. male pedipalp and habitus [file zookeys-1012-021-s002.zip › Supplementary material 2/Crassignatha_seeliam_palp_prolateral_ducts_1.jpg]

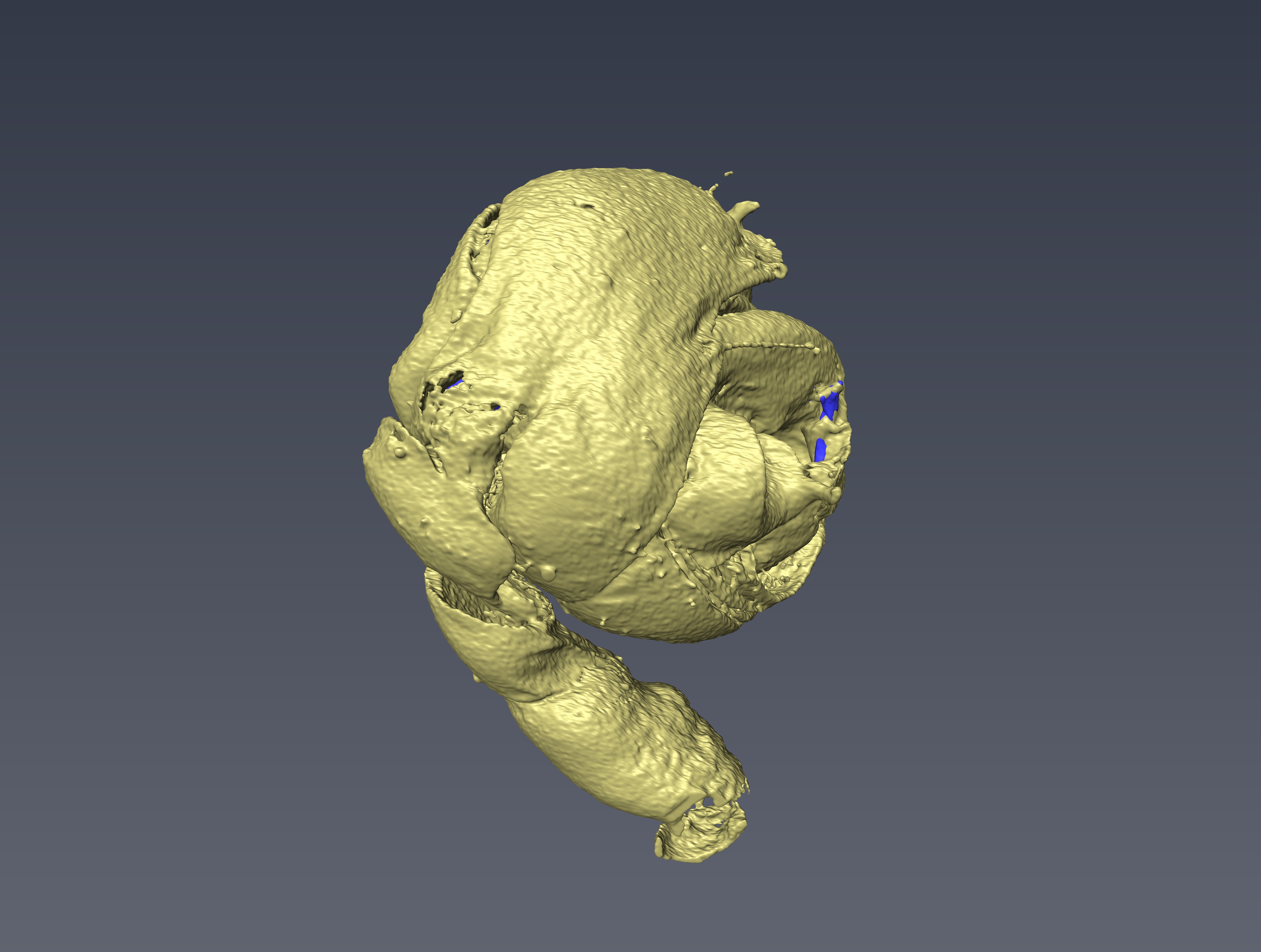

Supplement: Supplementary material 2 — 3D reconstructions Crassignatha seeliam sp. nov. male pedipalp and habitus [file zookeys-1012-021-s002.zip › Supplementary material 2/Crassignatha_seeliam_palp_prolateral_surface.jpg]

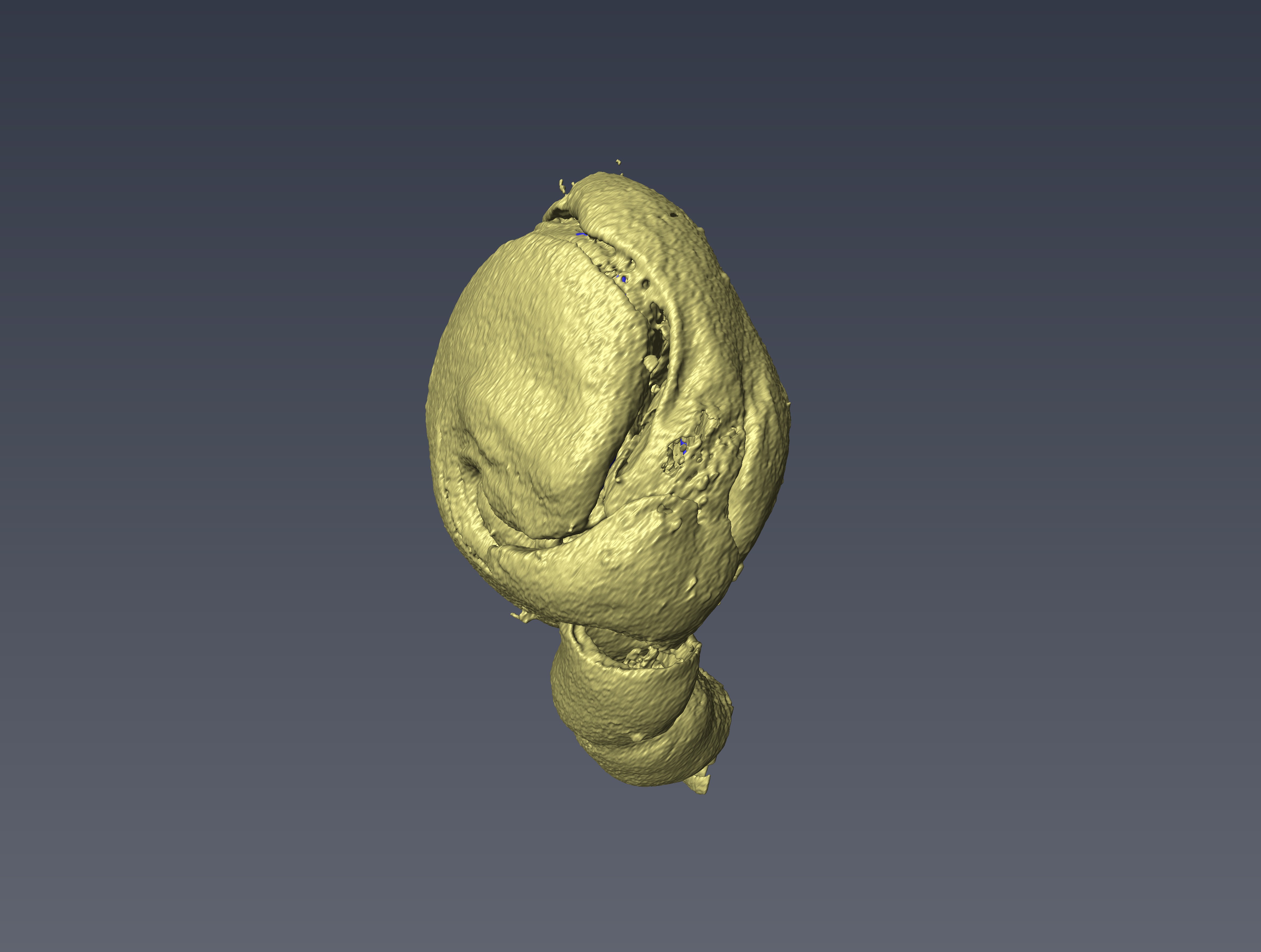

Supplement: Supplementary material 2 — 3D reconstructions Crassignatha seeliam sp. nov. male pedipalp and habitus [file zookeys-1012-021-s002.zip › Supplementary material 2/Crassignatha_seeliam_palp_prolateral_surface_1.jpg]

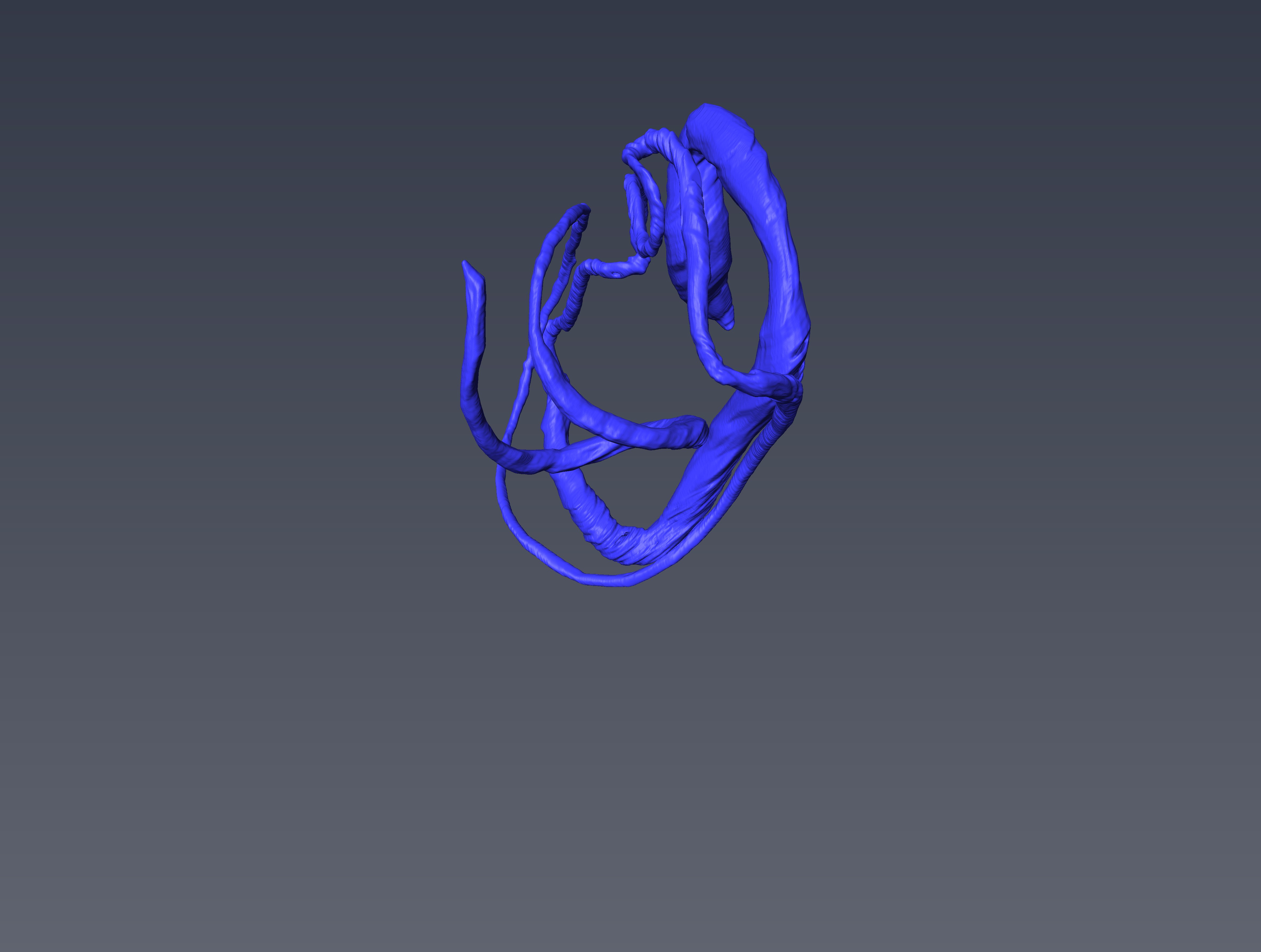

Supplement: Supplementary material 2 — 3D reconstructions Crassignatha seeliam sp. nov. male pedipalp and habitus [file zookeys-1012-021-s002.zip › Supplementary material 2/Crassignatha_seeliam_palp_retrolateral_ducts.jpg]

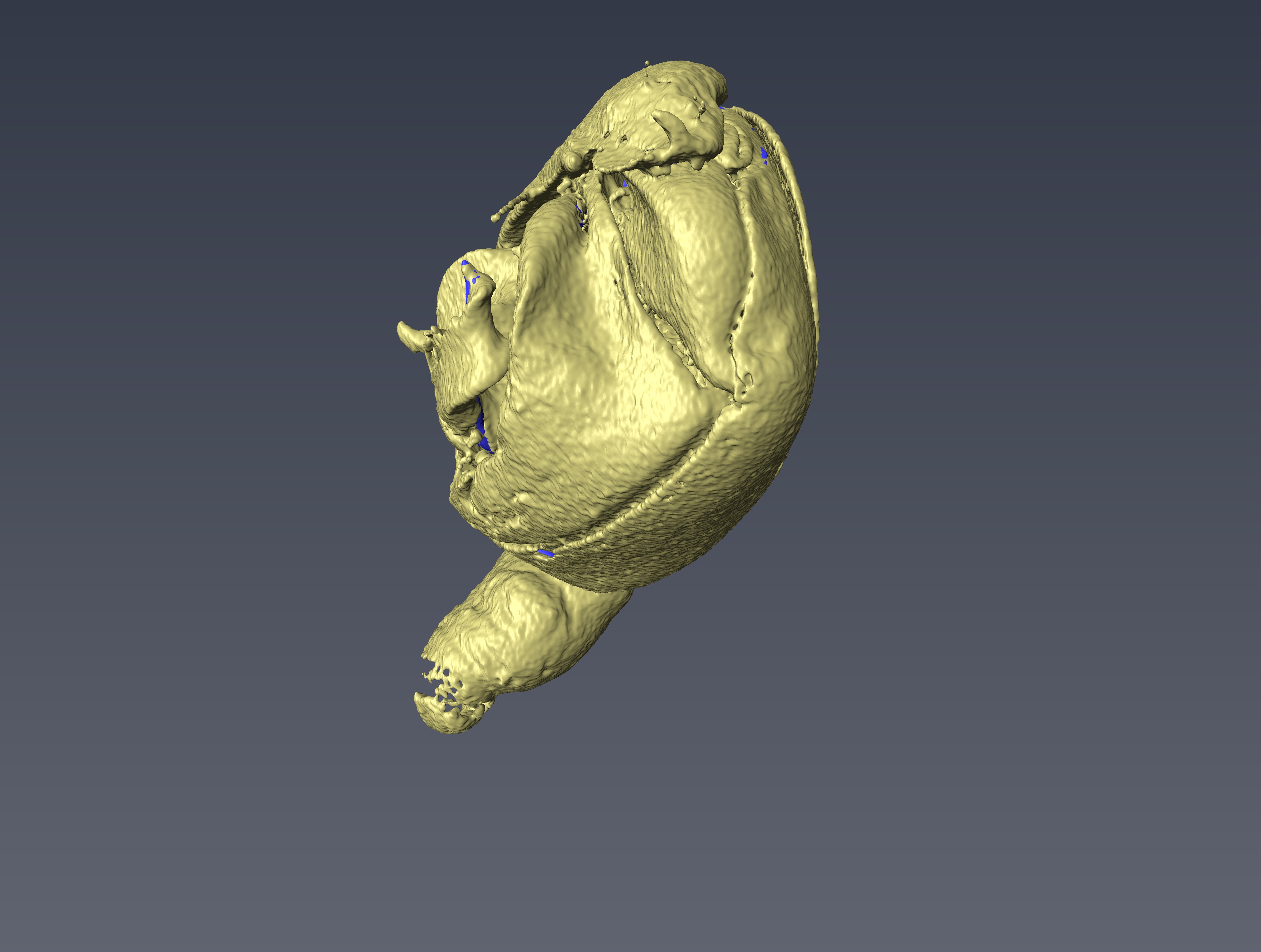

Supplement: Supplementary material 2 — 3D reconstructions Crassignatha seeliam sp. nov. male pedipalp and habitus [file zookeys-1012-021-s002.zip › Supplementary material 2/Crassignatha_seeliam_palp_retrolateral_surface.jpg]

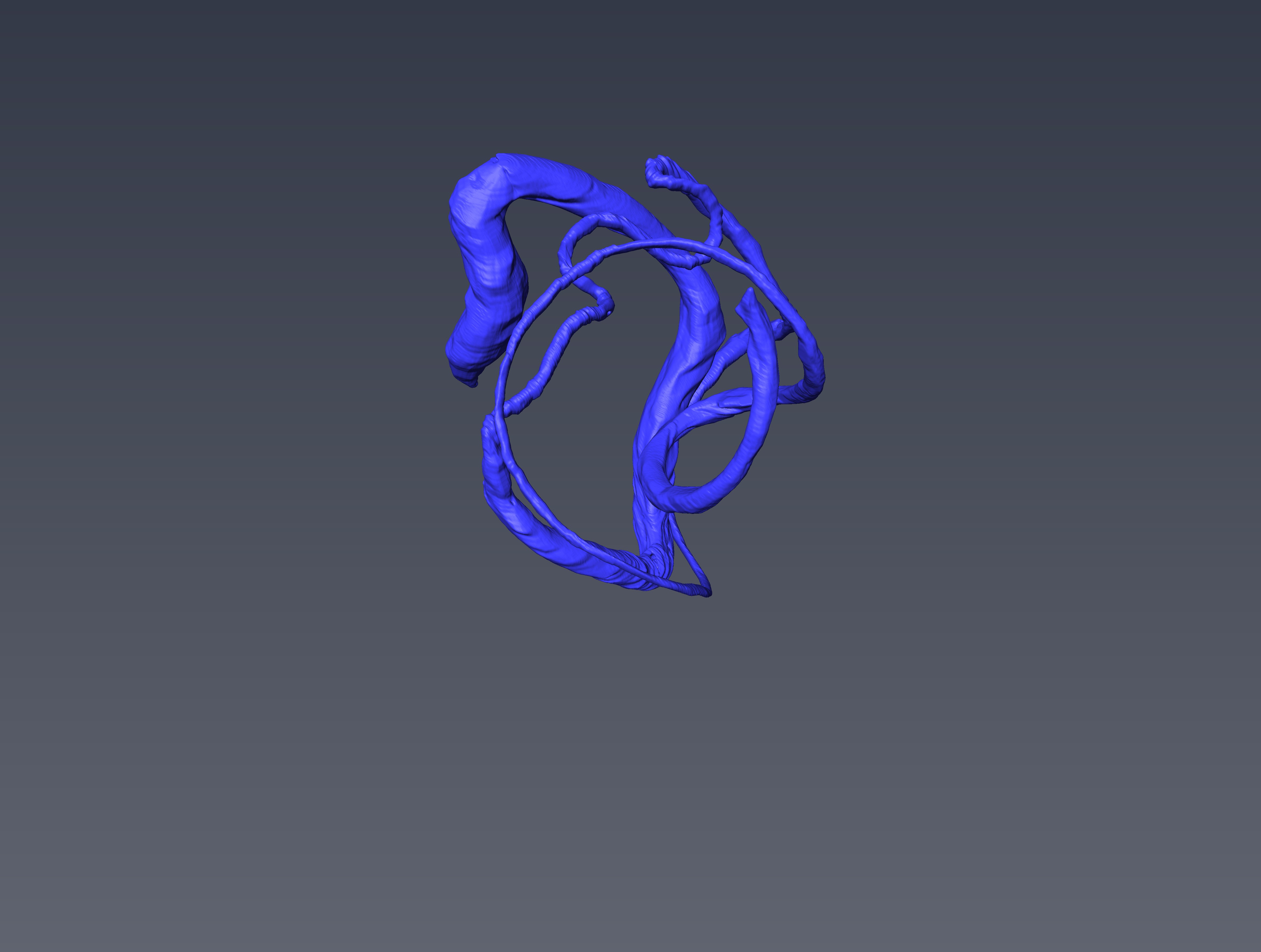

Supplement: Supplementary material 2 — 3D reconstructions Crassignatha seeliam sp. nov. male pedipalp and habitus [file zookeys-1012-021-s002.zip › Supplementary material 2/Crassignatha_seeliam_palp_ventral_ducts.jpg]

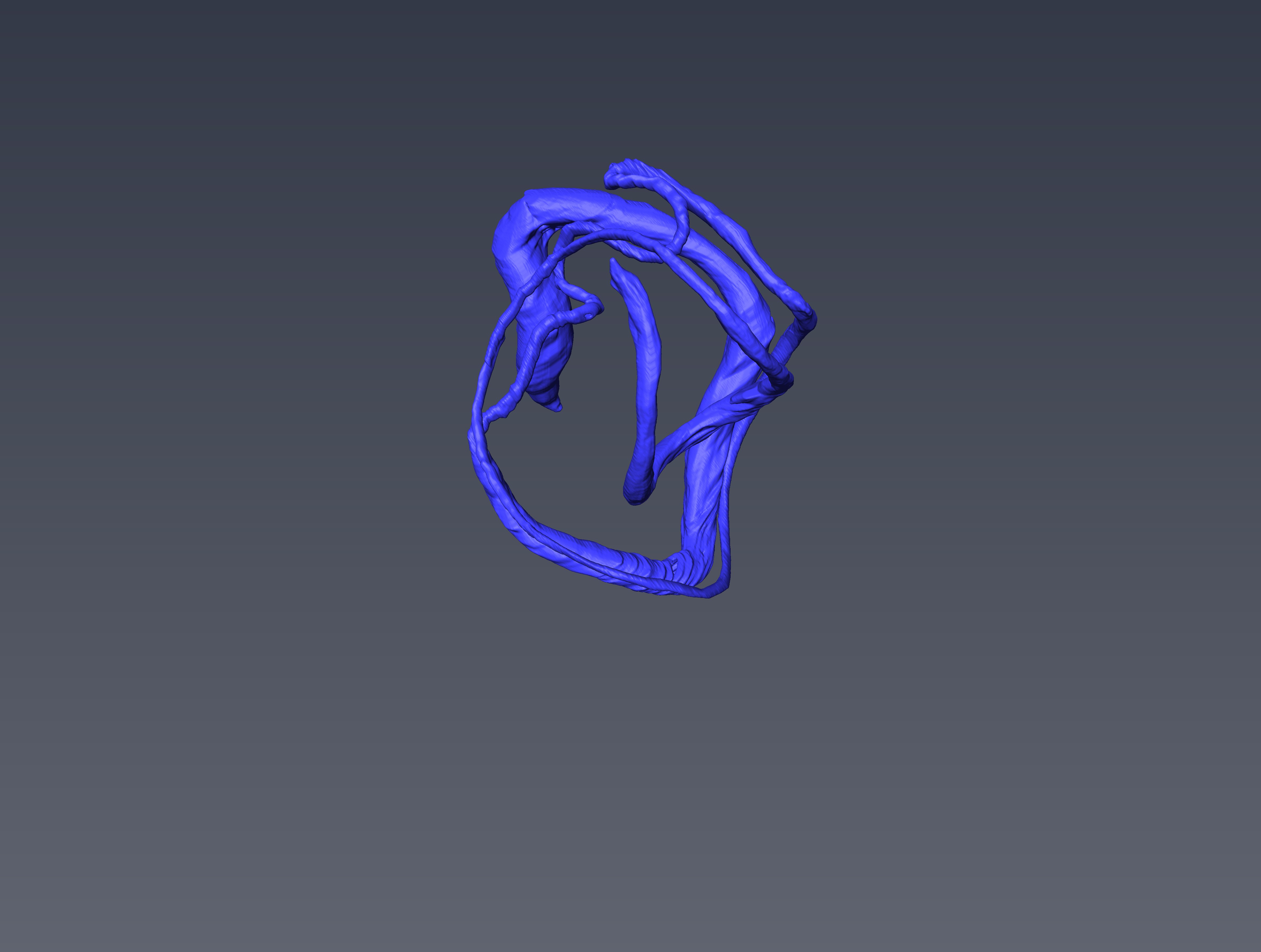

Supplement: Supplementary material 2 — 3D reconstructions Crassignatha seeliam sp. nov. male pedipalp and habitus [file zookeys-1012-021-s002.zip › Supplementary material 2/Crassignatha_seeliam_palp_ventral_ducts_1.jpg]

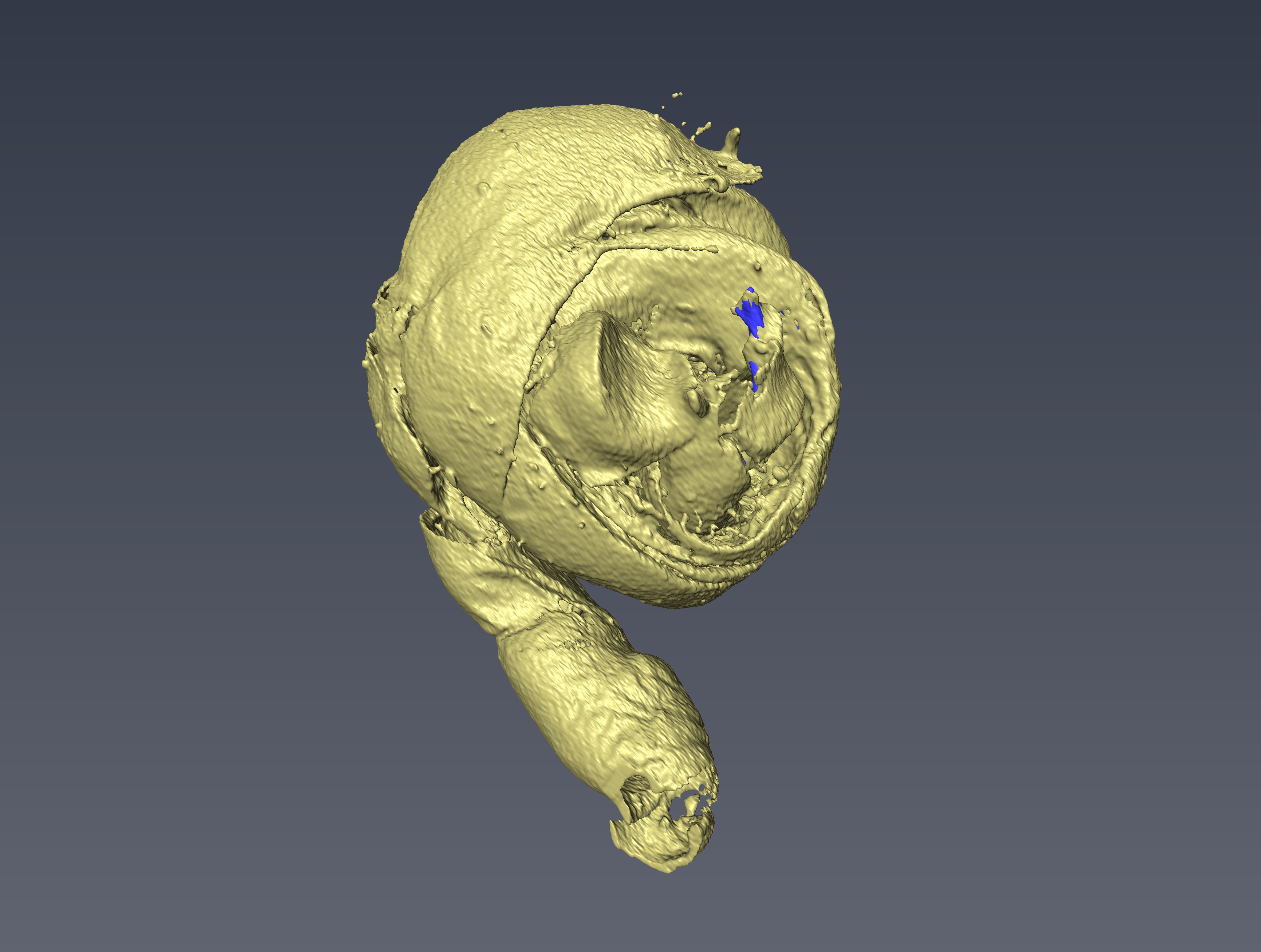

Supplement: Supplementary material 2 — 3D reconstructions Crassignatha seeliam sp. nov. male pedipalp and habitus [file zookeys-1012-021-s002.zip › Supplementary material 2/Crassignatha_seeliam_palp_ventral_surface.jpg]

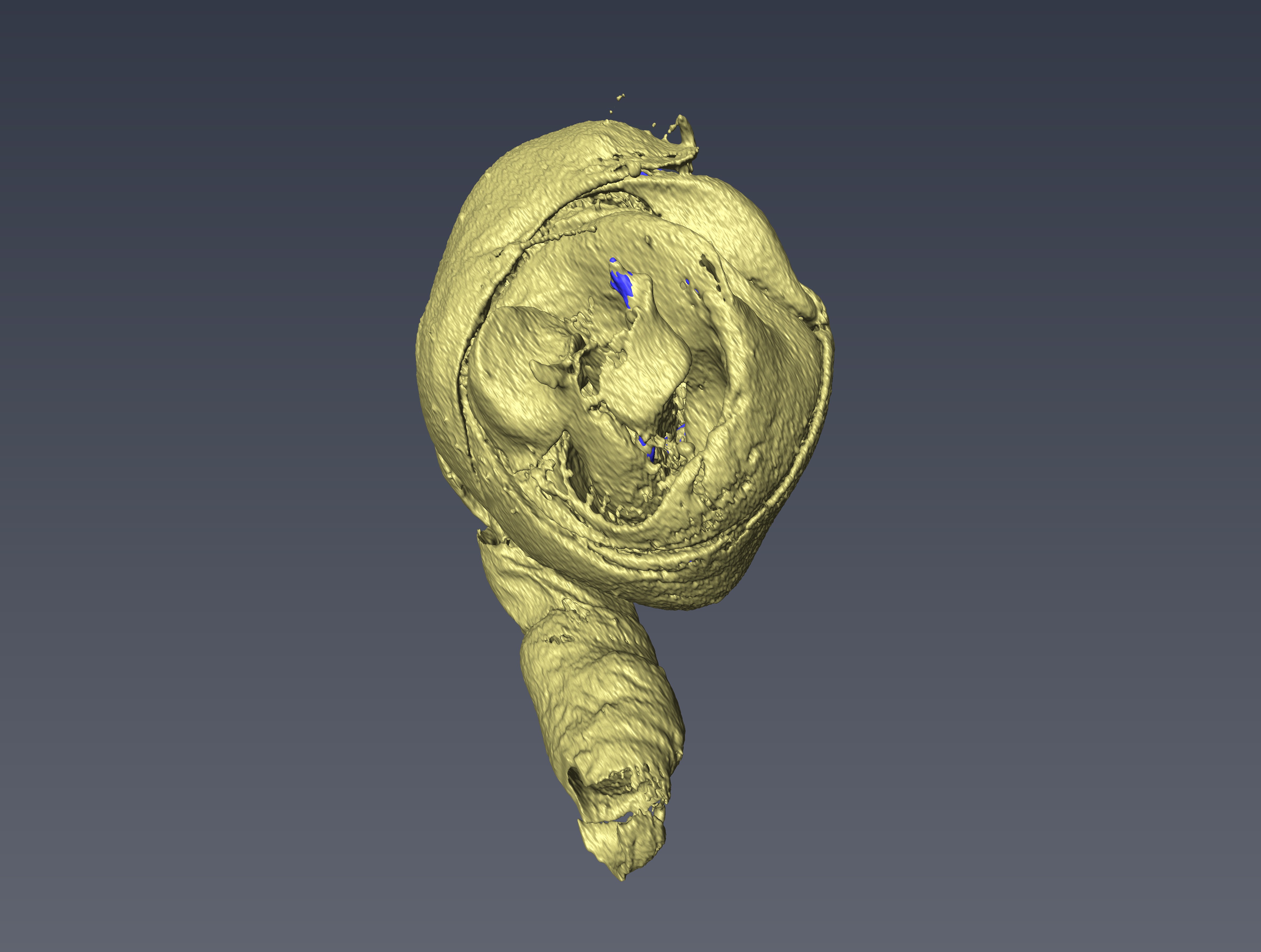

Supplement: Supplementary material 2 — 3D reconstructions Crassignatha seeliam sp. nov. male pedipalp and habitus [file zookeys-1012-021-s002.zip › Supplementary material 2/Crassignatha_seeliam_palp_ventral_surface_1.jpg]

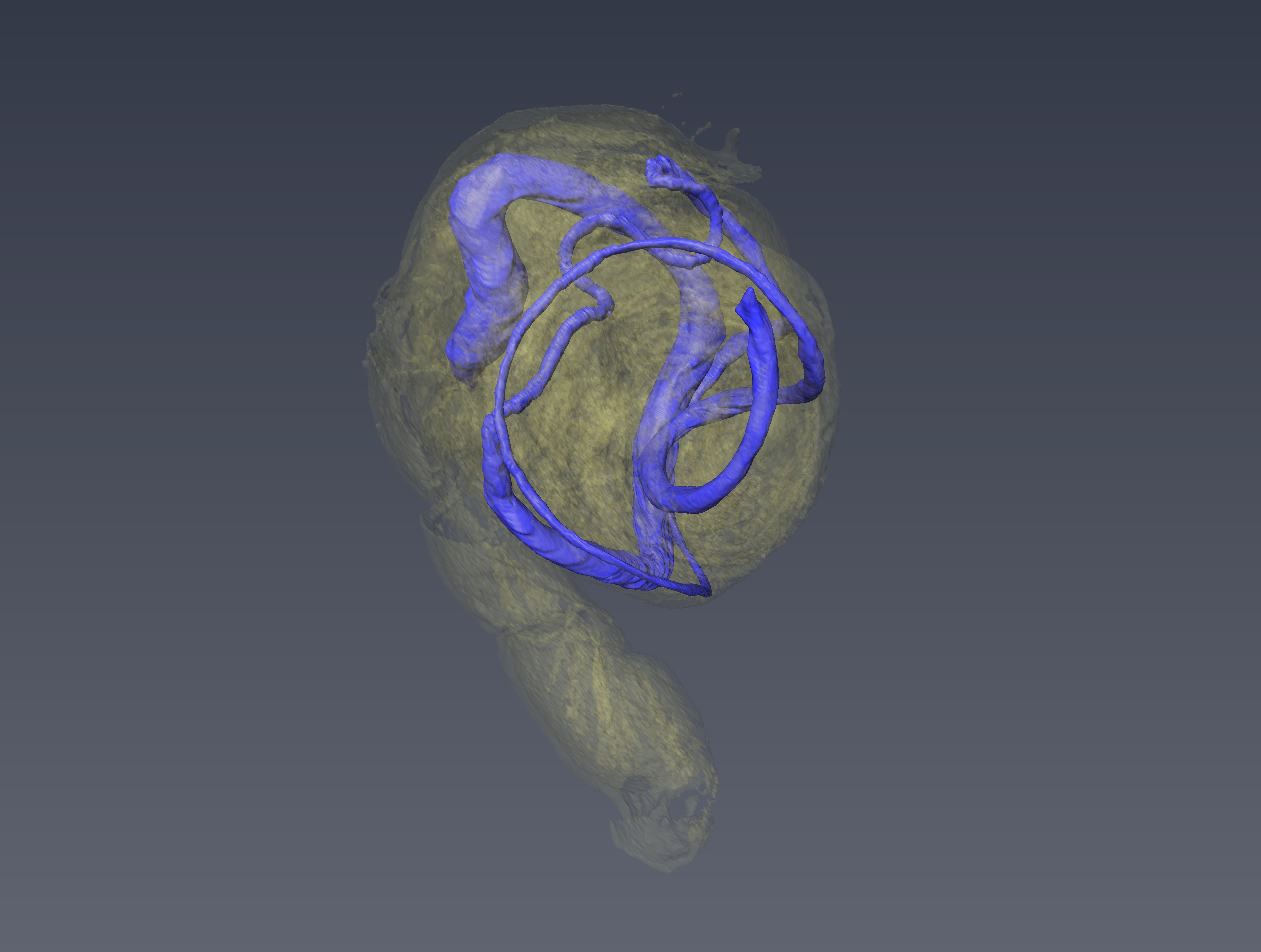

Supplement: Supplementary material 2 — 3D reconstructions Crassignatha seeliam sp. nov. male pedipalp and habitus [file zookeys-1012-021-s002.zip › Supplementary material 2/Crassignatha_seeliam_palp_ventral_transparency.jpg]

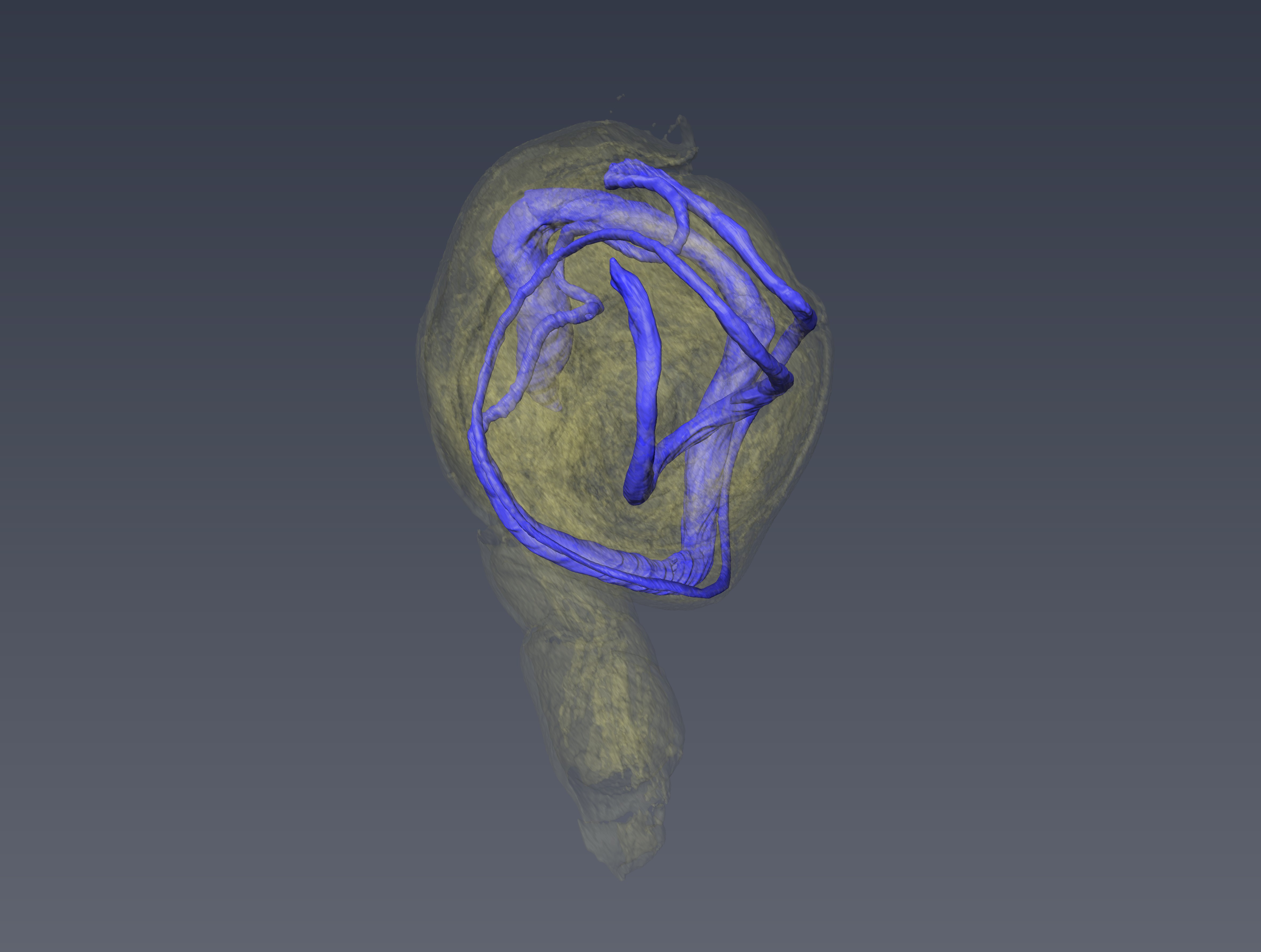

Supplement: Supplementary material 2 — 3D reconstructions Crassignatha seeliam sp. nov. male pedipalp and habitus [file zookeys-1012-021-s002.zip › Supplementary material 2/Crassignatha_seeliam_palp_ventral_transparency_1.jpg]

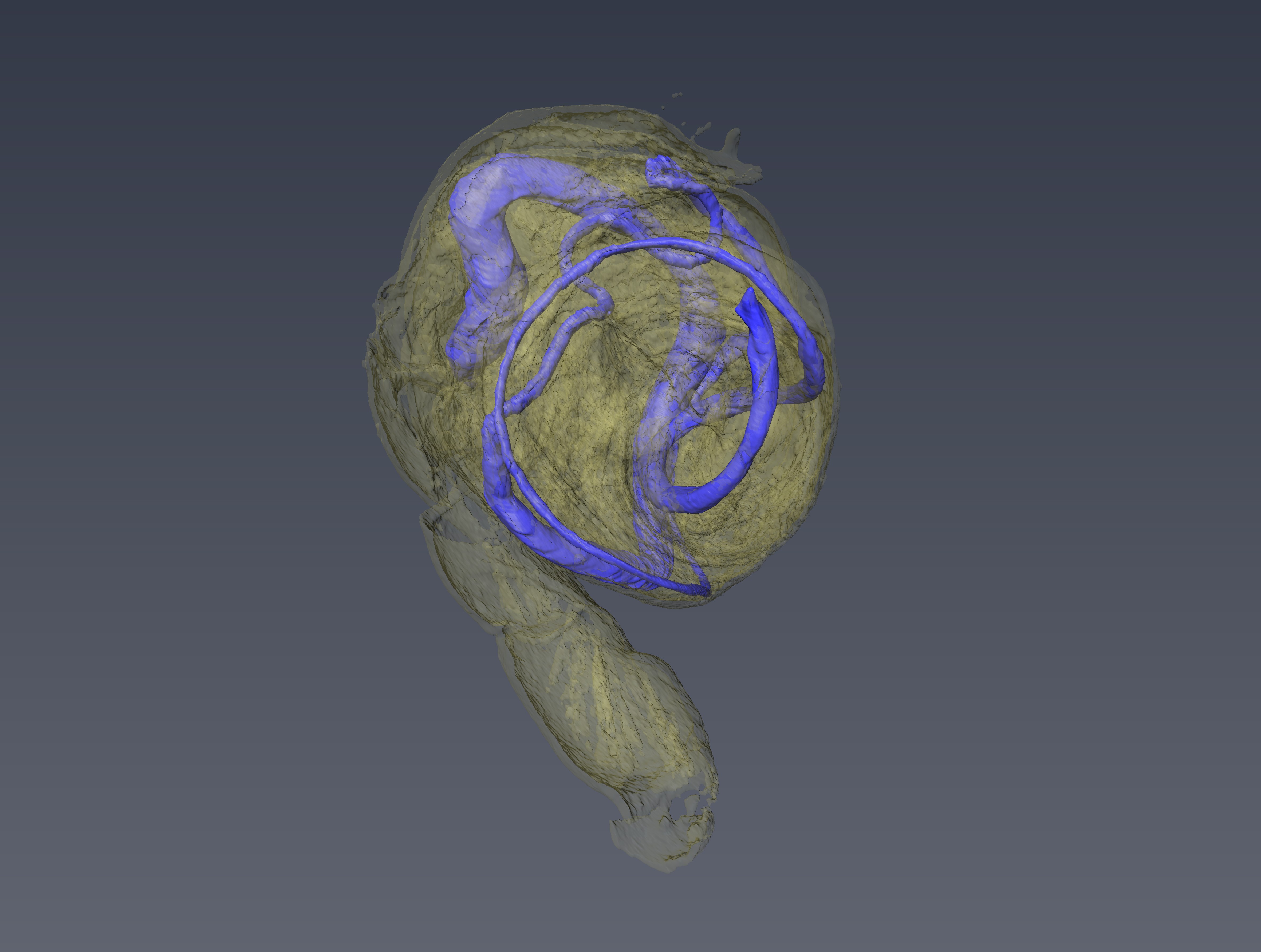

Supplement: Supplementary material 2 — 3D reconstructions Crassignatha seeliam sp. nov. male pedipalp and habitus [file zookeys-1012-021-s002.zip › Supplementary material 2/Crassignatha_seeliam_palp_ventral_transparency_2.jpg]

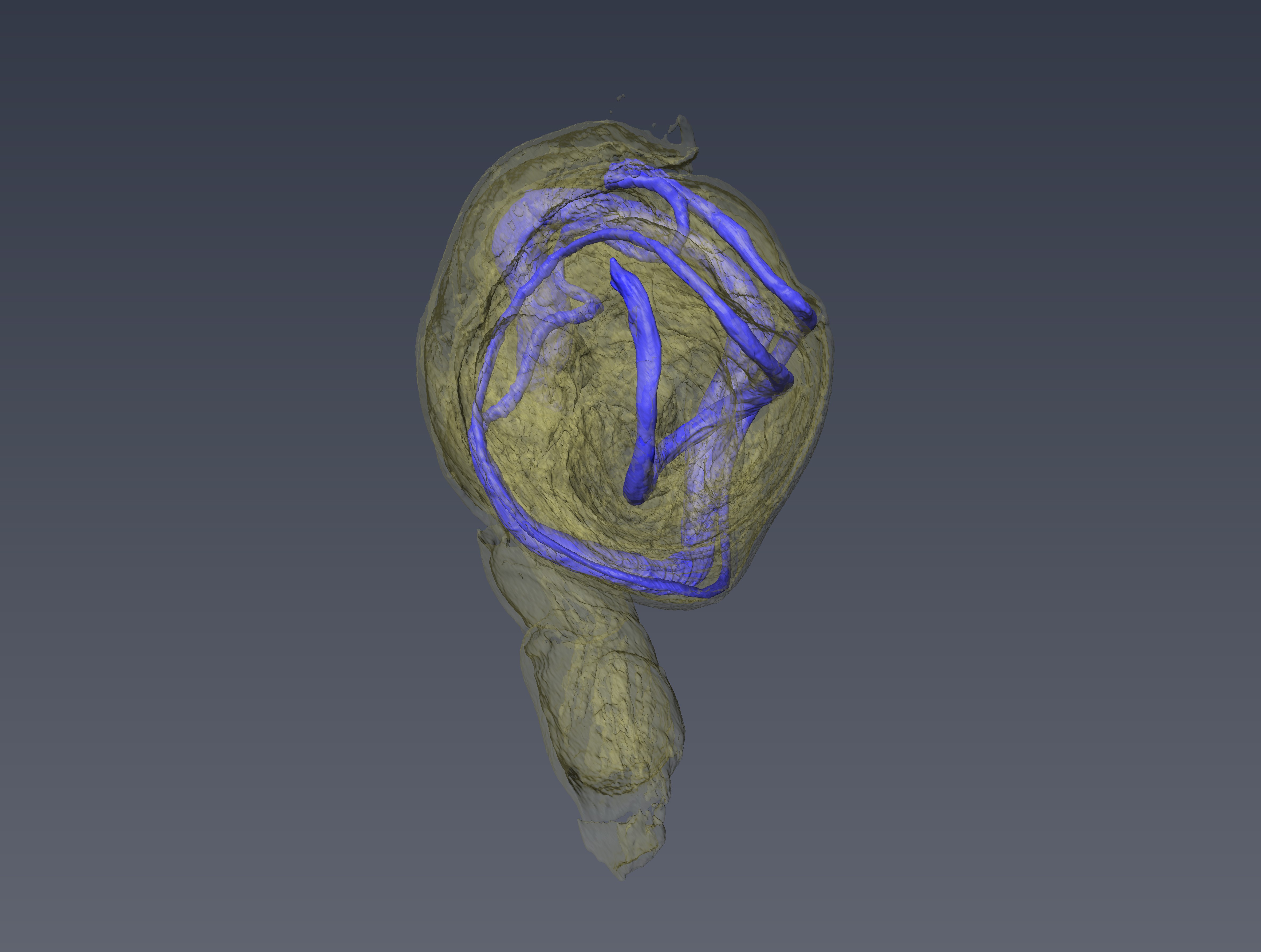

Supplement: Supplementary material 2 — 3D reconstructions Crassignatha seeliam sp. nov. male pedipalp and habitus [file zookeys-1012-021-s002.zip › Supplementary material 2/Crassignatha_seeliam_palp_ventral_transparency_2_1.jpg]

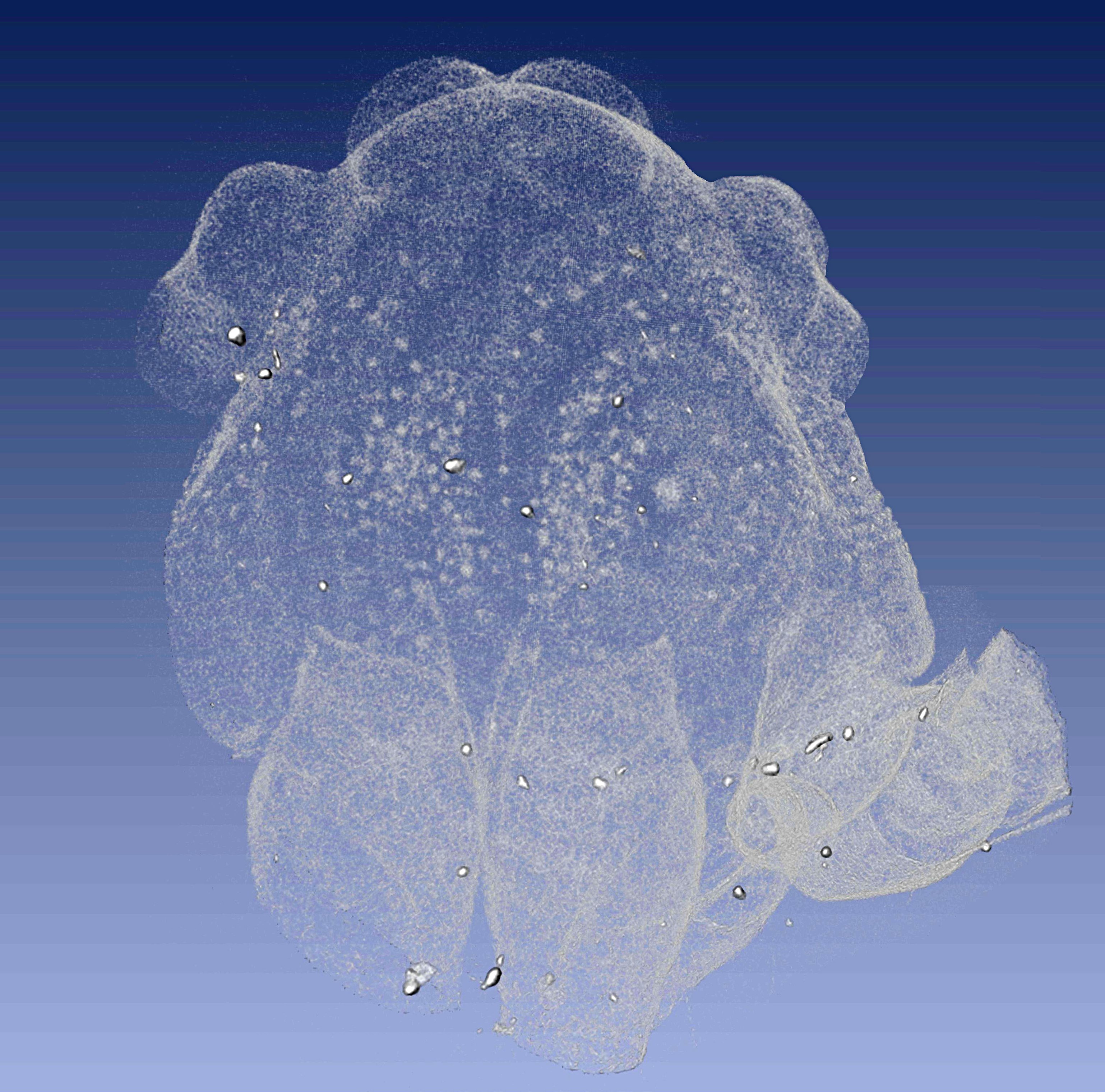

Supplement: Supplementary material 2 — 3D reconstructions Crassignatha seeliam sp. nov. male pedipalp and habitus [file zookeys-1012-021-s002.zip › Supplementary material 2/Crassignatha_seeliam_prosoma_anteior_render.jpg]

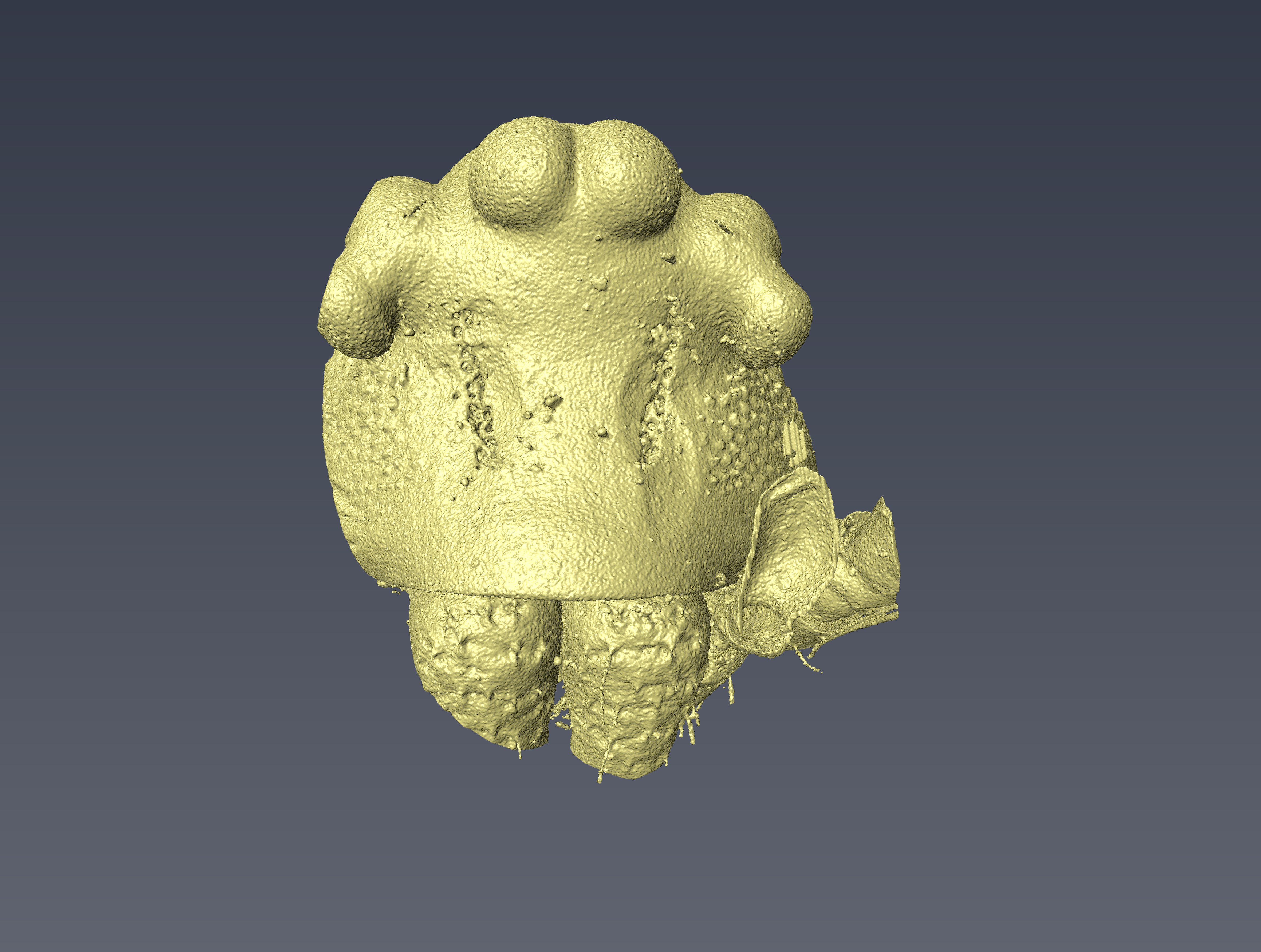

Supplement: Supplementary material 2 — 3D reconstructions Crassignatha seeliam sp. nov. male pedipalp and habitus [file zookeys-1012-021-s002.zip › Supplementary material 2/Crassignatha_seeliam_prosoma_anteior_surface.jpg]

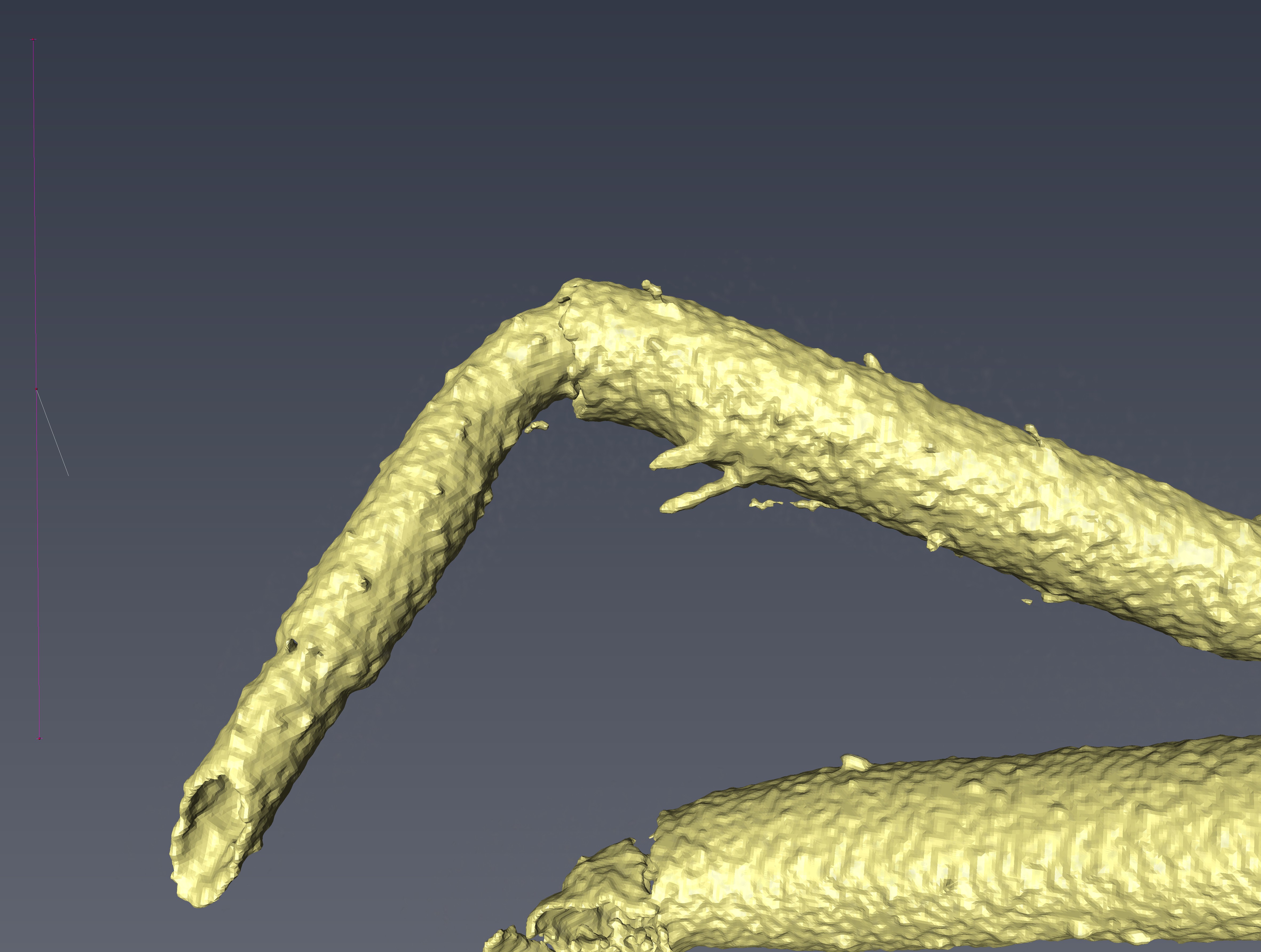

Supplement: Supplementary material 2 — 3D reconstructions Crassignatha seeliam sp. nov. male pedipalp and habitus [file zookeys-1012-021-s002.zip › Supplementary material 2/Crassignatha_seeliam_tibia_leg_IIprolateral_surface.jpg]

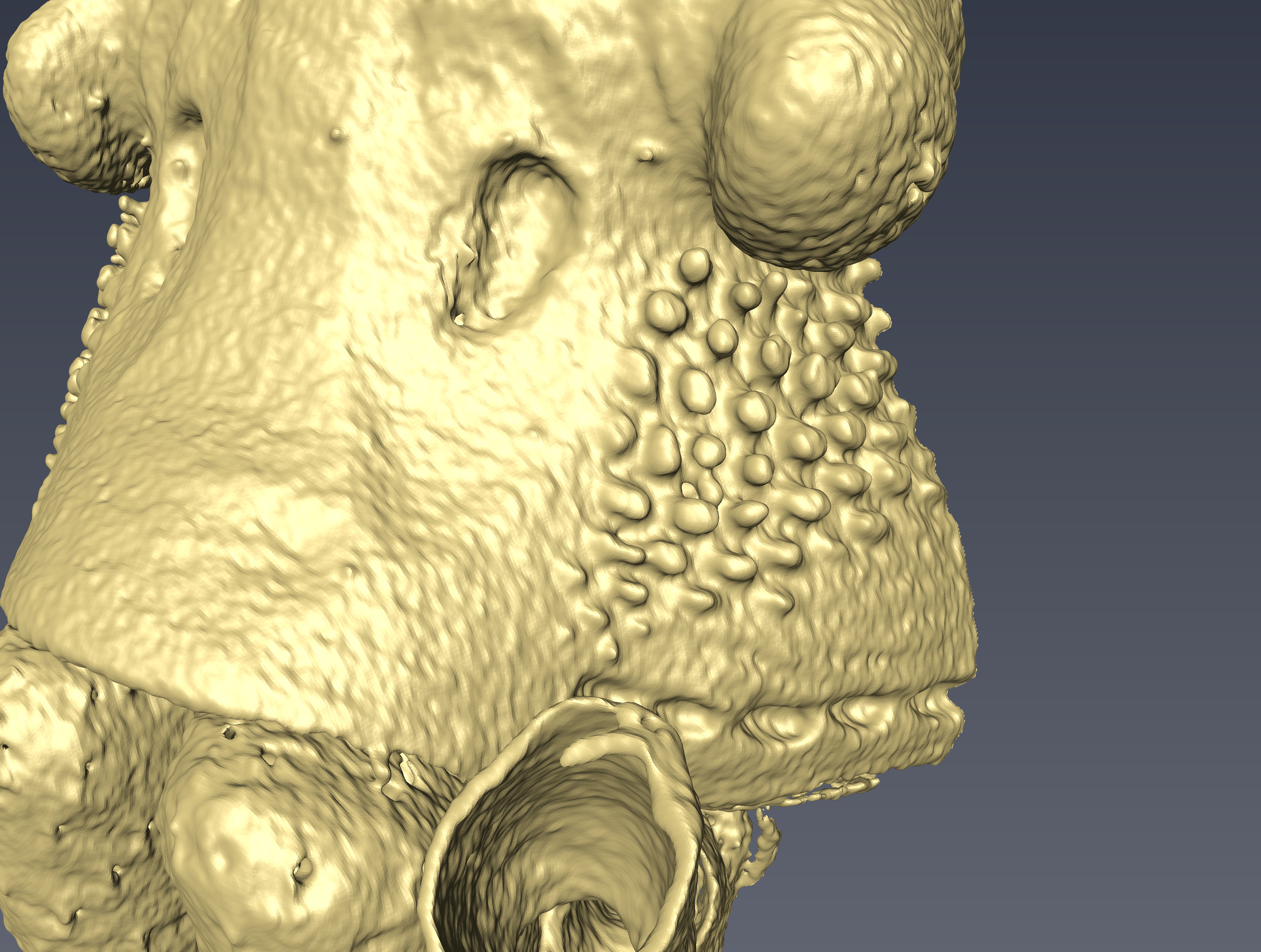

Supplement: Supplementary material 3 — 3D reconstructions Crassignatha danaugirangensis male pedipalp and habitus [file zookeys-1012-021-s003.zip › Supplementary material 3/Crassignatha_danaugirangensis_carapace_antero_lateral_surface.jpg]

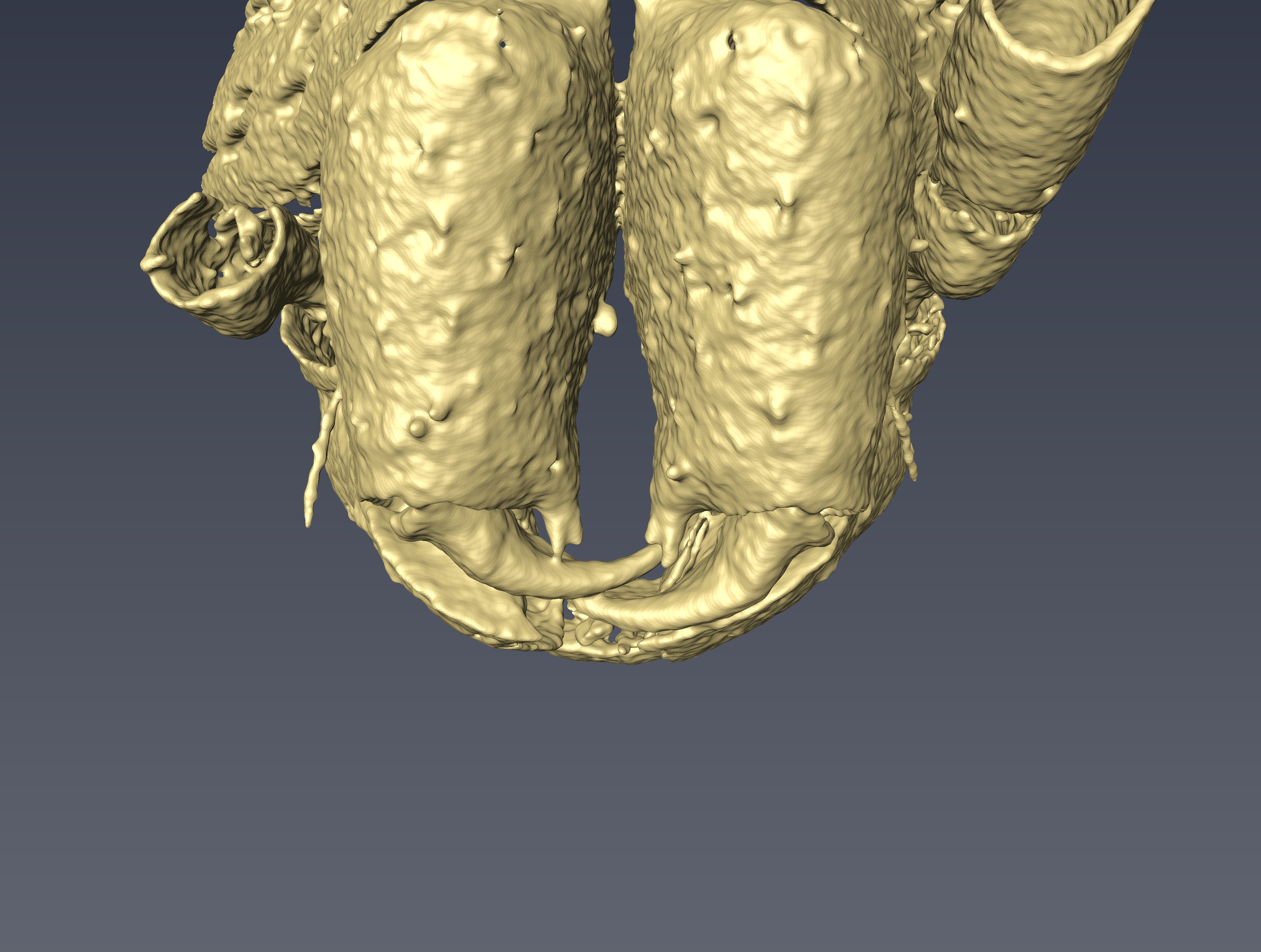

Supplement: Supplementary material 3 — 3D reconstructions Crassignatha danaugirangensis male pedipalp and habitus [file zookeys-1012-021-s003.zip › Supplementary material 3/Crassignatha_danaugirangensis_chelicerae_anteior_surface.jpg]

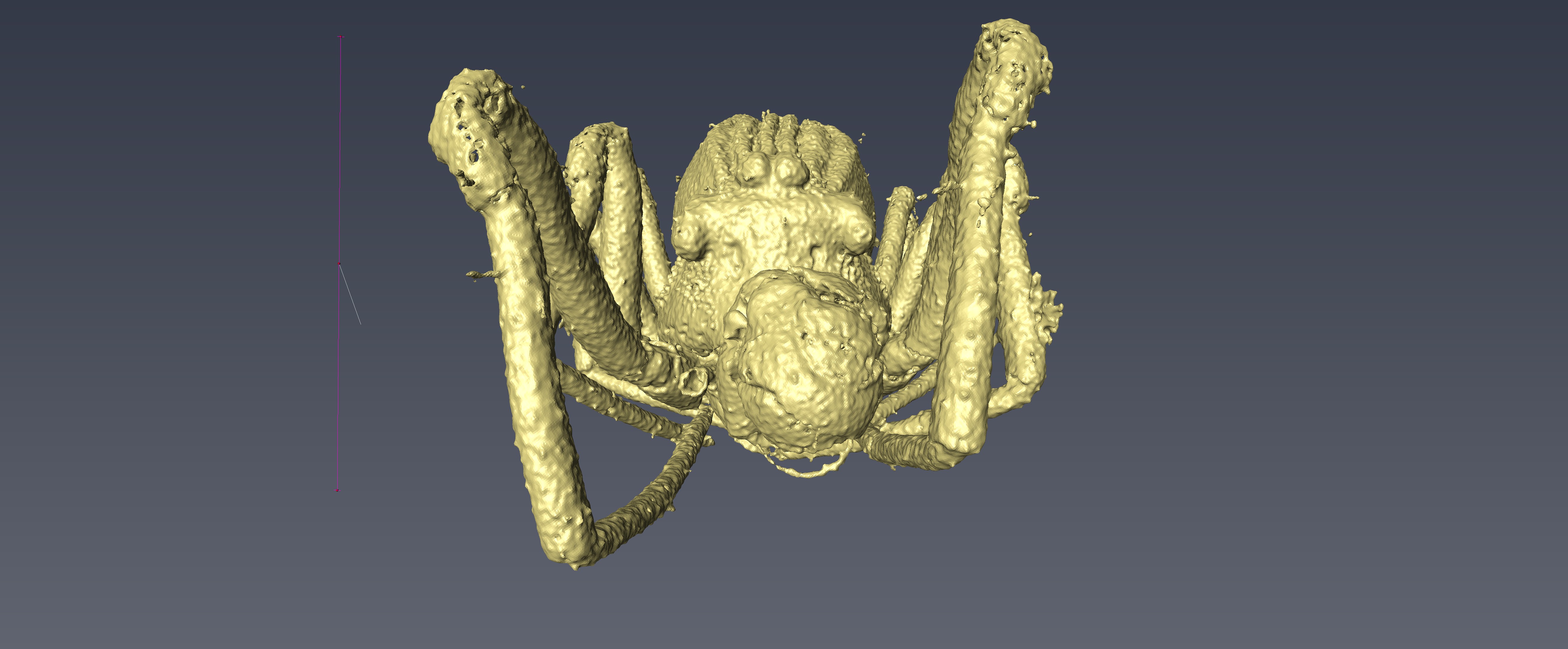

Supplement: Supplementary material 3 — 3D reconstructions Crassignatha danaugirangensis male pedipalp and habitus [file zookeys-1012-021-s003.zip › Supplementary material 3/Crassignatha_danaugirangensis_habitus_anterior_surface.jpg]

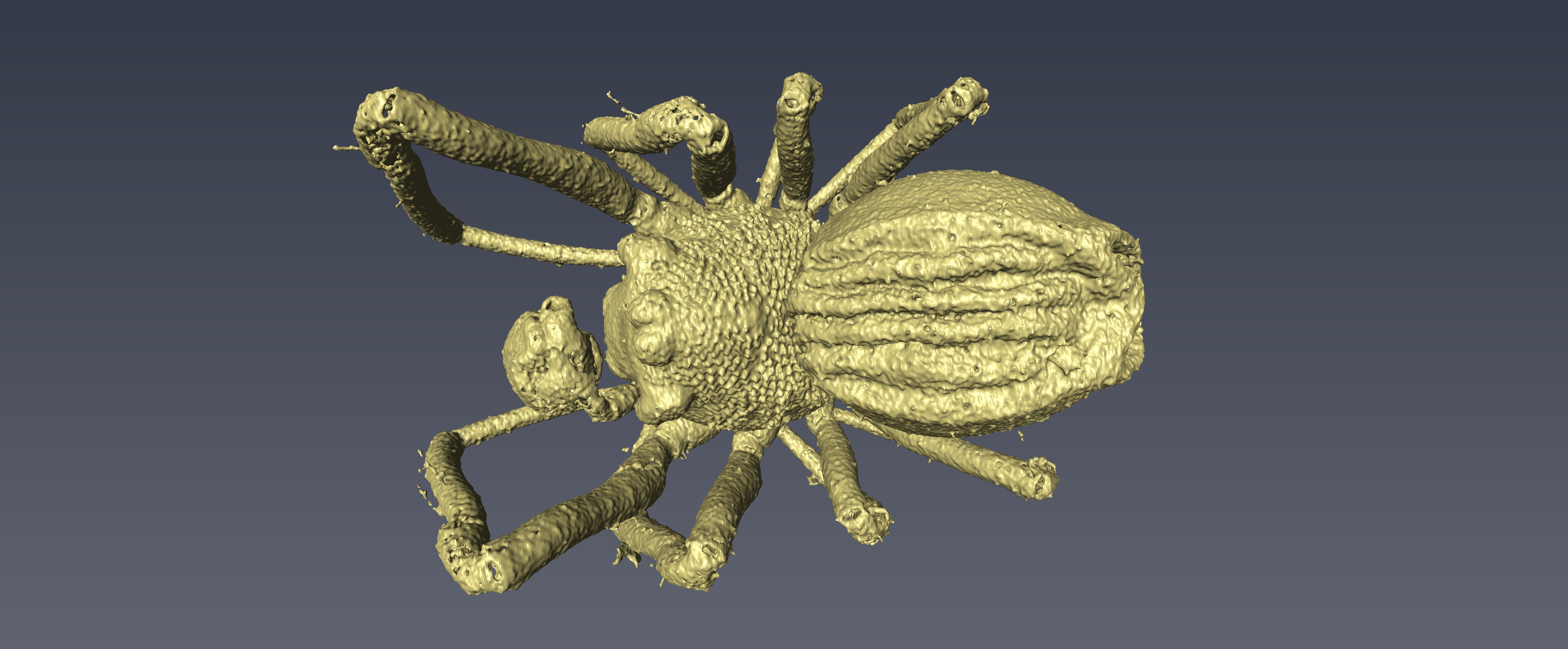

Supplement: Supplementary material 3 — 3D reconstructions Crassignatha danaugirangensis male pedipalp and habitus [file zookeys-1012-021-s003.zip › Supplementary material 3/Crassignatha_danaugirangensis_habitus_dorsal_surface.jpg]

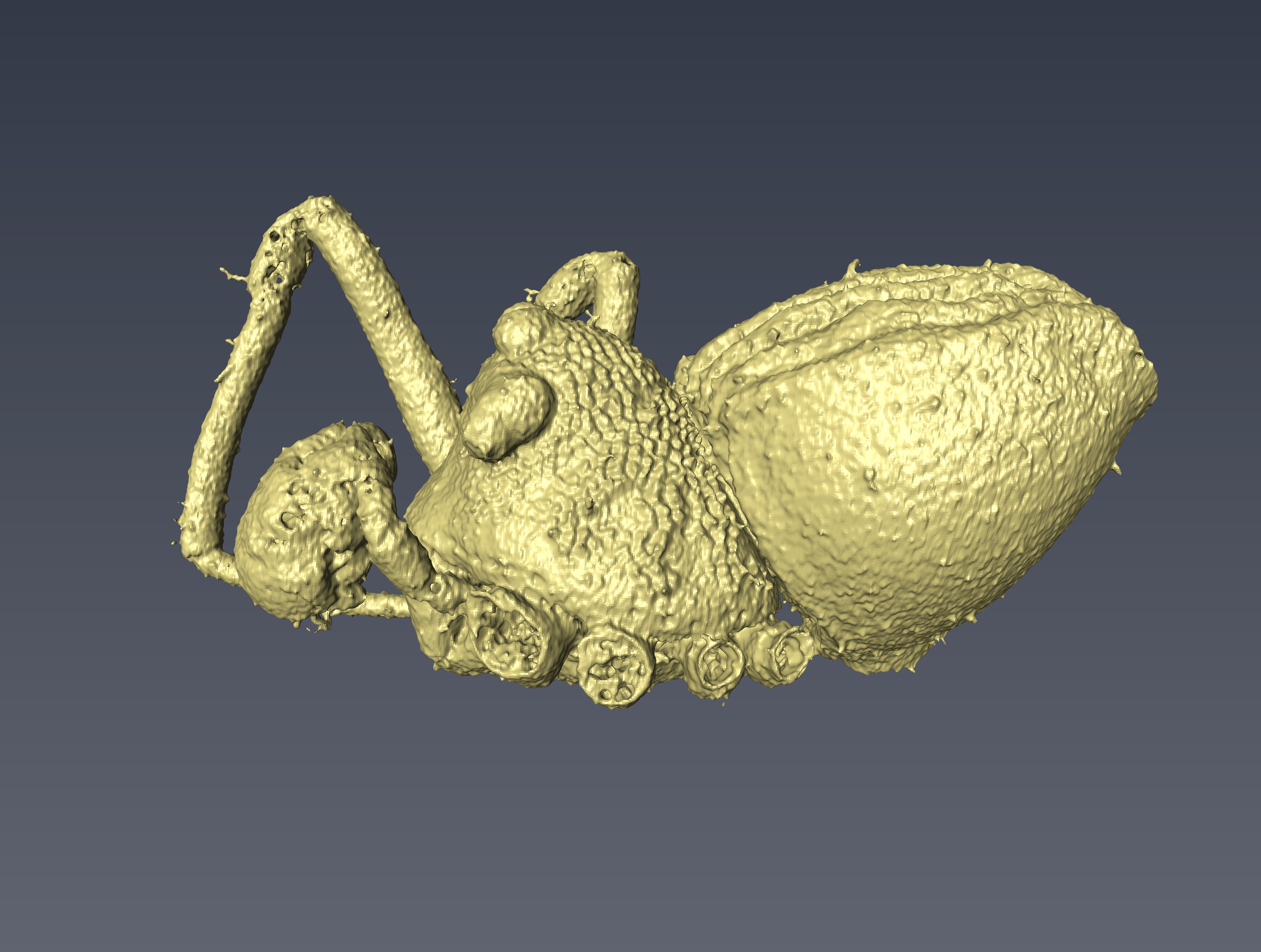

Supplement: Supplementary material 3 — 3D reconstructions Crassignatha danaugirangensis male pedipalp and habitus [file zookeys-1012-021-s003.zip › Supplementary material 3/Crassignatha_danaugirangensis_habitus_lateral_surface.jpg]

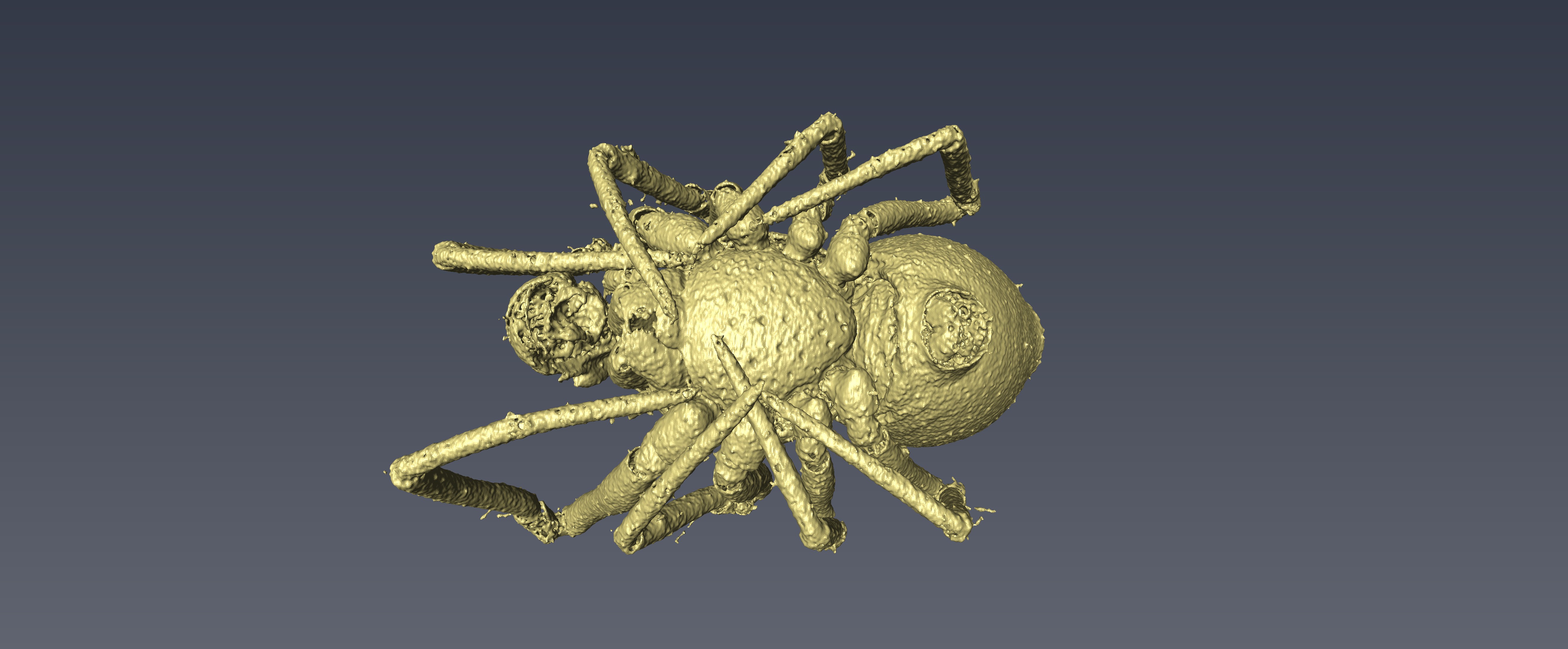

Supplement: Supplementary material 3 — 3D reconstructions Crassignatha danaugirangensis male pedipalp and habitus [file zookeys-1012-021-s003.zip › Supplementary material 3/Crassignatha_danaugirangensis_habitus_ventral_surface.jpg]

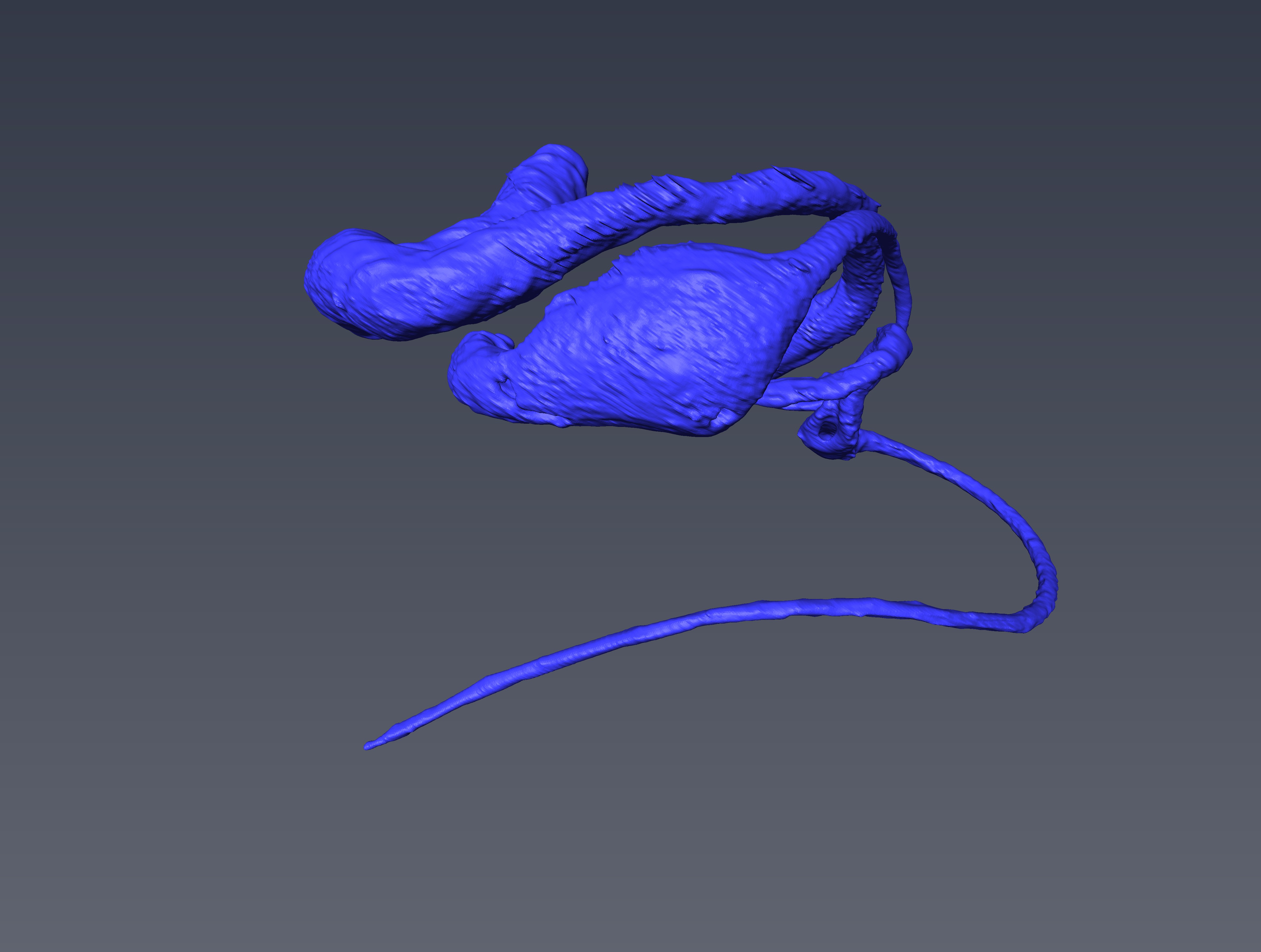

Supplement: Supplementary material 3 — 3D reconstructions Crassignatha danaugirangensis male pedipalp and habitus [file zookeys-1012-021-s003.zip › Supplementary material 3/Crassignatha_danaugirangensis_palp_anterior_ducts.jpg]

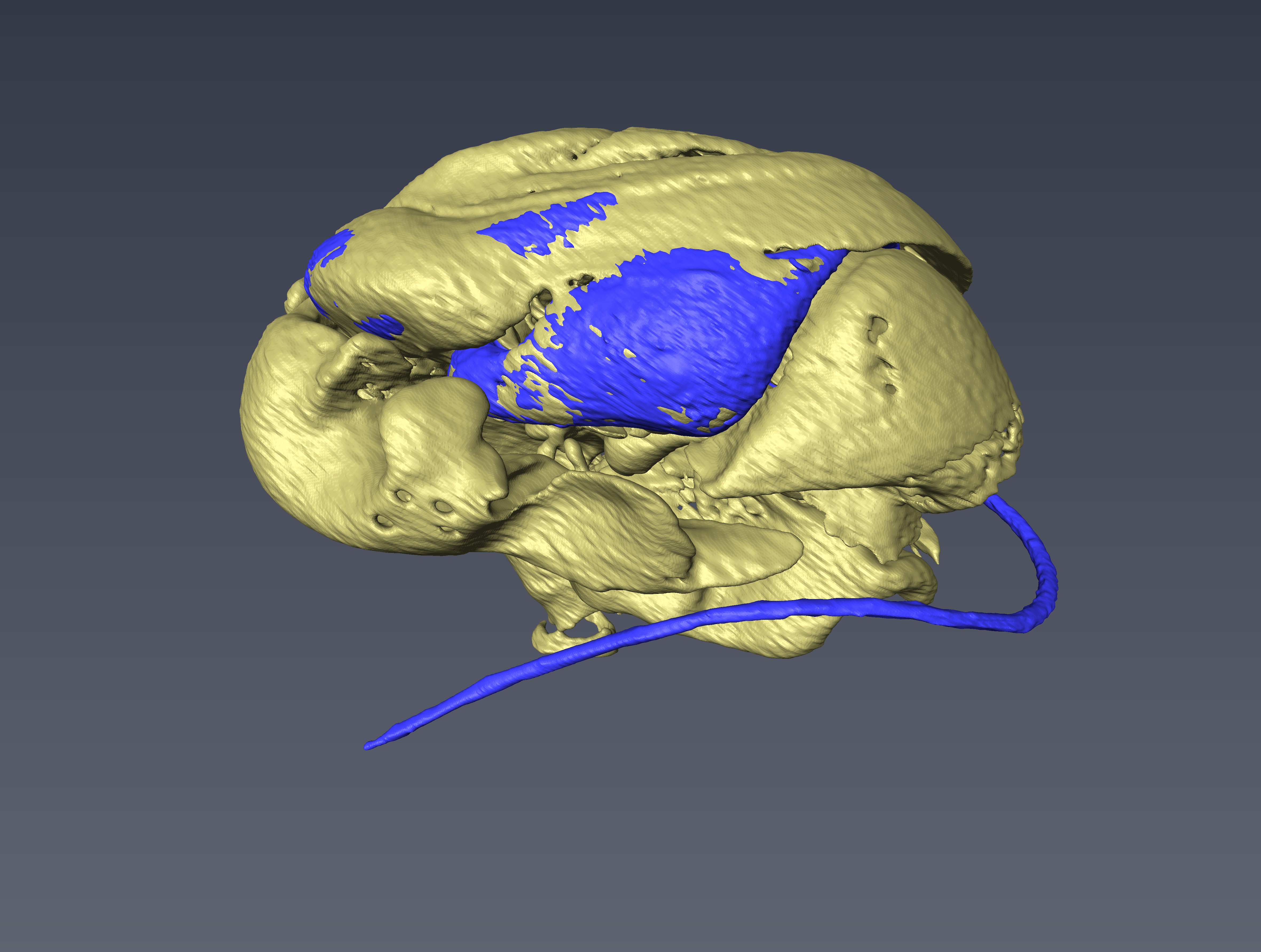

Supplement: Supplementary material 3 — 3D reconstructions Crassignatha danaugirangensis male pedipalp and habitus [file zookeys-1012-021-s003.zip › Supplementary material 3/Crassignatha_danaugirangensis_palp_anterior_surface.jpg]

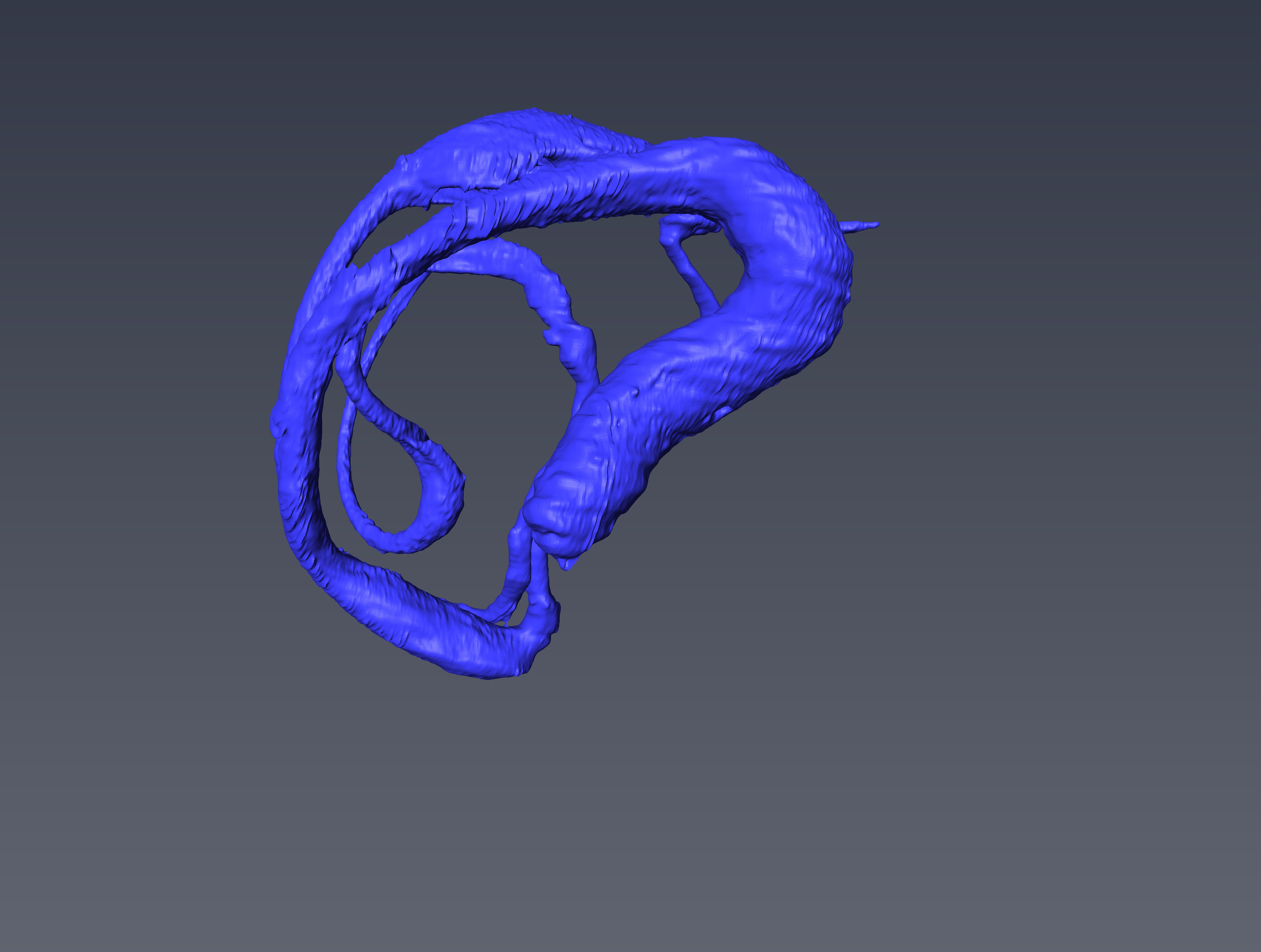

Supplement: Supplementary material 3 — 3D reconstructions Crassignatha danaugirangensis male pedipalp and habitus [file zookeys-1012-021-s003.zip › Supplementary material 3/Crassignatha_danaugirangensis_palp_dorsal_ducts.jpg]

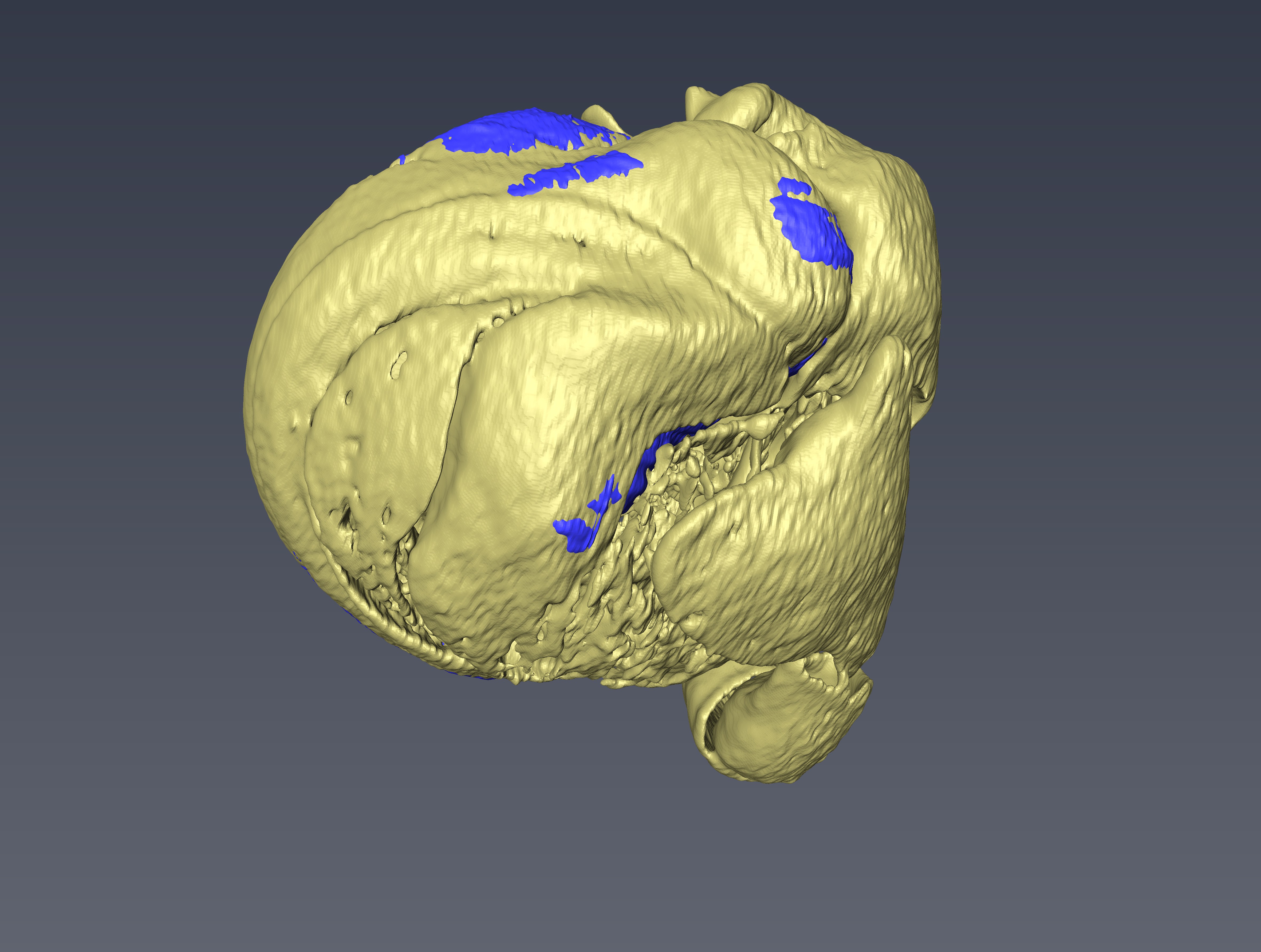

Supplement: Supplementary material 3 — 3D reconstructions Crassignatha danaugirangensis male pedipalp and habitus [file zookeys-1012-021-s003.zip › Supplementary material 3/Crassignatha_danaugirangensis_palp_dorsal_surface.jpg]

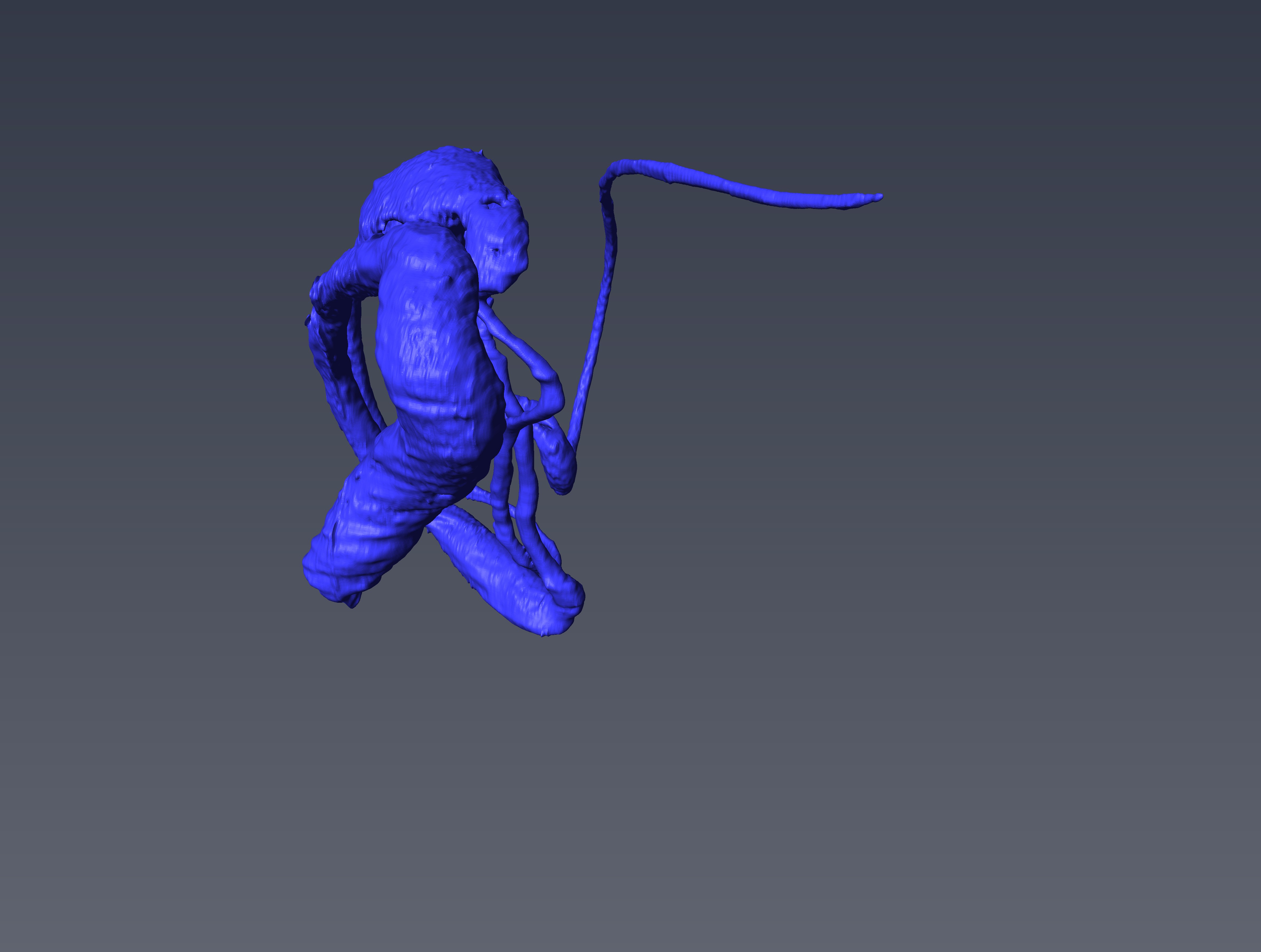

Supplement: Supplementary material 3 — 3D reconstructions Crassignatha danaugirangensis male pedipalp and habitus [file zookeys-1012-021-s003.zip › Supplementary material 3/Crassignatha_danaugirangensis_palp_prolateral_ducts.jpg]

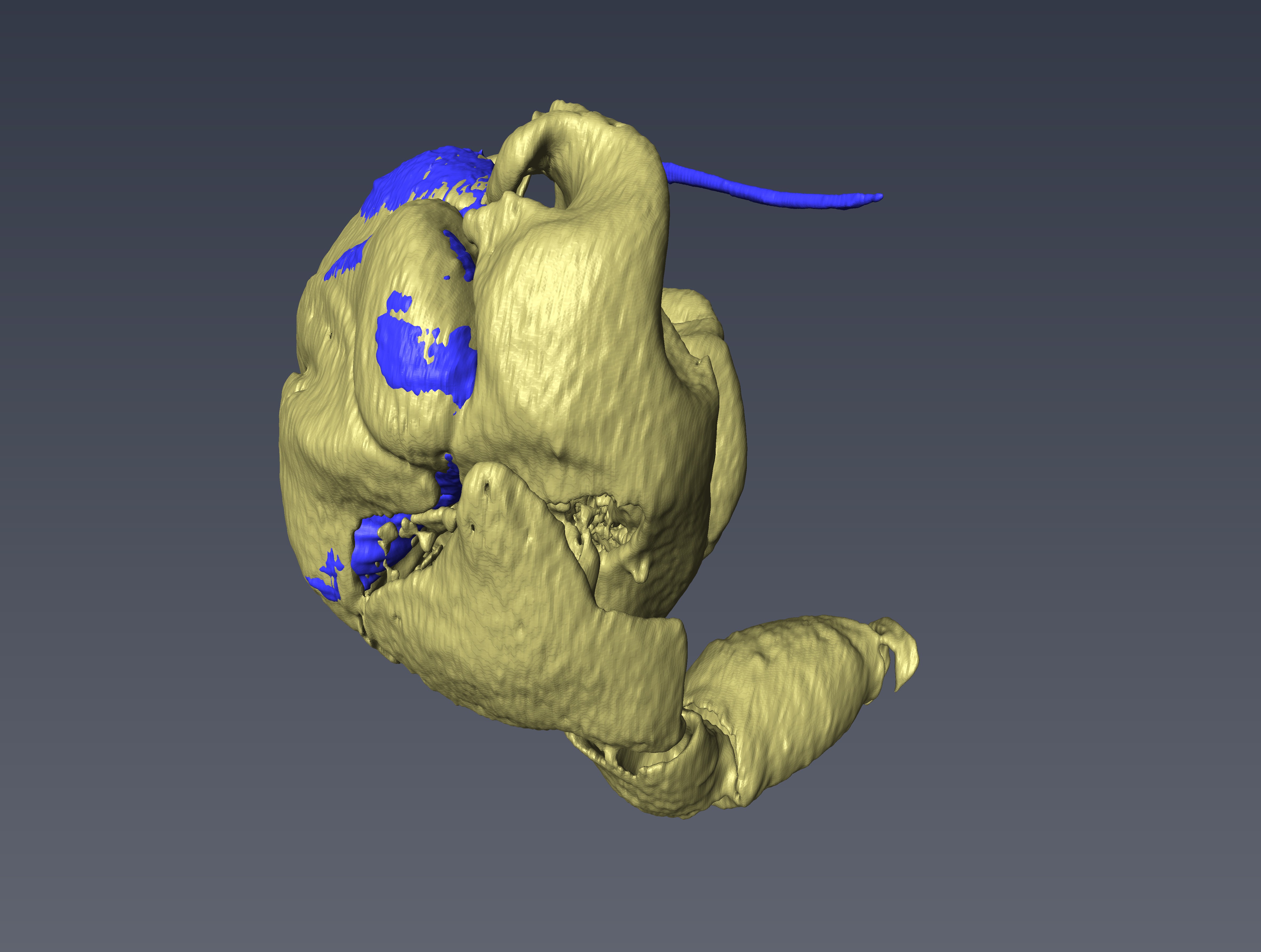

Supplement: Supplementary material 3 — 3D reconstructions Crassignatha danaugirangensis male pedipalp and habitus [file zookeys-1012-021-s003.zip › Supplementary material 3/Crassignatha_danaugirangensis_palp_prolateral_surface.jpg]

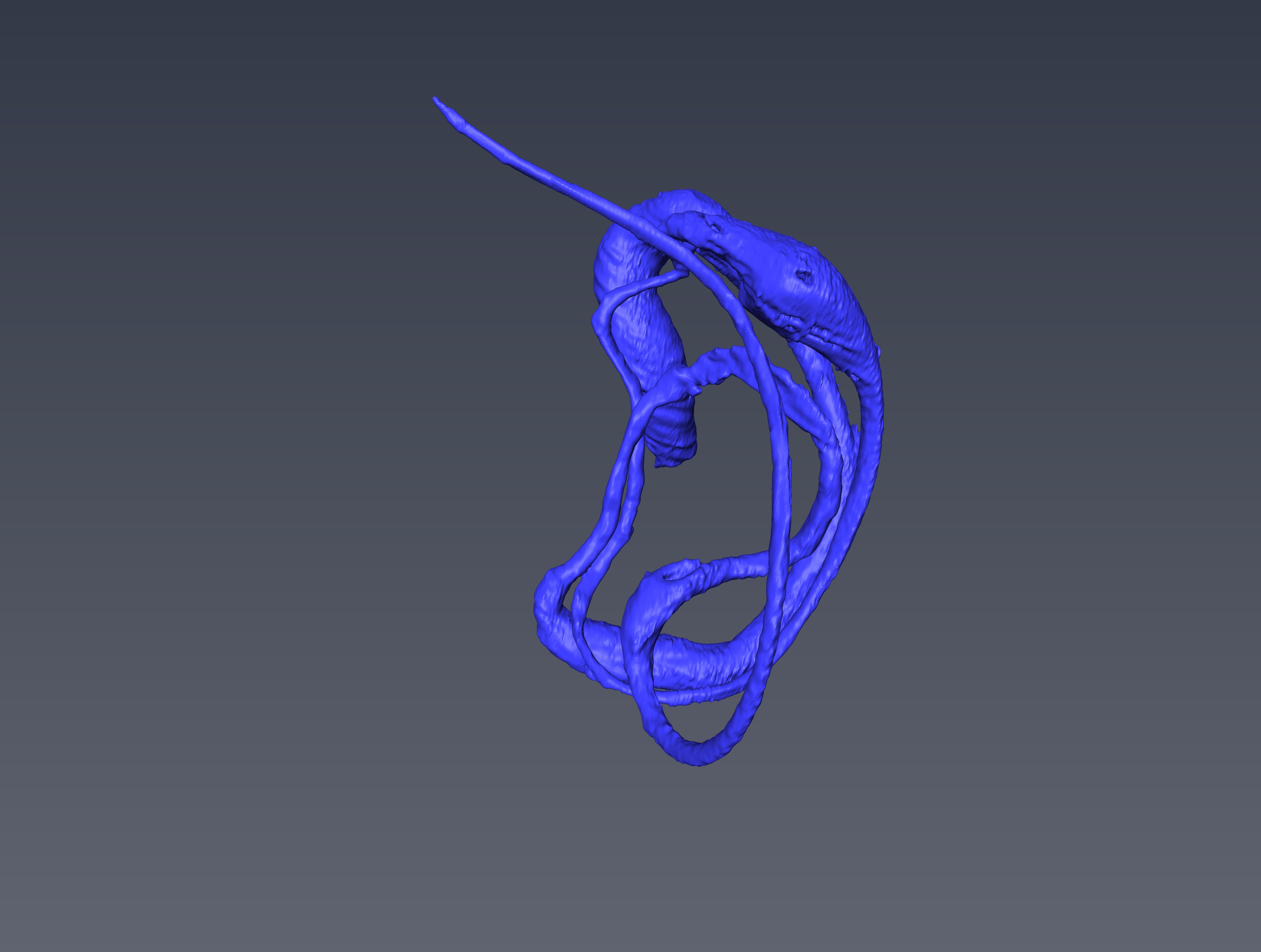

Supplement: Supplementary material 3 — 3D reconstructions Crassignatha danaugirangensis male pedipalp and habitus [file zookeys-1012-021-s003.zip › Supplementary material 3/Crassignatha_danaugirangensis_palp_retrolateral_ducts.jpg]

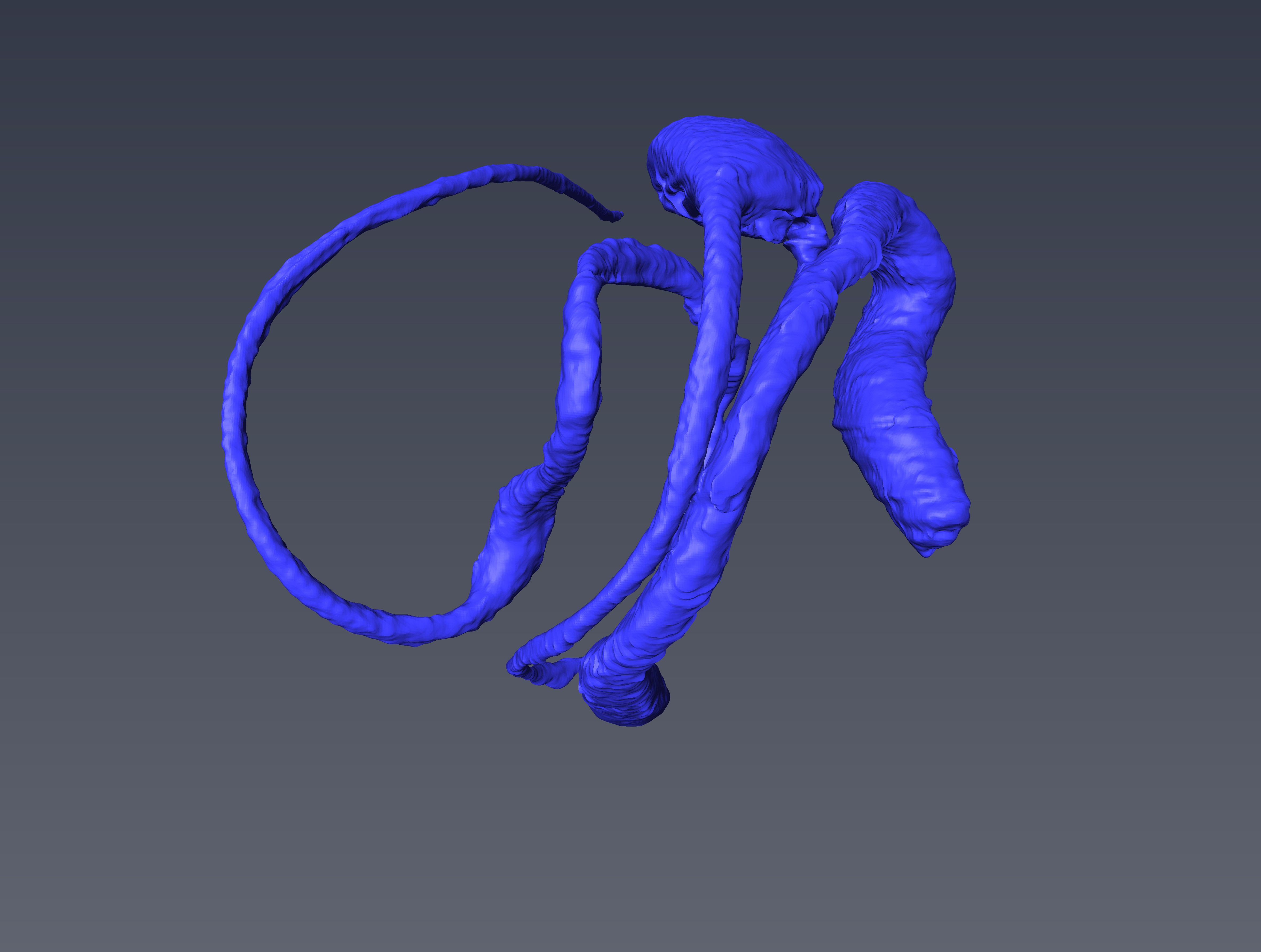

Supplement: Supplementary material 3 — 3D reconstructions Crassignatha danaugirangensis male pedipalp and habitus [file zookeys-1012-021-s003.zip › Supplementary material 3/Crassignatha_danaugirangensis_palp_retrolateral_ducts_1.jpg]

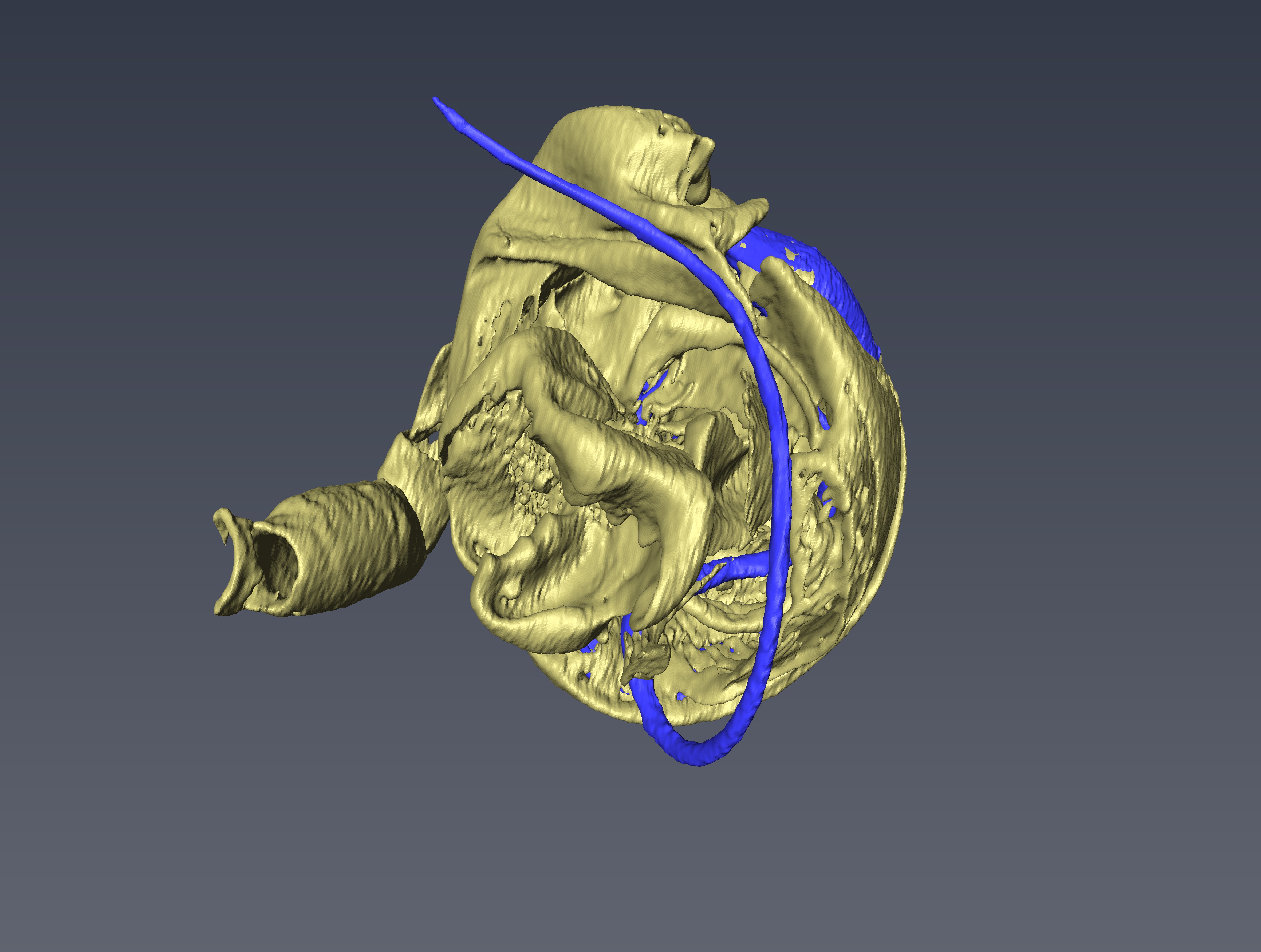

Supplement: Supplementary material 3 — 3D reconstructions Crassignatha danaugirangensis male pedipalp and habitus [file zookeys-1012-021-s003.zip › Supplementary material 3/Crassignatha_danaugirangensis_palp_retrolateral_surface.jpg]

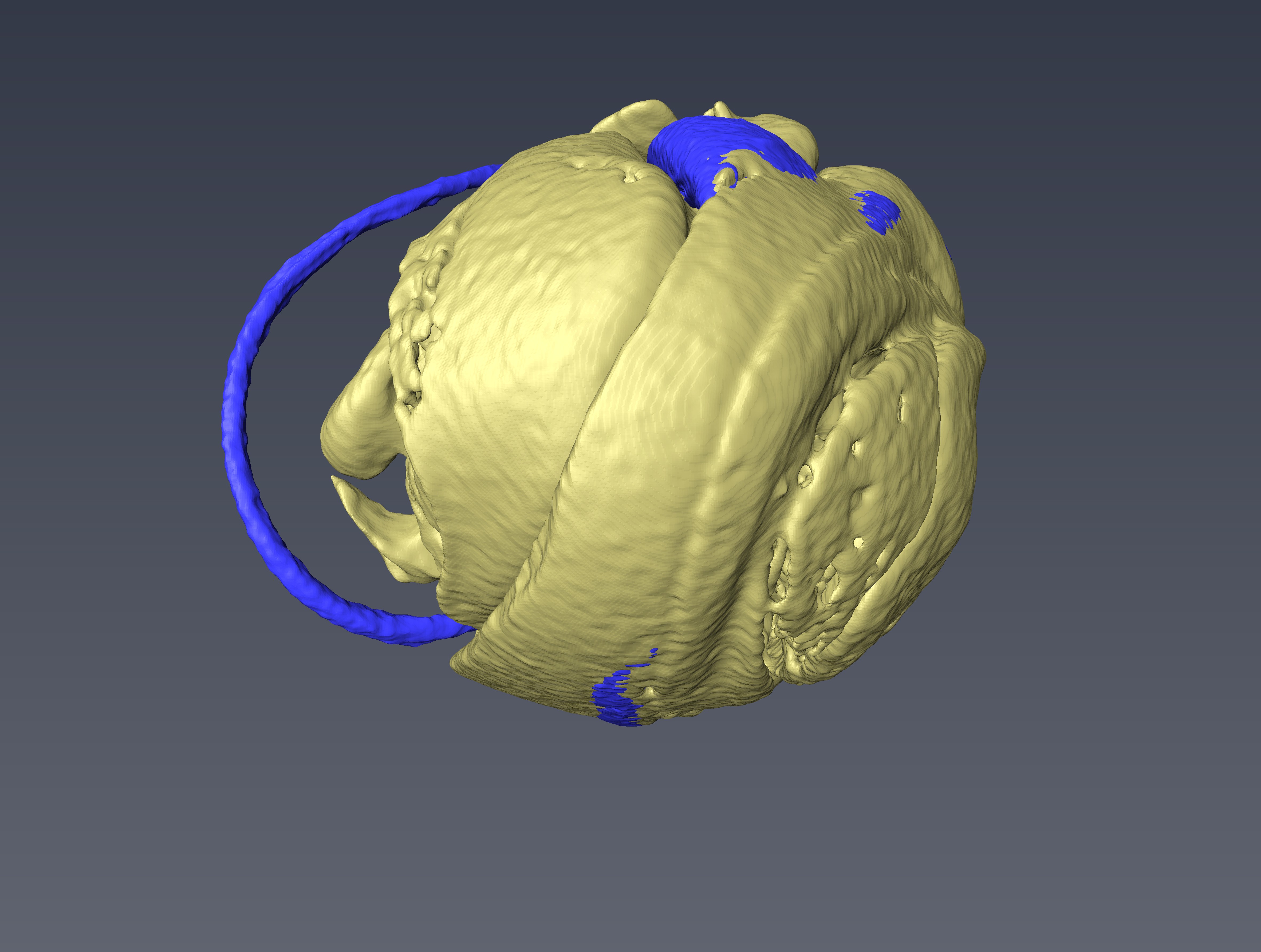

Supplement: Supplementary material 3 — 3D reconstructions Crassignatha danaugirangensis male pedipalp and habitus [file zookeys-1012-021-s003.zip › Supplementary material 3/Crassignatha_danaugirangensis_palp_retrolateral_surface_1.jpg]

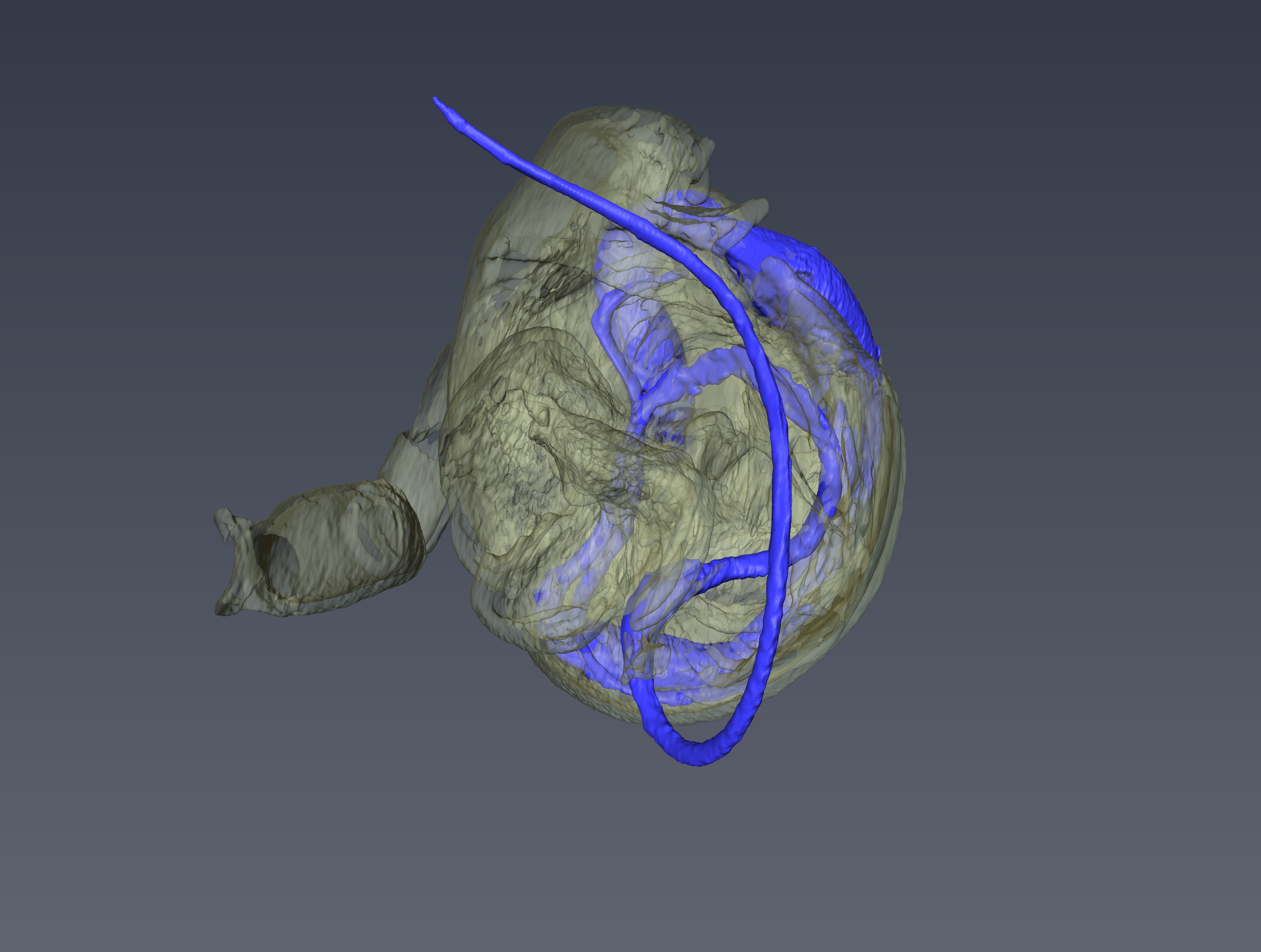

Supplement: Supplementary material 3 — 3D reconstructions Crassignatha danaugirangensis male pedipalp and habitus [file zookeys-1012-021-s003.zip › Supplementary material 3/Crassignatha_danaugirangensis_palp_retrolateral_transparency.jpg]

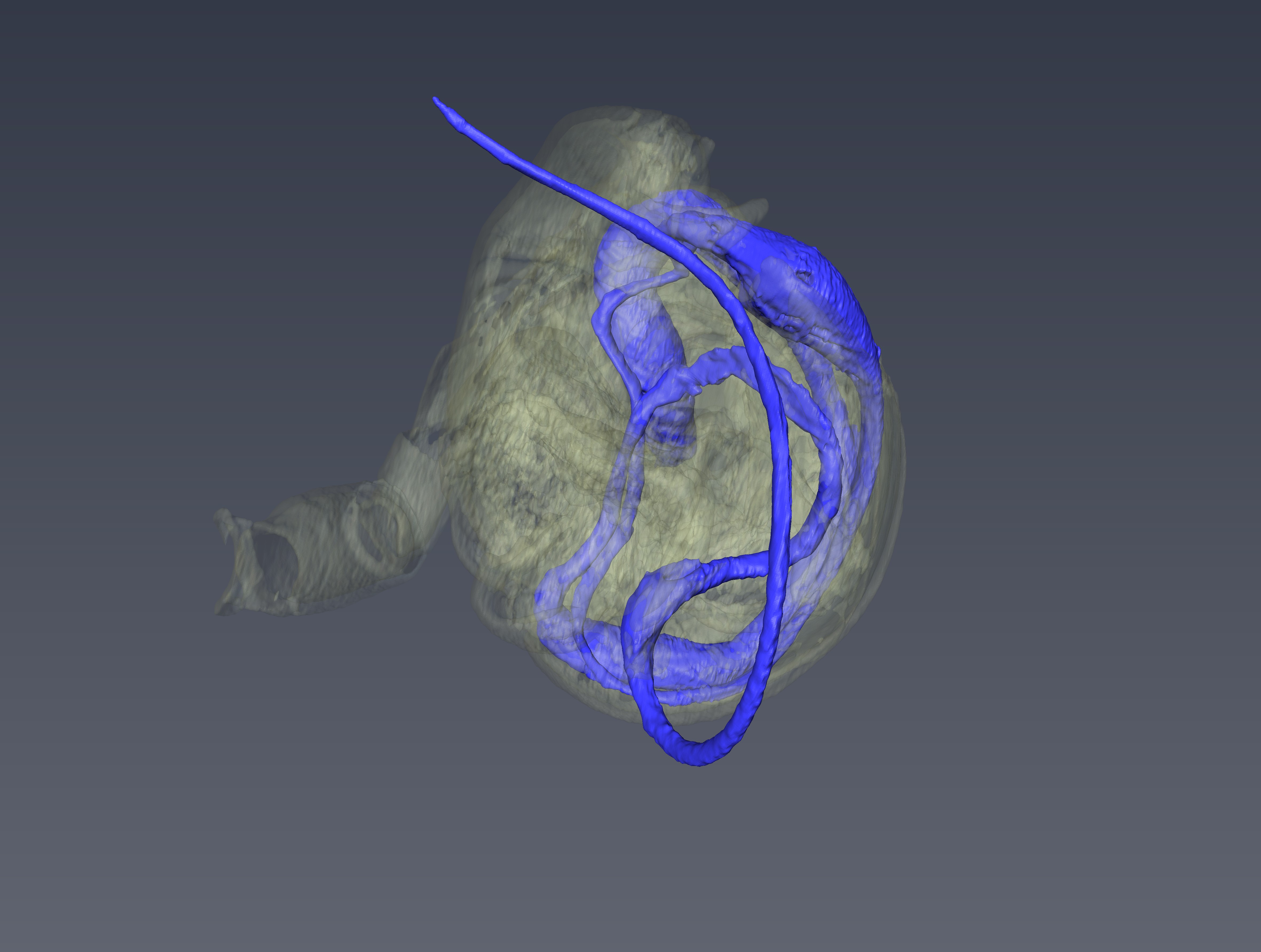

Supplement: Supplementary material 3 — 3D reconstructions Crassignatha danaugirangensis male pedipalp and habitus [file zookeys-1012-021-s003.zip › Supplementary material 3/Crassignatha_danaugirangensis_palp_retrolateral_transparency_2.jpg]

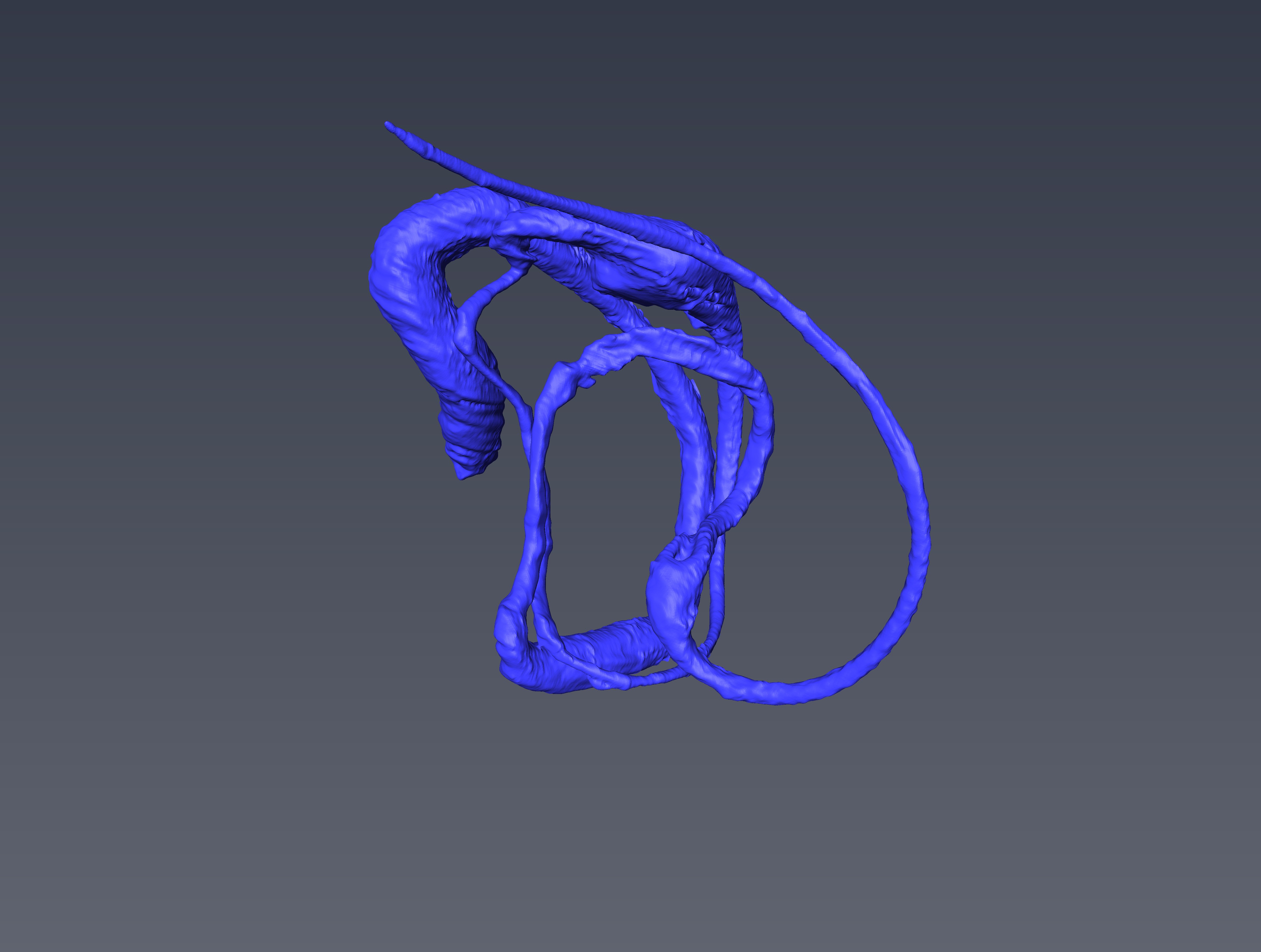

Supplement: Supplementary material 3 — 3D reconstructions Crassignatha danaugirangensis male pedipalp and habitus [file zookeys-1012-021-s003.zip › Supplementary material 3/Crassignatha_danaugirangensis_palp_ventral_ducts.jpg]

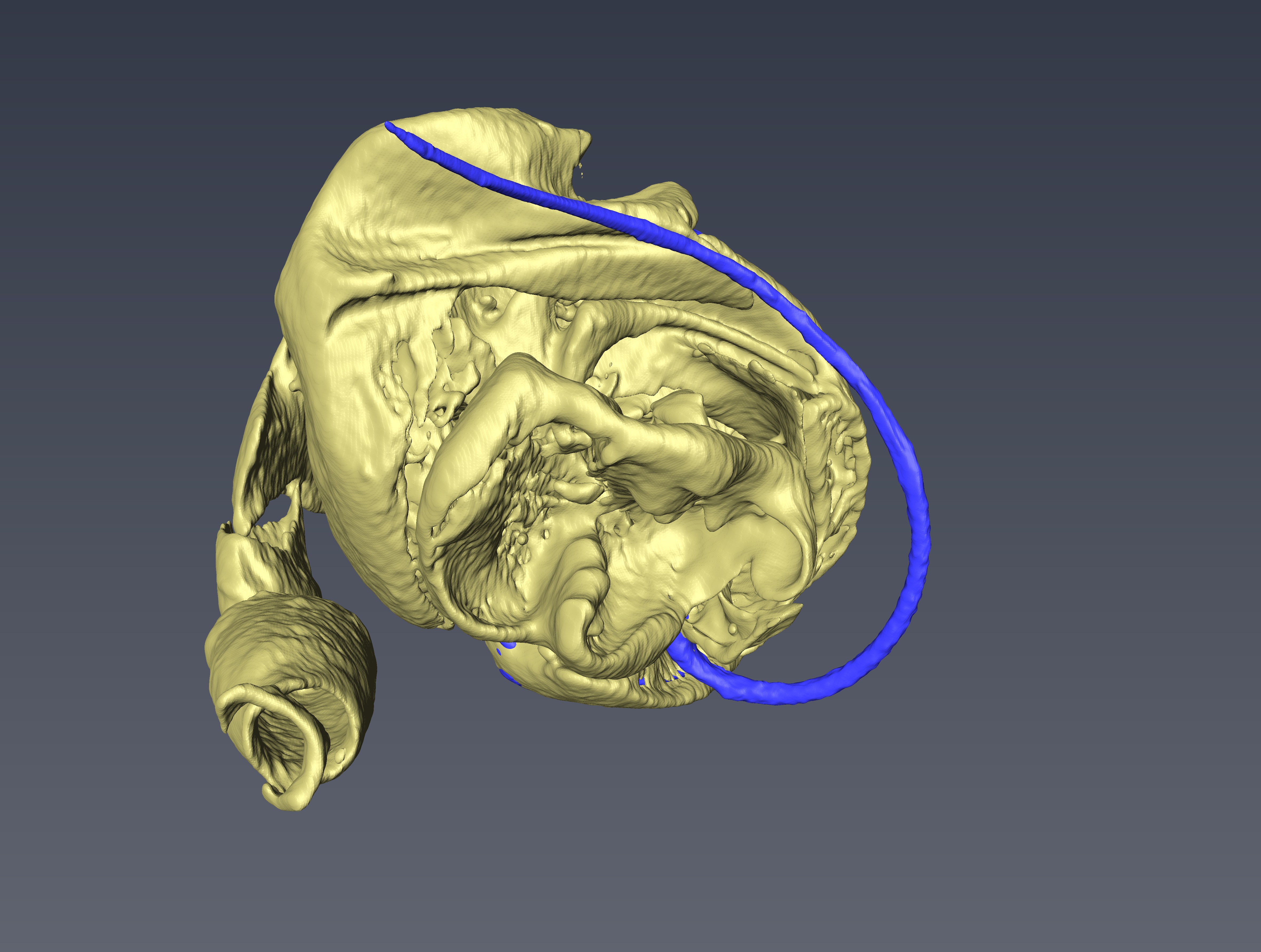

Supplement: Supplementary material 3 — 3D reconstructions Crassignatha danaugirangensis male pedipalp and habitus [file zookeys-1012-021-s003.zip › Supplementary material 3/Crassignatha_danaugirangensis_palp_ventral_surface.jpg]

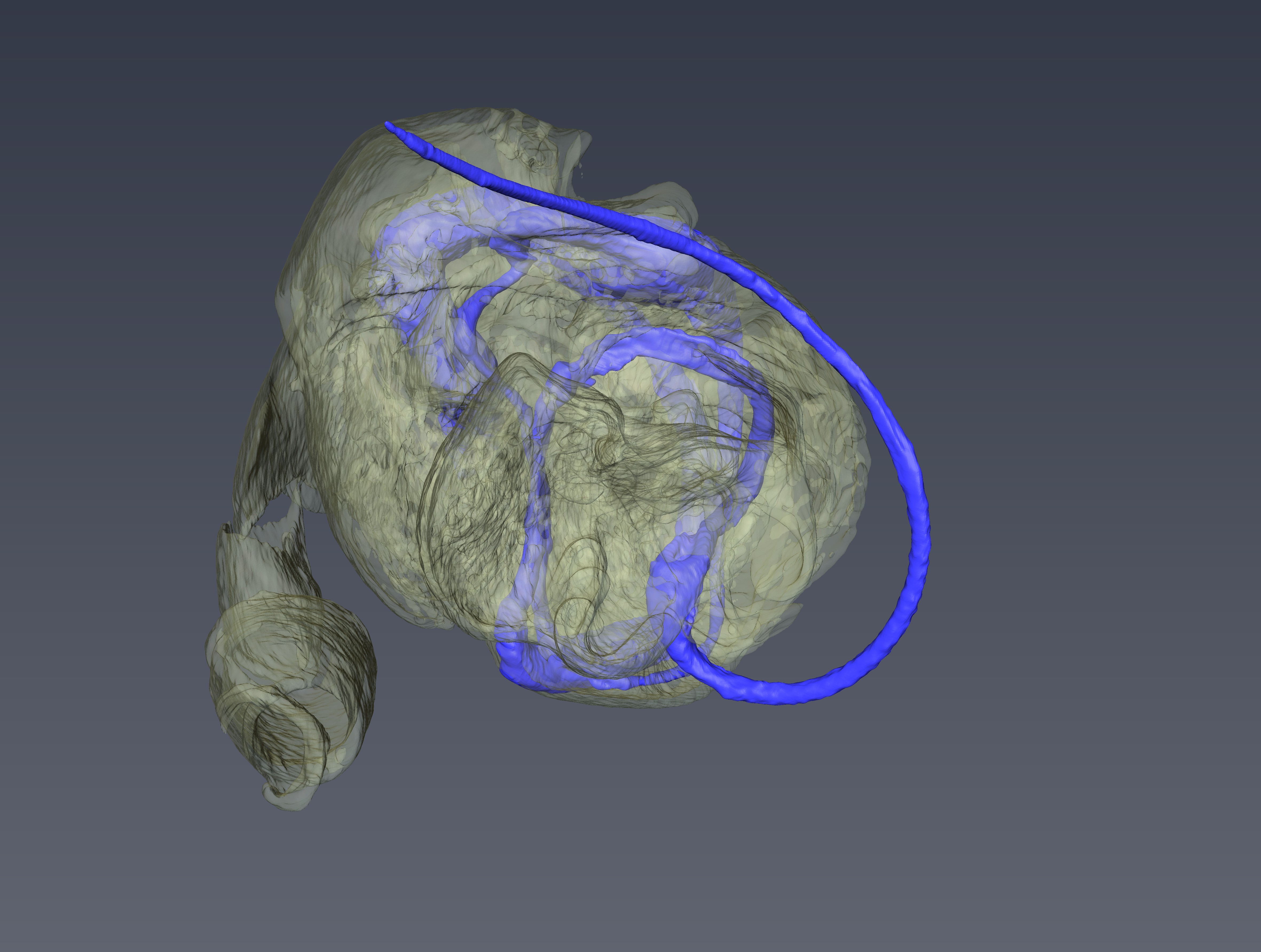

Supplement: Supplementary material 3 — 3D reconstructions Crassignatha danaugirangensis male pedipalp and habitus [file zookeys-1012-021-s003.zip › Supplementary material 3/Crassignatha_danaugirangensis_palp_ventral_transparency.jpg]

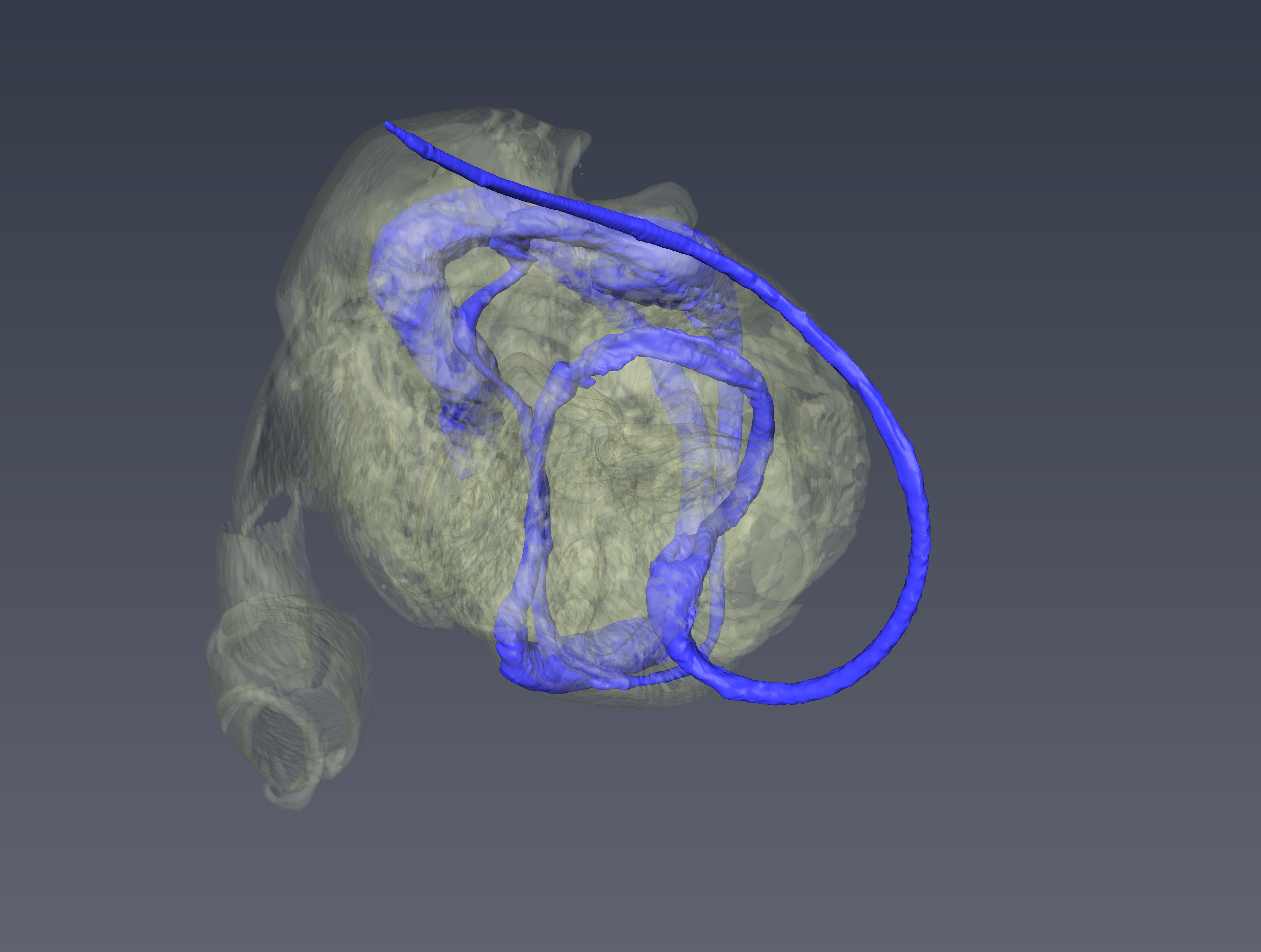

Supplement: Supplementary material 3 — 3D reconstructions Crassignatha danaugirangensis male pedipalp and habitus [file zookeys-1012-021-s003.zip › Supplementary material 3/Crassignatha_danaugirangensis_palp_ventral_transparency_2.jpg]

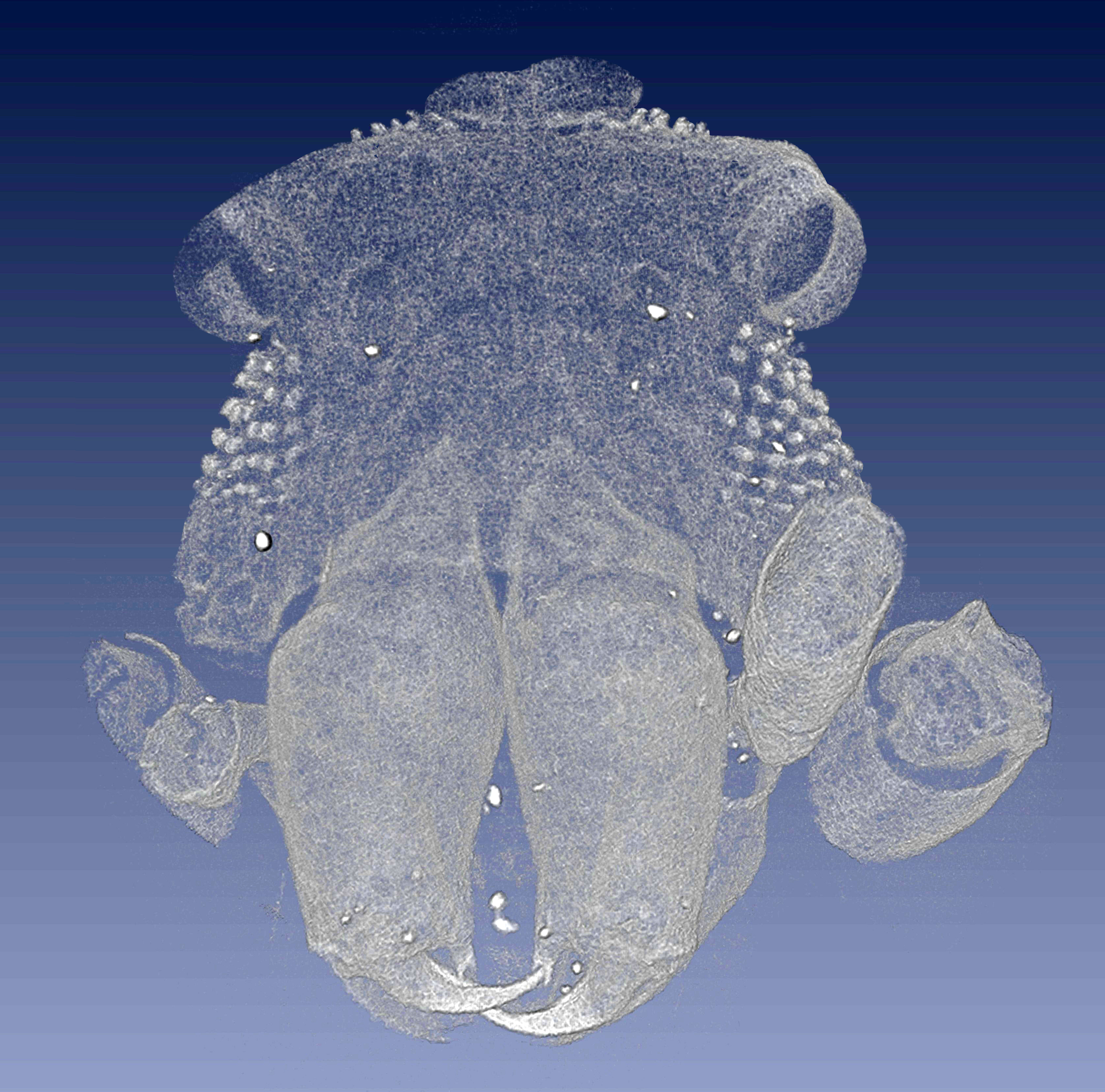

Supplement: Supplementary material 3 — 3D reconstructions Crassignatha danaugirangensis male pedipalp and habitus [file zookeys-1012-021-s003.zip › Supplementary material 3/Crassignatha_danaugirangensis_prosoma_anteior_render.jpg]

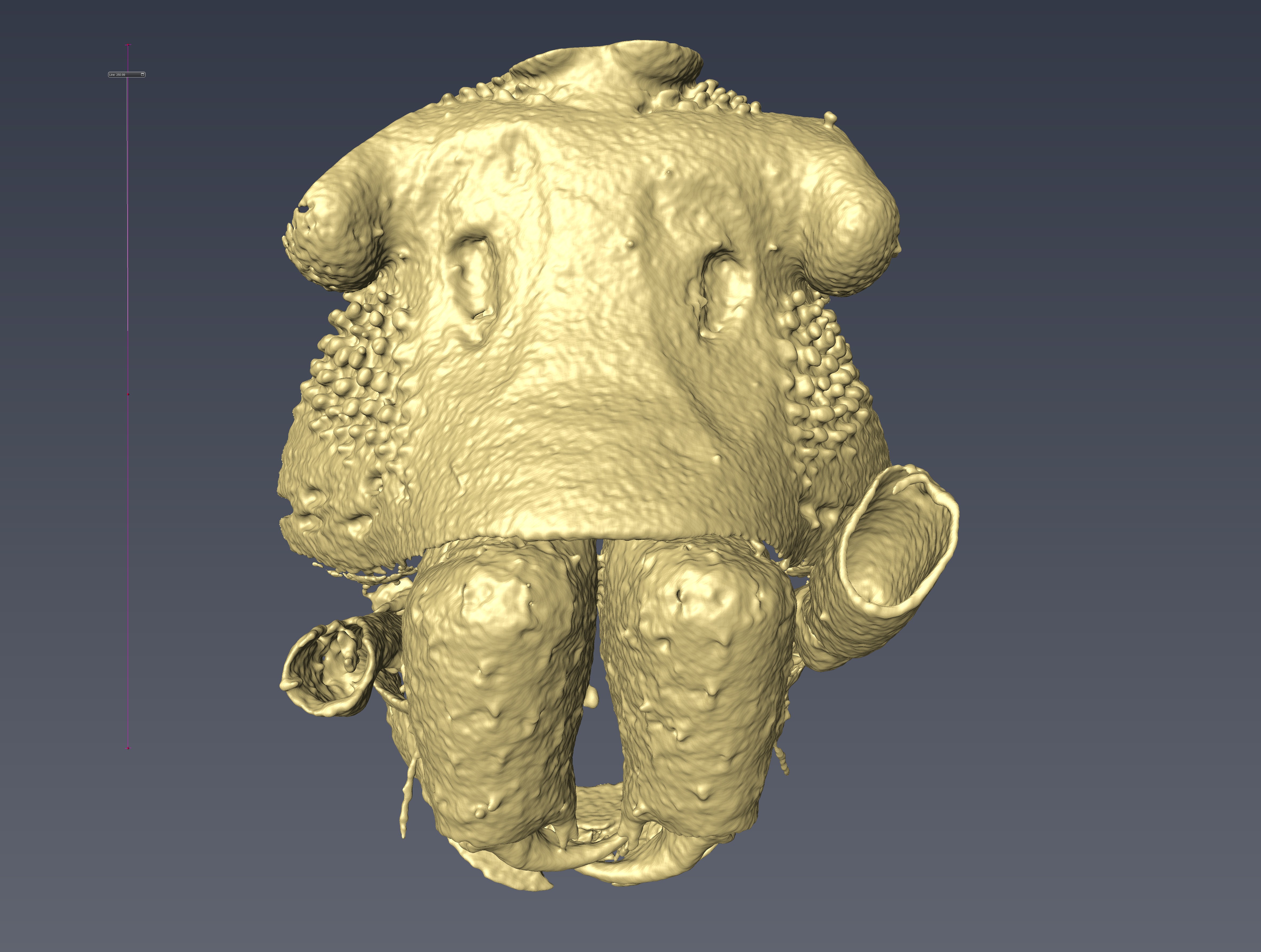

Supplement: Supplementary material 3 — 3D reconstructions Crassignatha danaugirangensis male pedipalp and habitus [file zookeys-1012-021-s003.zip › Supplementary material 3/Crassignatha_danaugirangensis_prosoma_anteior_surface.jpg]

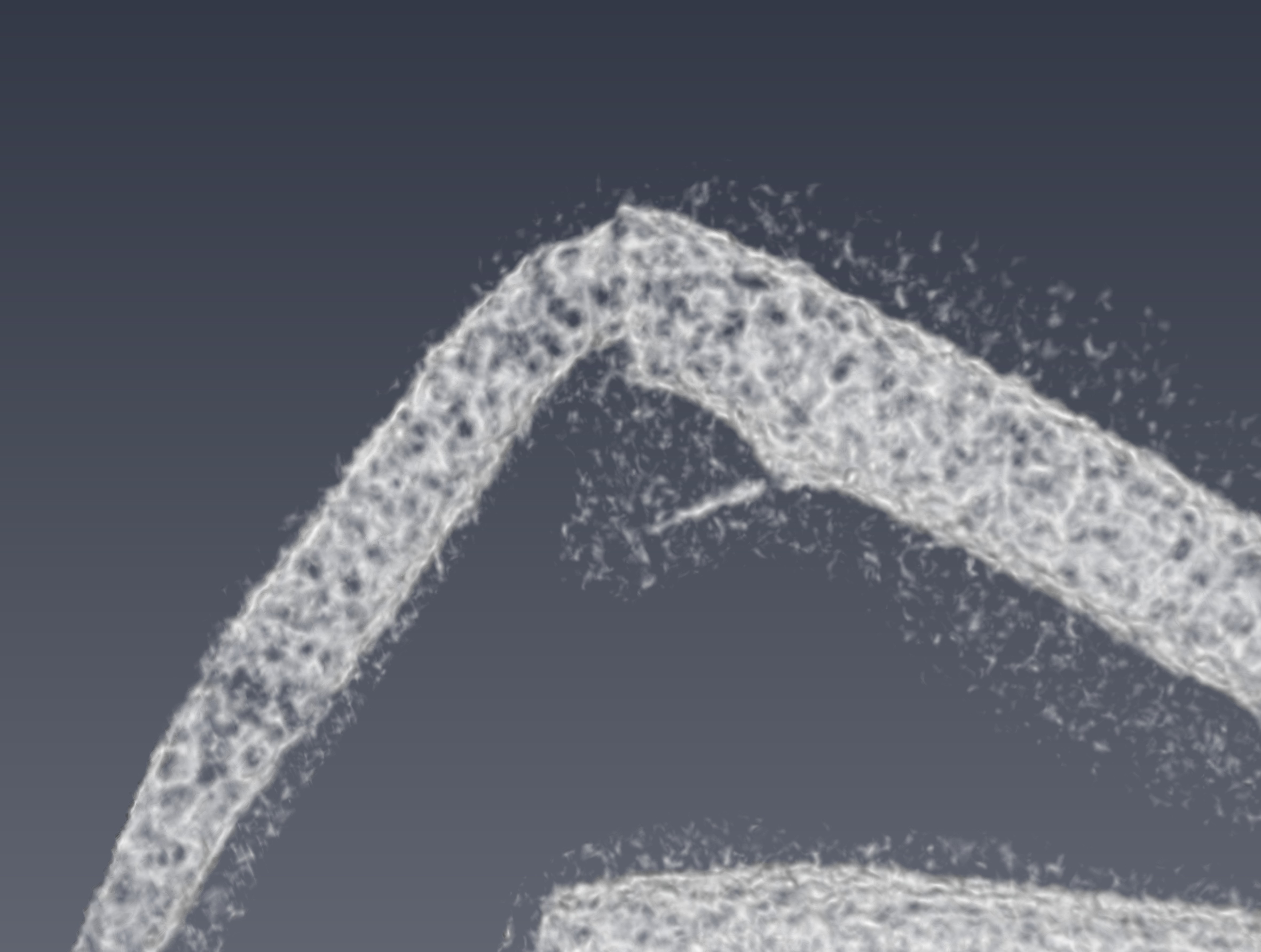

Supplement: Supplementary material 3 — 3D reconstructions Crassignatha danaugirangensis male pedipalp and habitus [file zookeys-1012-021-s003.zip › Supplementary material 3/Crassignatha_danaugirangensis_tibia_leg_II_prolateral_render.jpg]

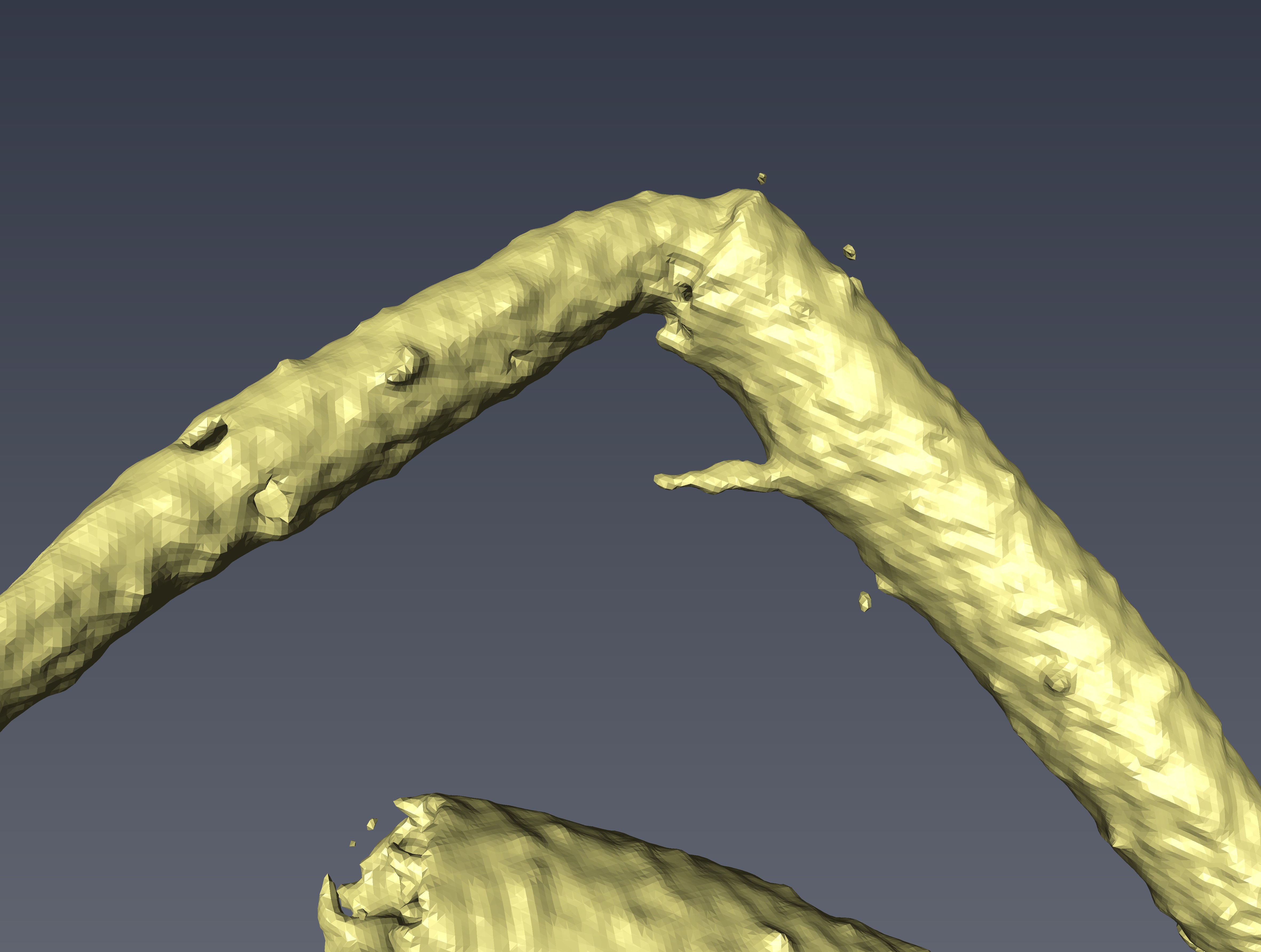

Supplement: Supplementary material 3 — 3D reconstructions Crassignatha danaugirangensis male pedipalp and habitus [file zookeys-1012-021-s003.zip › Supplementary material 3/Crassignatha_danaugirangensis_tibia_leg_II_prolateral_surface.jpg]
